# Proteomic study revealed cellular assembly and lipid metabolism dysregulation in sepsis secondary to community-acquired pneumonia

**DOI:** 10.1038/s41598-017-15755-1

**Published:** 2017-11-15

**Authors:** Narendra Kumar Sharma, Alexandre Keiji Tashima, Milena Karina Colo Brunialti, Eden Ramalho Ferreira, Ricardo Jose Soares Torquato, Renato Arruda Mortara, Flavia Ribeiro Machado, Murillo Assuncao, Otelo Rigato, Reinaldo Salomao

**Affiliations:** 1 Division of Infectious Diseases, Escola Paulista de Medicina, Hospital São Paulo, Universidade Federal de Sao Paulo, Sao Paulo, 04039-032 Brazil; 2 Departamento de Biochemistry, Escola Paulista de Medicina, Universidade Federal de São Paulo, São Paulo, São Paulo, 04023-900 Brazil; 3 Department of Microbiology, Immunology and Parasitology, Escola Paulista de Medicina, Universidade Federal de Sao Paulo, Sao Paulo, 04023-062 Brazil; 4 Intensive Care Unit, Hospital São Paulo, Escola Paulista de Medicina, Universidade Federal de Sao Paulo, Sao Paulo, 04024-002 Brazil; 5 Intensive Care Unit, Hospital Israelita Albert Einstein, Sao Paulo, 05652- 900 Brazil; 6 Intensive Care Unit, Hospital Sirio Libanes, Sao Paulo, 01409-001 Brazil

## Abstract

Sepsis is a life-threatening disorder characterized by organ dysfunction and a major cause of mortality worldwide. The major challenge in studying sepsis is its diversity in such factors as age, source of infection and etiology. Recently, genomic and proteomic approaches have improved our understanding of its complex pathogenesis. In the present study, we use quantitative proteomics to evaluate the host proteome response in septic patients secondary to community-acquired pneumonia (CAP). Samples obtained at admission and after 7 days of follow-up were analyzed according to the outcomes of septic patients. The patients’ proteome profiles were compared with age- and gender-matched healthy volunteers. Bioinformatic analyses of differentially expressed proteins showed alteration in the cytoskeleton, cellular assembly, movement, lipid metabolism and immune responses in septic patients. Actin and gelsolin changes were assessed in mononuclear cells using immunofluorescence, and a higher expression of gelsolin and depletion of actin were observed in survivor patients. Regarding lipid metabolism, changes in cholesterol, HDL and apolipoproteins were confirmed using enzymatic colorimetric methods in plasma. Transcriptomic studies revealed a massive change in gene expression in sepsis. Our proteomic results stressed important changes in cellular structure and metabolism, which are possible targets for future interventions of sepsis.

## Introduction

Sepsis is a major cause of morbidity and mortality worldwide. The actual number of cases is unknown, as there is limited information from developing countries. An extrapolation from high-income country data suggests global estimates of 31.5 million sepsis and 19.4 million severe sepsis cases, with potentially 5.3 million deaths^1^. In a recent multicenter study in Brazil, one third of intensive care beds were occupied by septic patients, with a mortality rate of 55.7%^2^. The place of acquisition, e.g., community-acquired or hospital-acquired infections, and the primary source of infection—respiratory tract, gastrointestinal tract, urinary tract, and surgical infections—are related to the etiology, pattern of microbial resistance and outcomes in sepsis^3–5^. Respiratory infection is a leading source of sepsis in ICU patients, accounting for more than 50% of infections^5^.

The concept of sepsis has been revised recently and is currently defined as life-threatening organ dysfunction caused by a dysregulated host response to infection^6^. Thus, sepsis results from a complex interaction between the host and the infecting microorganisms, in which the mechanisms of host defense are involved in the pathophysiology of the syndrome and play a major role in the outcomes^7^. Inflammatory and anti-inflammatory responses are triggered in sepsis, and the predominance of one response over the other during the ongoing infection may lead to the deleterious effects of inflammation or immunosuppression^8,9^. Inflammatory cytokines, such as tumor necrosis factor-α (TNF-α), interleukin (IL)-1, and IL-6, lead to endothelial damage and activation of procoagulation factors, which results in intravascular clotting, the formation of blood clots in small blood vessels, and multiple organ failure^10^. The inflammatory response leads to overwhelming oxidative stress, which results from the uncontrolled production of reactive oxygen species (ROS) and reactive nitrogen species (RNS)^11^. Mitochondrial enzymes are particularly vulnerable to oxidative stress, mainly to peroxynitrite, which leads to the cessation of electron transport and ATP formation, mitochondrial swelling, and permeabilization of the outer mitochondrial membrane^12^.

In recent years, proteomics has emerged as a powerful tool to evaluate the complex host-response to sepsis. This methodology is advantageous for the identification of biomarkers, altered pathways, functional alterations and mechanisms^13,14^. Several groups have investigated the proteome changes in animal models of sepsis as well as in septic patents^13,15^. Proteome studies have investigated the changes induced in human volunteers in response to lipopolysaccharides (LPS)^16^. Interestingly, circulating proteins, such as apolipoprotein, LDL, transferrin and holotransferrin, interact with the bacterial cell wall components - LPS and lipoteichoic acid (LTA) - and modulate their binding and the subsequent induced inflammatory response. Such proteins were found in lower abundance in non-surviving septic patients in one proteomic study^17^.

Few studies have been performed with septic patients, mostly without focusing on a primary source of infection^18–20^. One study evaluated proteome changes in patients with community-acquired pneumonia (CAP). The focus of the investigation was the alterations in the age-related pathways in young and old patients who could correlate with later development of sepsis^21^. In the present study, we evaluated the proteome changes in septic patients, focusing on changes related to immune and metabolic pathways in survivors and non-survivors. Aiming to avoid, at least in part, patient heterogeneity, we selected patients diagnosed with CAP as the source of infection. Samples were obtained at admission and after seven days of therapy to measure changes after the initial interventions. Using the absolute quantitative method iTRAQ, we were able to identify several differentially expressed proteins in septic patients compared to healthy volunteers and the associated outcomes. We used a bioinformatics tool to identify altered functions, pathways and regulatory networks. Our study provides evidence for a compromised cytoskeleton, cell movement, the transport of macromolecules and energy metabolism in sepsis.

## Results

**Figure 1:**
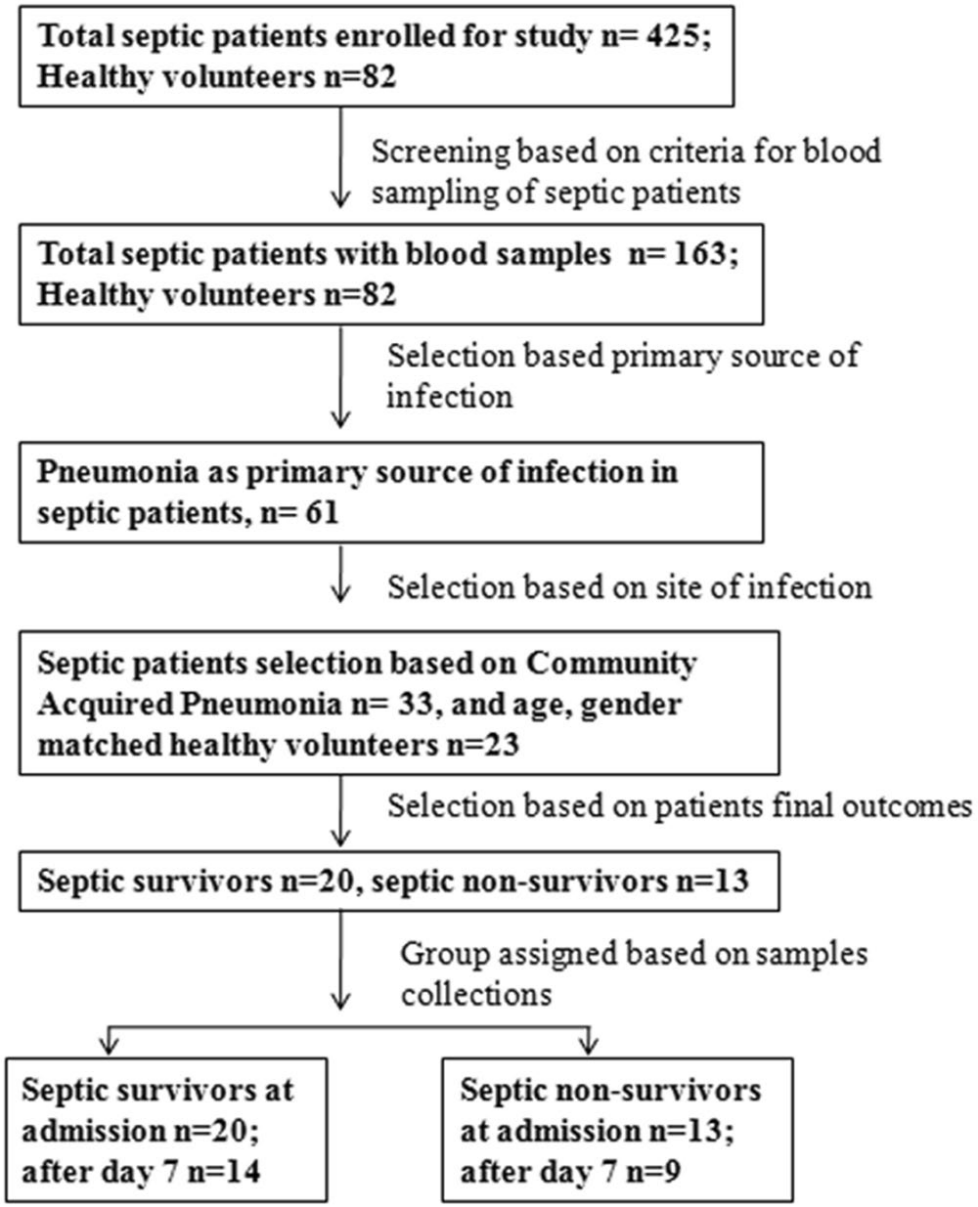
Schematic representation of a flow chart of a septic patient’s enrollment and selection Patients admitted to intensive care units with severe sepsis and/or septic shock were selected based on criteria of blood sampling, source and site of infection and their assigned group according to the final patient outcomes (survivors and non-survivors).

A total of 425 septic patients were prospectively enrolled in the cohort, and 164 patients met the criteria for blood sampling. Of these 164 patients, 33 had CAP as the primary source of infection^22^ and were selected for this study, 20 of whom survived and 13 of whom died during hospitalization (Fig. 1).

### Proteome changes

**Figure 2:**
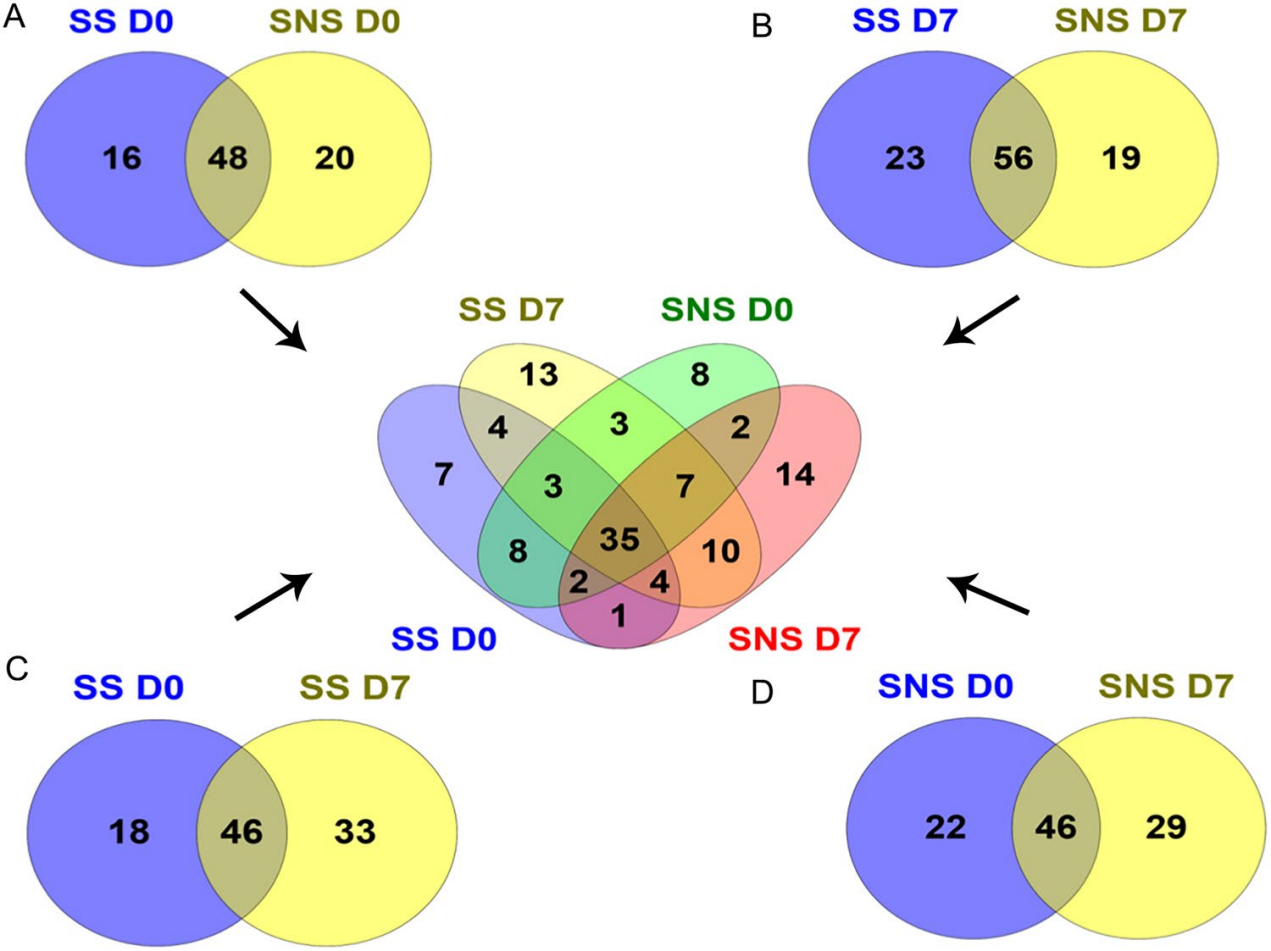
Venn diagram showing differential proteome expression between septic patient groups. The differential expression of proteins in survivors and non-survivors at admission (**A**) and D7 (**B**); the differential expression at D0 and D7 in survivors (**C**) and in non-survivors (**D**). The Venn diagram was created using the online tool Venny 2.1. http://bioinfogp.cnb.csic.es/tools/venny/index.html).

We have identified a total of 234 proteins with 1% FDR using the Swiss-prot database. After removing albumin and immunoglobulins and their isoforms from the identified proteins, we selected 179 proteins for further analysis. Compared with healthy volunteers, we identified 64 differentially expressed proteins in survivors at admission (D0) and 79 proteins in follow-up samples (D7). Similarly, we identified 68 and 75 differentially expressed proteins in non-survivors at D0 and at D7, respectively (Supplementary Table S1). There were 48 proteins commonly differentially expressed, 16 proteins were specific to survivors, and 20 proteins were specific to non-survivors at day 0 (Fig. 2A). Similarly, 56 proteins were commonly differentially expressed, 23 proteins were specific to survivors, and 19 proteins were specific to non-survivors after D7 (Fig. 2B). When protein changes were compared between D0 and D7 according to disease progression, 46 proteins were common in survivors at D0 and D7, while 18 and 33 proteins were specific to D0 or D7, respectively (Fig. 2C). Similarly, 46 proteins were found in non-survivors at D0 and D7, while 22 and 29 proteins were specific to D0 or D7, respectively (Fig. 2D).

#### Gene ontology annotations

**Figure 3:**
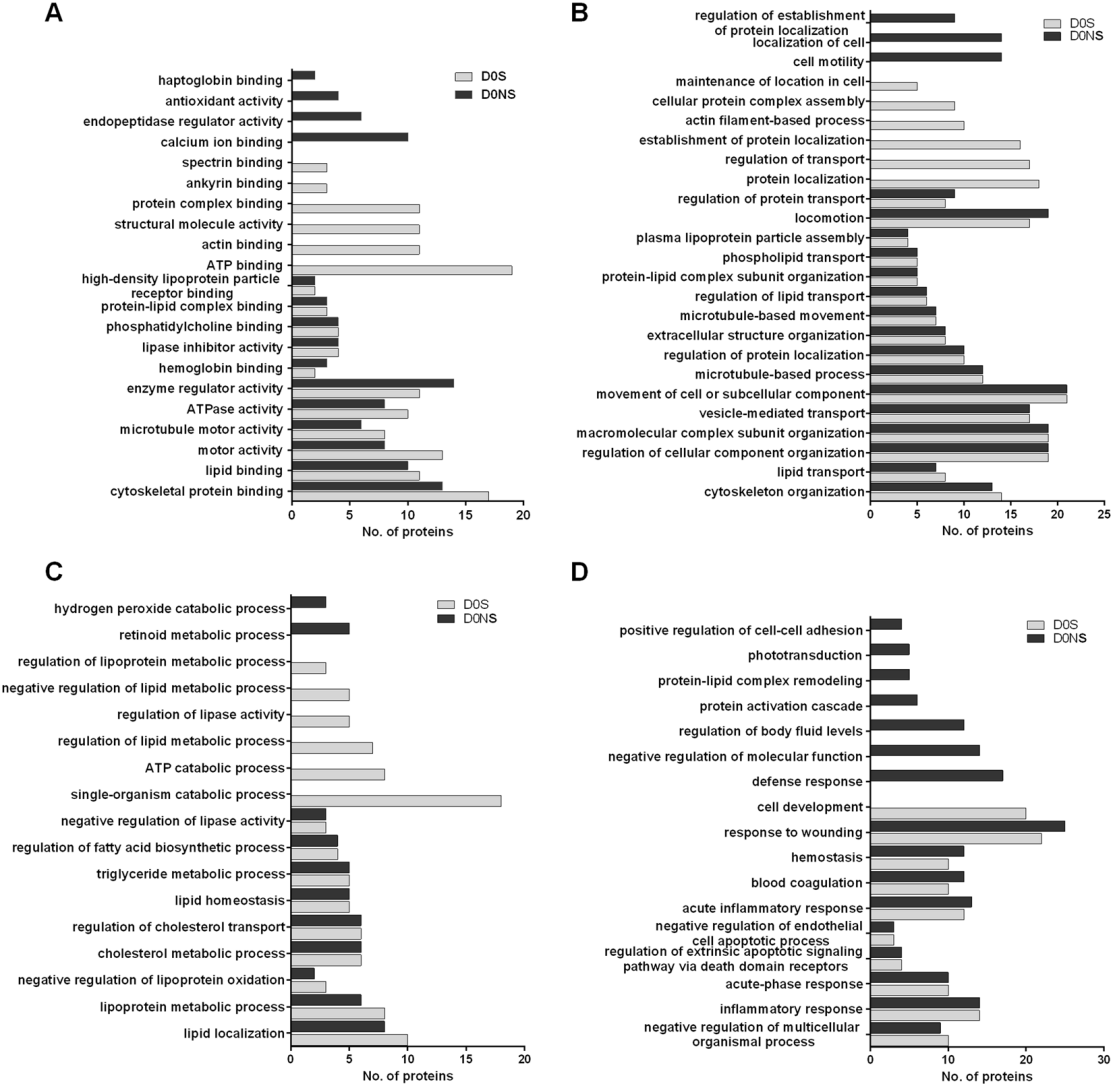
Gene ontology annotation for molecular function and biological processes for differentially expressed proteins. Altered molecular functions between septic survivors and non-survivors at admission are shown in (**A**). Biological processes were further categorized accordingly as follows: associated cellular organization and movement (**B**); alteration associated with metabolism (**C**); and immune response-related altered processes (**D**). The molecular functions and biological processes are shown at the corresponding P-value ≤ 0.01 with FDR B&Y multiple corrections.

Gene ontology annotation analysis enabled us to identify altered molecular function and biological processes (Supplementary Table S2). The top altered molecular functions that were identified in survivor and non-survivor groups at admission included cytoskeleton binding protein, motor activity, hemoglobin binding, lipid binding and microtubule motor activity (Fig. 3A). There are several altered biological processes listed that are further categorized into three major subtypes. In the first category, we included cellular assembly and transport-related processes, where cytoskeleton organization, macromolecular complex subunit organization, extracellular structure organization, microtubule-based movement, and protein/lipid/vesicle-mediated transport were observed in both groups. We found that maintenance of localization in the cell, cellular protein complex assembly, actin filament-based processes and regulation of transport were unique to survivors. Cell motility, localization of the cell and establishment of protein localization were found in non-survivors (Fig. 3B). In the second category, metabolism-related processes were included where lipoprotein/cholesterol/tryglyceride metabolic processes and the regulation of the fatty acid biosynthetic process were observed in both groups. The regulation of the lipoprotein/lipid metabolic process, single organism catabolic process and ATP catabolic process were observed in survivors, and hydrogen peroxide/retinoid catabolic processes were found in non-survivors (Fig. 3C). In the third category, we focused on immune response-related processes, such as homeostasis, blood coagulation, response to wounding, inflammatory response and acute-phase response, which were observed in both groups. Other processes, such as the defense response, regulation of body fluid levels and protein activation cascade, were altered in non-survivors (Fig. 3D).

At D7, we found more altered processes in the immune response, while other categories showed different responses between survivors and non-survivors (Supplementary Fig. S1). ATP binding, actin binding, spectrin binding, ankyrin binding and protein complex binding were observed in survivors, and haptoglobin binding, antioxidant activity, calcium ion binding and endopeptidase regulator activity were observed in non-survivors after 7 days.

#### Functional analysis with ingenuity pathway analysis

Several biological processes were identified and categorized on the basis of their IPA score. Metabolic disease, lipid metabolism, small molecule biochemistry and cellular assembly and organization were identified with the highest IPA scores in all groups; however, specific molecules have different expression values in different groups (Supplementary Table S3). There are several key molecules that hold the central node, such as CRP, LBP and HDL, and interact with several other differentially expressed proteins, such as the family of apolipoproteins (S) (Supplementary Fig. S2).

**Figure 4:**
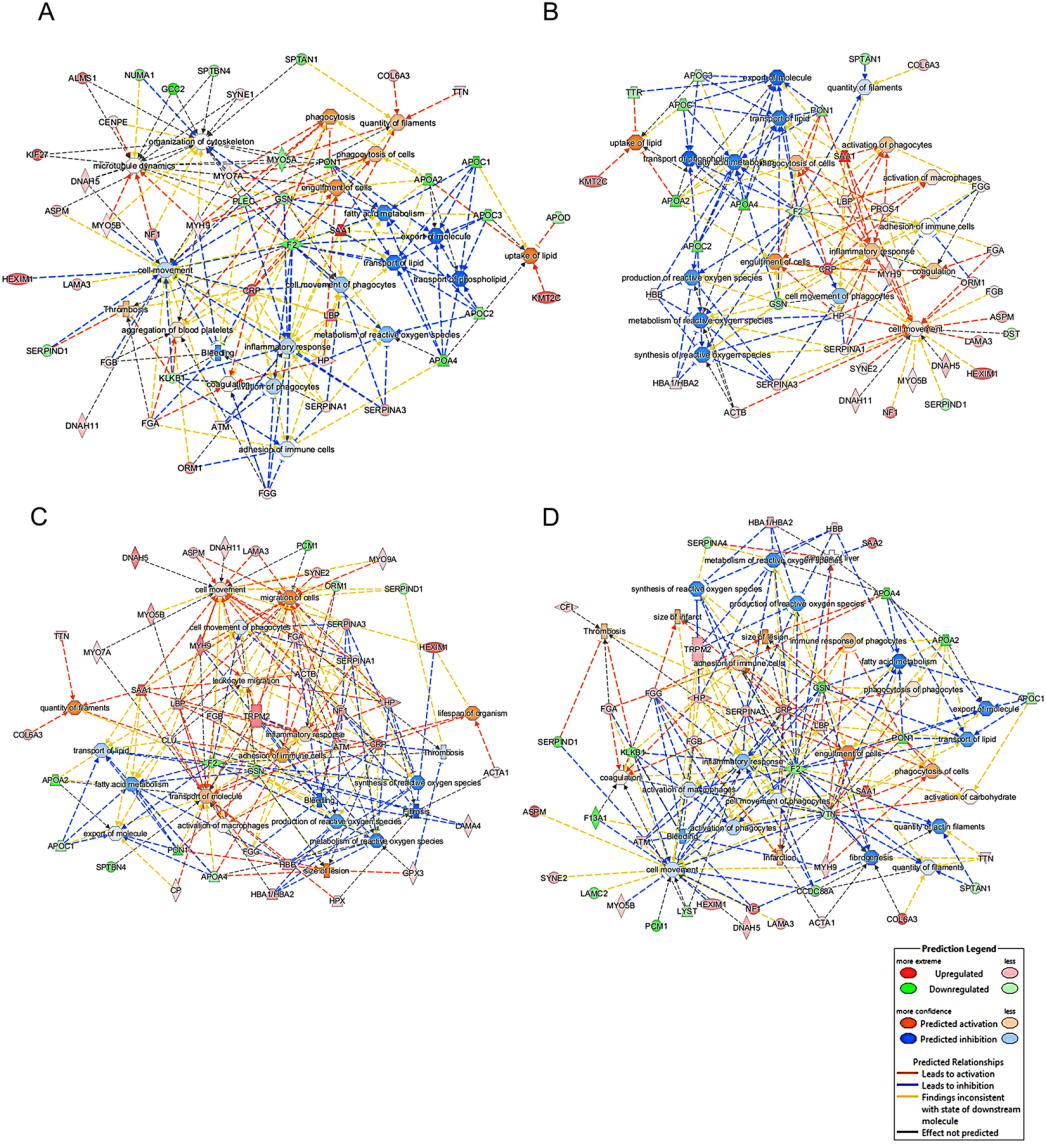
Functional analysis curated by Ingenuity Pathway Analyses. Interaction between altered functions and their association with proteins are represented based on their Z-score in the functional network. (**A)** Refers to the functional alteration in survivors; (**B)** Refers to non-survivors at admission; (**C**) Refers to D7 survivors; and (**D**) refers to D7 non-survivors.

Functional analysis demonstrated activation or inhibition of functions based on z-scores (Supplementary Table S4). We found that the uptake of lipids, engulfment of cells, coagulation and phagocytosis of cells were activated; however, fatty acid metabolism, export of molecules, transport of lipid and phospholipids, cell movement of phagocytes and metabolism of reactive oxygen species (ROS) were inhibited in survivors and non-survivors after admission. We also found that some functions were outcome-specific. Microtubule dynamics, organization of the cytoskeleton, phagocytosis and quantity of filaments were activated, and bleeding and adhesion of immune cells were inhibited in survivors (Fig. 4A). Activation of macrophages and synthesis and production of ROS were inhibited in non-survivors (Fig. 4B). Interestingly, we identified cell movement and inflammatory response inhibition in survivors, while activation was observed in non-survivors; this pattern was inversed at D7. In D7 samples, migration of cells, transport of molecules and adhesion of immune cells were activated, and synthesis, production and metabolism of ROS, bleeding, transport of lipid and export of molecules, and thrombosis were inhibited in survivors (Fig. 4C). Similarly, engulfment of cells, thrombosis, infarction and phagocytosis were activated, and the quantity of actin filaments, activation of macrophages and phagocytes and cell movement of phagocytes were inhibited in non-survivors (Fig. 4D).

#### Canonical pathway analysis

**Figure 5:**
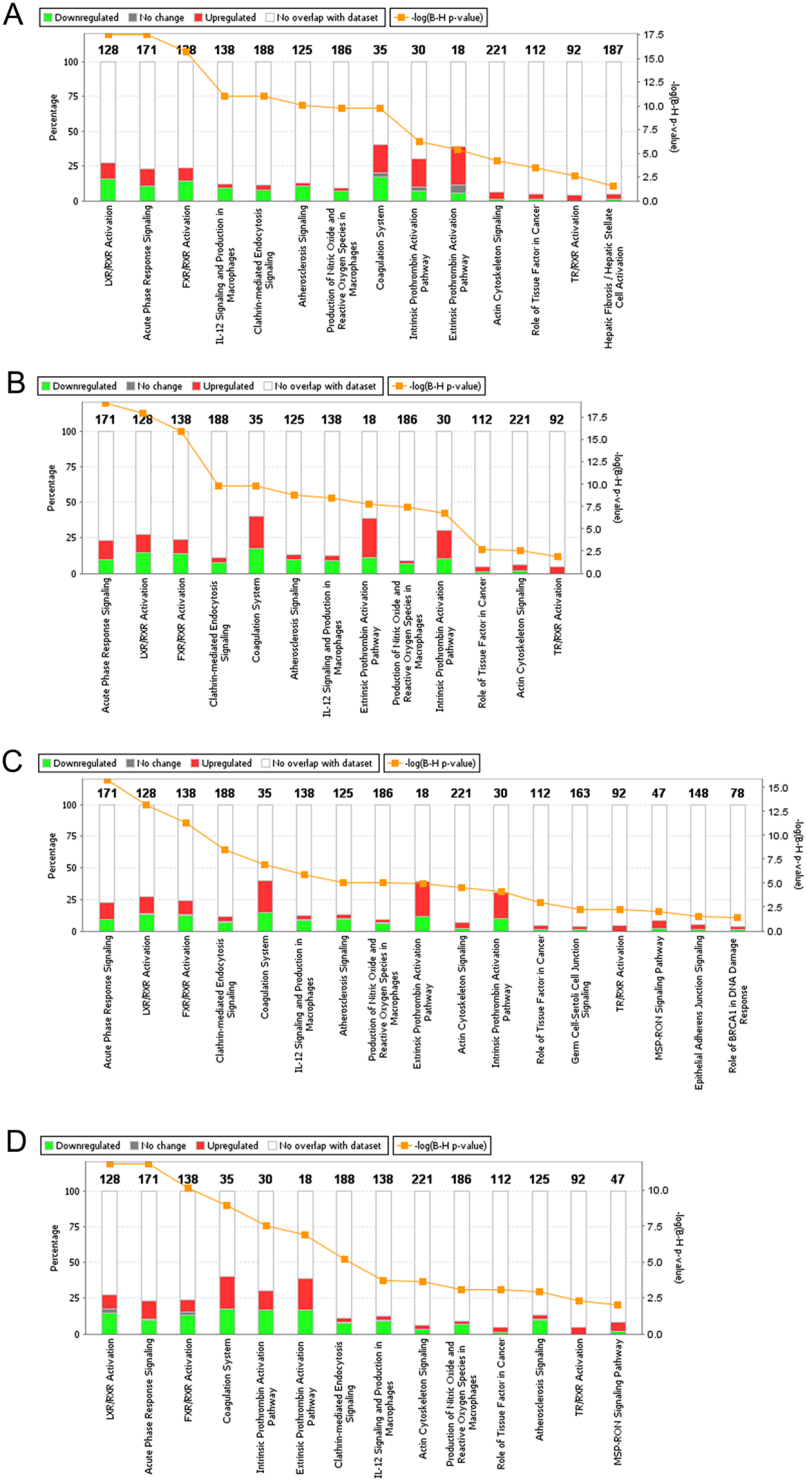
IPA canonical pathway analysis in septic patients. (**A**) Refers to altered canonical pathway in survivors; (**B**) refers to non-survivors at admission; (**C**) refers to survivors; and (**D**) refers to non-survivors after D7. Enriched canonical pathways were identified from the IPA library using Fisher’s exact test adjusted for multiple hypothesis testing using the Benjamini- Hochberg correction.

Canonical pathway analysis identified altered pathways and top-listed pathways with significant P-values and included LXR/RXR activation, acute-phase activation, FXR/RXR activation, interleukin signaling, coagulation, intrinsic and extrinsic prothrombin activation pathway and actin cytoskeleton signaling in both groups; however, the number of proteins, P-values and expression values were different in survivors and non-survivors (Fig. 5 and Supplementary Table S5).

### Proteins related to altered biological functions

#### Proteins related to organization of the cytoskeleton and microtubule dynamics

We have identified several differentially expressed proteins that are responsible for different functions in the living system. Several proteins representing cellular assembly were found to be altered in all groups, such as KIF27, NF1, MYH9, MYO5B, ALMS1, SYNE1, ASPN (upregulated) and GSN, PLEC, PON1, F2, GCC2 (downregulated). Some proteins of a dynein heavy chain family member, a microtubule-dependent motor ATPase, have been identified after admission, including DNAH5, DNAH8, DNAH10, DNAH11 and DNAH12 (upregulated); DNAH5, DNAH10, and DNAH11 were identified in survivor follow-up groups. We identified some proteins in survivors at D0 and D7, such as OBSCN, TTN (upregulated) and SPTBN4 (downregulated), while SYNE2 (downregulated) was identified in non-survivors at D0 and D7. NUMA1 and SPTAN1 (downregulated) were identified after admission and in the survivors group after day 7. Importantly, we also identified changes in several proteins such as ACTA1, ACTB, MYH13, MYO5A, MY07A and MYO9A in the different groups (Supplementary Table S1).

#### Proteins related to cell movement

Cell migration is a central process in the immune response or the pathogen entry; impairment of this function has serious consequences. Apart from some proteins included in the cytoskeleton, we identified several other proteins, including HEXIM1, LAMA3, FGA, FGB, ATM, DNAH11, SERPINEA3, SAA1, HP, CRP, LBP (upregulated) and SERPINAD1 and KLKB1 (downregulated). ORM1 protein was upregulated after admission and downregulated in follow-up samples. Some of these proteins, such as SAA1, HP, CRP and LBP, were included in the acute-phase proteins (Supplementary Table S1).

#### Proteins related to energy metabolism

Energy metabolism is the process of generating ATP and is essential in maintaining homeostasis and various signaling in the cell. Dysregulation in energy metabolism leads to the alteration of various cellular functions and signaling. We identified downregulated apolipoprotein family proteins, such as APOA1, APOA2, APOA4, APOB, APOC1, APOC2, APOC3, and APOE in septic patients. Apolipoprotein members APOD and APOE were downregulated after admission. Some of these proteins are responsible for the uptake of lipids and the transport of phospholipids. Apolipoproteins with PON1, GSN, SAA1, LBP, MYO5A, and F2 are responsible for alterations in fatty acid metabolism, and with the addition of SERPINA1 and SERPINA3, altered reactive oxygen species metabolism (Supplementary Table S1).

#### Proteins related to inflammation, coagulation and bleeding

Inflammation is a complex biological response against pathogens. In the present study, we identified several inflammatory response-related proteins, including HP, FGG, ATM, SERPINA1, SERPINA3, CRP and LBP (upregulated) and F2, GSN and PON1 (downregulated). Some of these proteins are also key molecules in coagulation and bleeding.

### Validation results

#### Confocal analysis for actin and gelsolin

**Figure 6:**
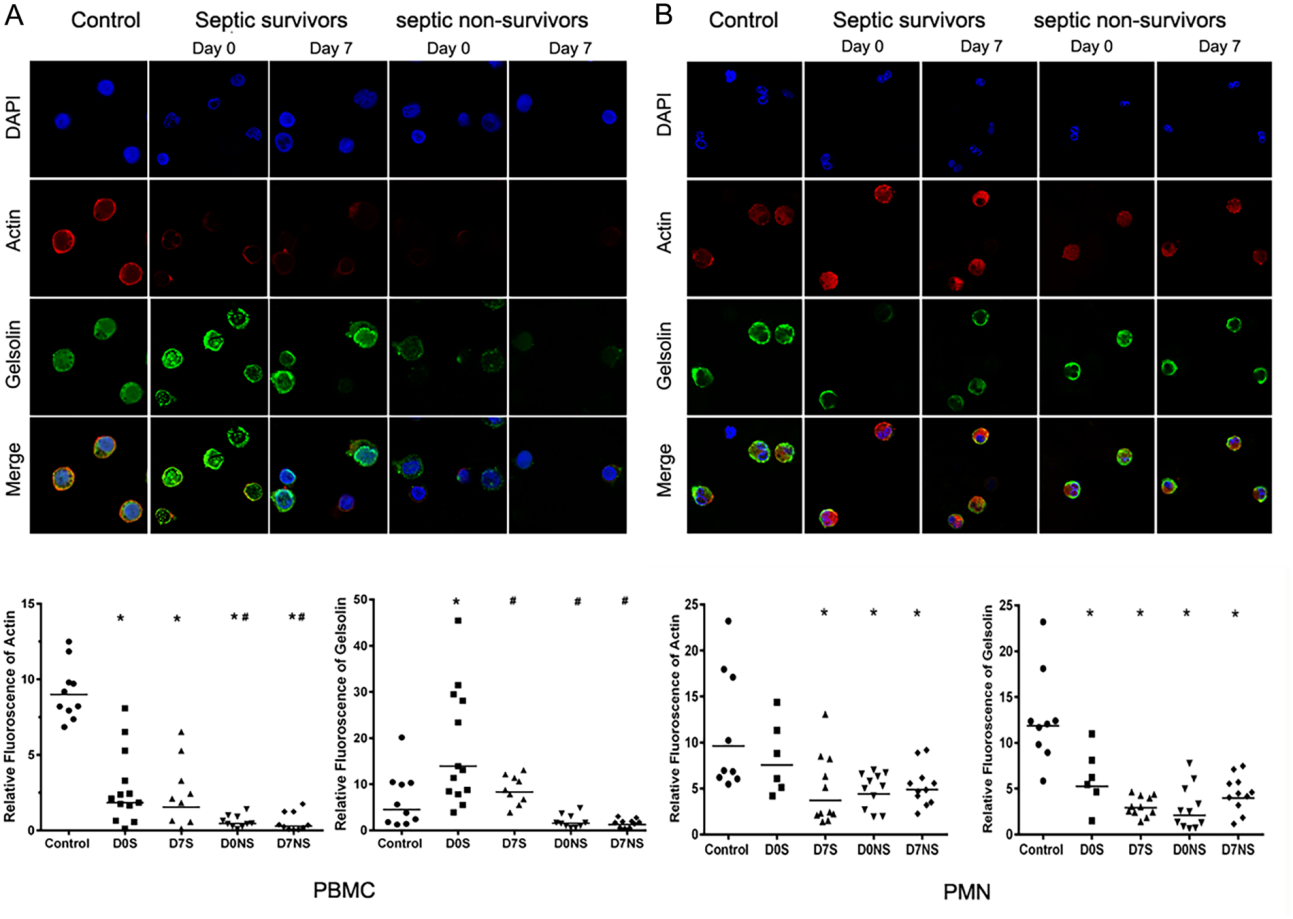
Confocal microscopy for actin and gelsolin in circulating blood cells. (**A)** Refers to cellular expression for actin and gelsolin with nuclear staining in peripheral blood mononuclear cells (PBMCs) of healthy volunteers (n = 10), septic survivors (n = 13 at D0 and n = 9 at D7) and non- survivors (n = 10, at admission and after D7). Similarly, (**B)** refers to cellular expression for actin and gelsolin in polymorphonuclear cells (PMNs) in healthy volunteers (n = 9), septic survivors (n = 6 at D0 and n = 11 at D7) and non-survivors (n = 11 at D0 and at D7). Data are represented as the geographic means of cell fluorescence analyzed using ImageJ software (National Institutes of Health, Bethesda, MD, USA) for actin and gelsolin. *Denotes P ≤ 0.05 when compared to control, ^#^Denotes when compared to septic survival at admission.

Proteomic data have provided evidence for a higher expression of actin (main component of the cytoskeleton) in plasma. To confirm the proteomic data, we compared actin in the PBMCs and PMNs from septic patients to that of healthy volunteers. We found a decreased expression of actin in the septic patient’s blood mononuclear cells, with acute depletion in the non-survivors. In contrast, gelsolin had a higher expression in survivor septic patients than in non-survivor septic patients (Fig. 6A). Changes in the PMNs showed a different pattern; gelsolin was decreased in septic patients, and actin was decreased only in the non-survivor groups (Fig. 6B).

#### Lipid and lipoprotein analyses

**Figure 7:**
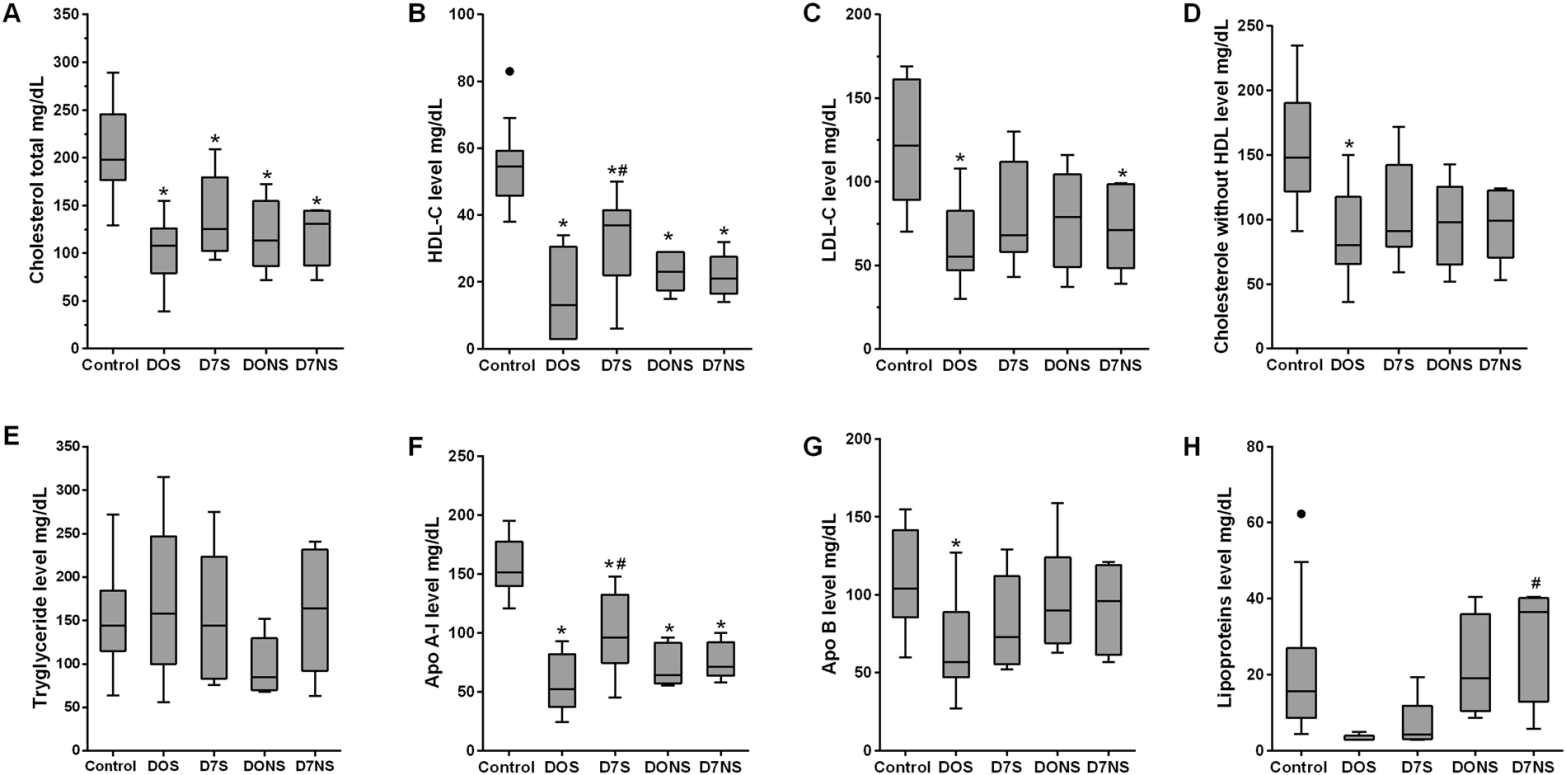
Estimation of lipid moieties in blood plasma. (**A**) Refers to total cholesterol; (**B**) refers to HDL-C; (**C)** refers to LDL-C; (**D**) refers to HDL free cholesterol; (**E**) refers to triglyceride levels; F refers to apo A-I; (**G**) refers to Apo B; and (**H**) refers to lipoprotein fraction levels in healthy volunteers, septic survivors and non-survivors. The results are presented as box plots and analyzed using one-way ANOVA with Tukey’s post hoc multiple comparison test, where P ≤ 0.05 is considered significant. *Denotes P ≤ 0.05 when compared to control, ^#^Denotes when compared to septic survival at D0.

Our proteomic data showed alterations in lipid metabolism and dysregulation of apolipoproteins. For further confirmation, we estimated the level of total cholesterol, HDL-C, LDL-C, triglycerides, ApoA-I, Apo B and lipoproteins. We found that the level of total cholesterol was significantly decreased in septic patients, and no significant differences were observed between survivor and non-survivor septic patients (Fig. 7A). The HDL-C levels decreased significantly in septic patients, and increased levels have been observed in survivors at D7 compared to D0. In contrast, no significant differences were observed in non-survivor septic patients in the follow-up samples (Fig. 7B). LDL-C and HDL free cholesterol levels were also decreased in septic patients, whereas survivors showed significant changes at admission (Fig. 7C and D). No significant differences were observed in triglyceride levels in septic patients (Fig. 7E).

Apo A-I is the major protein of HDL composition. We found significantly decreased levels in septic patients in a similar pattern as HDL-C (Fig. 7F). Apo B protein was significantly decreased only in survivors at D0, while no significant differences were observed in lipoprotein levels (Fig. 7G and H).

## Discussion

Sepsis is a syndrome resulting from various sources of infection that lead to changes in the inflammatory response and cellular metabolism, ultimately resulting in organ dysfunction and death^23^. We aimed to evaluate these changes at the protein level and selected those patients with CAP to avoid the heterogeneity of sepsis secondary to multiple sources of infection, the impact of underlying diseases and previous interventions of patients with hospital-acquired infections.

Overall, the number of proteins identified was similar in the different groups of septic patients, and specific changes were observed for survivors and non-survivors as well as for admission and follow-up samples. Gene ontology results pointed to major changes in the cytoskeleton and cell movement, energy metabolism and immune response-related processes. These changes were further supported by IPA analyses showing that the top altered functions included cellular assembly and organization, the actin-cytoskeleton pathway, metabolic disease, lipid metabolism and acute-phase response signaling.

Our current findings corroborated with the meta-analysis of transcriptomic data showing the cytoskeleton as a top altered pathway in septic patients^24^. We found several proteins related to the organization of the cytoskeleton and microtubule dynamics that were altered in our patients. Importantly, actin, gelsolin and myosin, major components of the cytoskeleton, were altered in septic patients; actin levels were increased, and levels of gelsolin were decreased in plasma. Interestingly, immunofluorescence analyses showed a decreased expression of actin in mononuclear cells of septic patients that was more pronounced in non-survivors than in survivors. Actin was also decreased in polymorphonuclear cells (PMNs), with a greater magnitude in non-survivors than in survivors. Gelsolin presented with a different pattern in mononuclear cells and PMNs, with increased expression in mononuclear cells in survivors and decreased expression in PMNs in the entire cohort of septic patients.

Actin is a conserved essential multifunctional protein and structural unit of the cytoskeleton of the cell. Dysregulation of actin resulted in the alteration of various cellular processes, transport of molecules, signaling pathways, cell migration and wound repair. We found an altered actin cytoskeleton pathway, which supported previous proteomic results in septic patients^21^. Lee *et al*. showed circulating actin in the plasma of septic patients^25^, which increased blood viscosity, obstructing small blood vessels^26,27^ and enhancing the bacterial infections under activation of purinergic receptors through binding to adenine nucleotides and the production of alpha hemolysine^28^. The dead cells supposedly are the source of free actin in plasma; however, our results suggested that circulating blood cells can also be a source of free actin.

Gelsolin is an actin-binding cytoplasmic and circulating protein, which effectively scavenges actin filaments in blood plasma^27^. It also binds to pro-inflammatory and bioactive mediators to localize inflammation, bacterial surface lipopolysaccharides, and inactivates bioactive lipid mediators such as lysophosphatidic acid, Gram-negative bacteria lipopolysaccharide, and platelet-activating factor^29,30^. Plasma gelsolin levels are significantly decreased in major trauma, burns, surgery and sepsis, whereas recovery of circulating gelsolin has been correlated with improvement in patient outcomes. Recombinant gelsolin administration showed a reduction in mortality in experimental sepsis^31–33^. Thus, our proteomic results showing increased levels of actin and decreased levels of gelsolin in plasma are consistent with previous reports and stress important changes in the cytoskeleton during sepsis. A higher expression of gelsolin and lower expression of actin were detected in mononuclear cells from survivor patients, which suggests that gelsolin nucleates actin monomers in filaments for maintaining the actin cytoskeleton. In contrast, a lower expression of actin and gelsolin found in septic non-survivors may make mononuclear cells more vulnerable, hence resulting in alterations in cellular assembly and maintenance. Recently, the ratio of actin to gelsolin in plasma was suggested as a biomarker for sepsis^34^.

Our proteomic results showed downregulation of apolipoproteins, such as ApoA2, ApoA4, ApoC1, ApoC2, ApoC3, Apod and Pon1, and upregulation of acute-phase proteins, such as SAA, HP, HPR and LBP, in the plasma of septic patients. Hence, lipids and lipoproteins were further evaluated in plasma to support our proteomic findings. Accordingly, we found lower levels of total cholesterol, HDL-C and APO A-I in the plasma of septic patients compared to healthy volunteers using enzymatic, colorimetric and immunoturbidimetric methods.

Septic patients have been reported to present low levels of total cholesterol^35^ and high-density lipoprotein (HDL)^36^. de la Llera *et al*. have described the alteration of HDL composition and function with increased expression of SAA, decreased expression of phospholipids, apoA-I, lipid-poor pre-β1 and small-medium-sized particles in response to human endotoxemia^37^. Recently, Cirstea *et al*. demonstrated decreased HDL-C levels as a prognostic marker for early organ failure and death in septic patients. The study suggested that the decreased levels of HDL-C are unchanged for 7–10 days in hospitalized septic patients^38^. Interestingly, our current finding demonstrated a similar pattern in non-survivors, while septic survivors showed an increased expression in follow-up samples compared to samples at admission.

Apolipoproteins transport cholesterol to other cells and tissues; hence, downregulation resulted in an alteration of cholesterol transport. Guo *et al*. demonstrated decreased LPS neutralization in apoAI knockout mice lacking HDL^39^. Low levels of HDL-C and apoA-1 in severe septic patient plasma is associated with increased mortality^40^. In contrast, higher levels of apoA-I improve survival^41^ and reduce TNF-α levels in response to LPS in rodents^42^. These studies support a role for apoA-1 in inhibiting the inflammatory cytokine production in sepsis^35^. Septic patients showed displacements of apoA-I with SAA; hence, SAA became the major component of HDL^36,43^. Our results showed increased SAA and decreased Apo A, which further supports these changes in the HDL components.

Changes in lipid moieties revealed the inhibition of LXR/RXR activation as the most altered pathway using IPA in our cohort of septic patients. These results corroborate with the results from a previous study in CAP patients, which also found LXR/RXR as the most altered pathway^21^. This pathway is involved in the regulation of lipid metabolism, inflammation, and cholesterol-to-bile acid catabolism; hence, it resulted in the alteration of lipid metabolism in patients.

Our proteomic data suggested counter-regulatory action to the excess of production, synthesis and metabolism of ROS in septic patients through increased expression of acute-phase response proteins. We have previously shown that monocytes and neutrophils from septic patients have an increased capacity to generate NO and ROS^44,45^. Mitochondria are a source and target of ROS in sepsis. In our previous transcriptomics study involving septic patients with CAP that belong to this same cohort, we observed differences in the expression profile of genes related to the mitochondrial electron transport chain (ETC) I-V between survivors and non-survivors at the time of patient admission^46^. Furthermore, using a PCR-array encompassing genes belonging to the interacting TLR cascades, NADPH-oxidase and mitochondrial oxidative phosphorylation, we found that the redox imbalance (iNOS signaling, oxidative phosphorylation and superoxide radical degradation) affecting mitochondrial functions was prominent in non-surviving septic patients^47^. The present proteomic data also suggested the activation of phagocytosis, engulfment and phagocytosis of cells in septic patients. This is in contrast with the current concept showing hyporesponsiveness of innate immune cells in sepsis^48^; however, it is in agreement with experimental data showing that bone marrow-derived mice macrophages and human monocytes made tolerant to TLR agonists presented with increased phagocytosis and respiratory burst^49,50^. Further support is provided by our previous results showing that monocytes of septic patients presenting with a decreased production of inflammatory cytokines showed an increased level of RONS and preserved phagocytosis^51^.

Finally, we found inflammatory and immune response-related processes as well as activation of coagulation and the intrinsic and extrinsic prothrombin activation pathway in sepsis, supporting data from transcriptomics and proteomic studies^13,52,53^.

Previous proteomic studies revealed a wide range of altered pathways in sepsis. Our data add important new insights to the previously described studies and confirmed some previous findings. The cytoskeleton alteration, previously demonstrated by the detection of few circulating proteins such as actin and gelsolin, could be documented by a number of proteins involved in cytoskeleton assembly and cellular organization. As a result, this pathway emerged as one of the most altered in gene ontology and functional analyses. Furthermore, our results showed metabolic disease, lipid metabolism and small molecule biochemistry as the top altered functions in septic patients and identified HDL and lipid moieties as important components in the interaction network. Thus, our proteomic results stressed the important changes in cellular structure and metabolism, which could be possible targets for future interventions of sepsis.

Our study has some strengths and limitations. Patients were selected from a multicenter sepsis cohort, and heterogeneity was partially circumvented by selecting patients with a single source of community-acquired infection. The number of proteins identified in our study is in the range of those reported in the literature, despite a few studies that identified more proteins than our study. Moreover, selecting septic patients with CAP restricted the number of patients with preserved plasma samples. Despite validating proteomic results using an immunofluorescence cellular assay and the plasma determination of lipid moieties, several cell functions showing alterations at the protein level were not evaluated in *in vitro* experiments. Nevertheless, most of the altered pathways were consistent with previous genomic and proteomic results as well as with described cellular changes in septic patients.

## Methods

### Patients, sample collection and processing

A cohort of septic patients was enrolled from the Intensive Care Units of three general hospitals located in São Paulo, Brazil. This study followed the guidelines of the Declaration of Helsinki for research on human participants and was approved by the ethics committees of the participating hospitals, São Paulo Hospital (Study number 1477/06), Albert Einstein Hospital (Study number 07/549) and Sírio-Libanês Hospital (Study number 2006/27). Informed consent was obtained from all participants or relatives before enrollment. Patients were enrolled within 48 hours of the first occurrence of organ dysfunction indicative of severe sepsis or septic shock. Exclusion criteria included patients receiving immunosuppressive therapy, and patients diagnosed with AIDS, end-stage chronic illness, or who had been submitted to experimental therapy. Patients with CAP as the primary source of infection, older than 40 years of age, were selected for the study. Twenty-three healthy volunteers matched for age- and gender were included as the control group (Table 1).

Blood samples from septic patients were collected at admission (D0) and after seven days (D7). Peripheral blood mononuclear cells (PBMC) and polymorphonuclear cells (PMN) were obtained using the Ficoll-Paque PLUS (GE Healthcare Bio-Sciences AB, Uppsala, Sweden) and Dextran 3% (Amresco, Solon, Ohio, USA), respectively. Cells were stored in liquid nitrogen until use.

### Depletion of plasma

Highly abundant proteins such as albumin and immunoglobulin were depleted using a proteome minor kit (BioRad, California, USA).

### Digestion

After determining the protein concentration using the Bradford assay, aliquots of up to 100 μg of protein were placed into separate tubes, and the sample volumes were equalized using 1 M TEAB. Cysteine disulfide bonds were reduced with 0.1 volumes of 50 mM TCEP, and samples were mixed on a vortex, quick spun and incubated at 60 °C for 1 h. Reduced cysteine residues were alkylated by adding 0.05 volumes of 200 mM methylmethanethiosulfate. Samples were mixed on a vortex, quick spun and incubated at room temperature (RT) for 10 min. To each tube, 10 μg of trypsin was added, which had been reconstituted in the supplied buffer and diluted in 20 μl of 1 M TEAB, mixed on a vortex, quick spun and incubated at 37 °C overnight.

### iTRAQ labeling

The sample volume was reduced using a SpeedVac at RT, and the volume was adjusted back to 30 μl using 1 M TEAB. The required iTRAQ reagents were removed from the freezer, and the reagent vials were quick spun. A total of 60 μl of isopropanol was added to each reagent vial, mixed and quick spun. The entire contents of the reagent were transferred to appropriate sample tubes, well-mixed and incubated at RT for 2 h. The volume was reduced to ~30 μl on a SpeedVac at RT to prevent the sample from drying out.

### Sample fractionation using SCX chromatography

A total of 100 μl of SCX-A buffer was added to each sample tube, and all tubes were pooled in a single vial. The pH was adjusted to <2.7 using 1 M hydrochloric acid. The entire sample was transferred to a glass sample vial, and the volume was adjusted to 1.6 ml with SCX-A. After this, the sample was loaded onto the analytical column using a 2-ml injection loop and was washed with 100% SCX-A at the rate of one ml per minute for 30 min. The peptide mixtures were separated using a gradient that increased from 0 to 15% SCX-B over 35 min, 15 to 30% SCX-B in 5 min and 30 to 100% SCX-B in 5 min at a flow rate of 400 μl per minute. Fractions of peptides were collected into 1.5-ml tubes after each minute. Typically, 40 fractions were collected from 5 min to 45 min after the start of the gradient. Peptide fractions were dried to ~20 μl. After the separation of peptides, 100% SCX-B was run for 15 min to wash the column and re-equilibrated at 100% SCX-A for a final 15 min.

### LC-MS/MS analysis

LC-MS/MS analysis of iTRAQ-labeled peptides was performed on a Synapt G2 mass spectrometer interfaced with a nanoacquity UPLC nanoflow liquid chromatography system (Waters, Miliford, MA, USA). The fractions were enriched on a trap column (180 µm × 2 cm, 5 µm, Waters, Miliford, MA, USA) at a flow rate of 8 µl/min for 5 min and then resolved on an analytical column (75 µm × 15 cm, 1.7 µm, Waters, Miliford, MA, USA) with the application of a voltage of 3Kv. The peptides were eluted using a linear gradient of 7–30% solvent B (90% acetonitrile in 0.1% formic acid). LC-MS/MS data were acquired using positive ion mode in a data-dependent manner from m/z 300 to 1600Da, targeting the three most abundant ions in the survey scan. MS data were acquired in the QTOF analyzer, and MS/MS spectra were acquired for 1.5 seconds. Collision-induced dissociation mode (CID) was used for MS/MS scans.

### Mass spectrometry data analysis

The individual raw files were processed with Mascot Distiller (Matrix Science, USA) and all files were merged using Mascot Daemon software. The merged processed data were searched against a reviewed UniProt database containing human proteins with known contaminants (20,120 entries). The parameters used for data analysis including trypsin as a protease (allowed one missed cleavage), iTRAQ label at N-terminus and lysine residues, and cysteine modification by methyl methanethiosulfonate (MMTS) were specified as fixed modifications. Oxidation of methionine was specified as a variable modification. The precursor and product ion mass error tolerance were fixed at 20ppm and 0.1 Da, respectively. The peptide and protein data were extracted using high peptide confidence (P ≤ 0.05), and a minimum of 2 peptides were used for protein identification. The false discovery rate (FDR) was calculated using decoy database searches. Peptides identified at 1% FDR were used for protein identification. The results from Mascot Server were loaded in isobaricQ for iTRAQ quantitation.

### Bioinformatic analysis of proteomics data

To understand the functions and signaling that are affected during sepsis, we converted the protein list to gene names and analyzed them with ToppGene Suite and Ingenuity Pathway Analysis (IPA) (Qiagen bioinformatics, CA, USA). For gene ontology annotations, a differentially expressed gene list was uploaded in the section of ToppFun of ToppGene Suite with FDR B&Y correction with a P-value cut-off at 0.05 (https://toppgene.cchmc.org/enrichment.jsp). For IPA analysis, the gene list was uploaded in the IPA module and the fold change cut-off was set at ±1.3 for further functional, pathway and regulatory network analysis.

### Confocal microscopy

After thawed, PBMCs and PMNs were spun on glass slides (poly-lysine treated; 0.05%), fixed with 4.0% paraformaldehyde in PBS for 15 min at RT, washed with PBS, and then incubated with blocking solution PGS (0.2% gelatin, 0.1% saponin and 0.1% NaN_3_, diluted in PBS) for 1 h at RT. The cells were incubated overnight with the primary antibody, mouse anti-Gelsolin (1:100 diluted in PGS, Sigma-Aldrich, St. Louis, MO, USA). After washing with PBS, they were incubated with green fluorescent Alexa Fluor 488 anti-mouse (1:150), phalloidin-TRITC (1:1000 diluted in PGS, Sigma-Aldrich, St. Louis, MO, USA) for actin, and the nuclear material was stained with 4, 6-diamidino-2-phenylindole (DAPI; Sigma-Aldrich). Cells were then washed with PBS and mounted in glycerol buffered with 0.1 M Tris, pH 8.6, with 0.1% p-phenylenediamine as an anti-fade agent. Images of stained cells were acquired with a TCS SP5 II Tandem Scanner confocal microscope (Leica Microsystems, Wetzlar, Germany) using a 63x NA 1.44 PlanApo oil immersion objective and processed with Imaris® (Bitplane). The images were further analyzed in the program ImageJ (National Institutes of Health, Bethesda, Maryland, USA) using the measuring of cell fluorescence as described earlier^54^ and were quantified from the average correspondence of two to four cells/randomly selected field.

### Biochemical assays

To validate the proteomics data, we determined plasma levels of cholesterol and lipids moieties using biochemical assays (COBAS 6000 automated system, C501 Japan). All reagents were obtained from Roche (USA).

### Statistical analysis

For gene ontology, the probability density function was selected and the P-value cut-off was set at 0.01 using the FDR B&Y multiple correction method. Enriched canonical pathways were identified from the IPA library using Fisher’s exact test and adjusted for multiple hypothesis testing using the Benjamini-Hochberg correction. For functional activation and inhibition, Z-statistics were used where the value of Z ≥ 2.0 refers to predicted activation and Z ≥ −2.0 refers to predicted inhibition for particular functions. For the protein-protein functional interaction network, a 0.05-FDR threshold cut-off was set to identify genes corresponding to identified proteins whose expression was significantly differentially regulated. These genes, called focus genes, were overlaid onto a global molecular network developed from information contained in the database. Networks of these focus genes were then algorithmically generated based on their connectivity and assigned a score. Statistical analysis was performed using GraphPad Prism 6 (GraphPad Software, Inc., USA) for confocal and enzymatic colorimetric analyses. For confocal analyses, data were represented as the geometric means of cell fluorescence for actin and gelsolin and analyzed using one-way ANOVA followed by Tukeys’ multiple comparison test where P ≤ 0.05 was considered significant. For total cholesterol, HDL-C, LDL-C, HDL free cholesterol, triglycerides, Apo A-I, Apo B and lipoproteins, data were represented in box plots and analyzed using one-way ANOVA, and Tukey’s post hoc multiple comparison test was used where P ≤ 0.05 was considered significant Table 1.

**Table 1:** Clinical variables and demographic data from septic patients. All values are presented as the means ± SD. SOFA, Sequential Organ Dysfunction Assessment; COPD, Chronic Obstructive Pulmonary Disease; N/A, not applicable.

	Control (N = 23)	Sepsis (N = 33)	Survival (20)	Non-survival (13)
Age	64.95 ± 14.56	68.33 ± 15.84	63.75 ± 16.99	75.38 ± 11.12
Sex	60% male	66.6% male	60% male	76.92% male
Septic Shock	NA	81.10%	75%	92.30%
Apache II	NA	20.21 ± 5.95	20.01 ± 6.61	20.38 ± 5.0
SOFA	NA	8.12 ± 3.06	8.25 ± 3.5	7.92 ± 2.36
SOFA Delta (day3-day0)	NA	(−)0.5 ± 4.26	(−)2.5 ± 2.7	2.23 ± 4.54
Organ Dysfunction				
Cardiovascular	NA	87.80%	85.00%	92.30%
Renal	NA	45.40%	55%	30.76%
Respiration	NA	69.7	70%	69.23
Hematological	NA	18.18%	15%	23.07%
Hepatological	NA	15.15%	10%	23.07%
Nervous system	NA	42.42%	35%	53.84%
Underlying Diseases				
Corticosteroids	NA	42.42%	45%	38.46%
AIDS	NA	0	0	0
COPD	NA	27.27%	30%	23.07%
Immunosuppression	NA	9.09%	10%	7.69%

### Data availability

Most of data generated or analysed during this study are included in this published article (and its Supplementary Information files). Any further information is available from the corresponding author on reasonable request.

## Electronic supplementary material

Supplementary Information
