# Supplementary material for: Proteomic study revealed cellular assembly and lipid metabolism dysregulation in sepsis secondary to community-acquired pneumonia
Source: Sci Rep. 2017 Nov 15;7:15606. doi: 10.1038/s41598-017-15755-1 (PMC5688086; doi:10.1038/s41598-017-15755-1)
Supplement: Supplementary file 1 — Supplementary Information [file 41598_2017_15755_MOESM1_ESM.pdf]

Proteomic study revealed cellular assembly and lipid metabolism dysregulation in sepsis secondary to community-acquired pneumonia

Narendra Kumar Sharma[1], Alexandre Keiji Tashima[2], Milena Karina Colo Brunialti[1], Éden Ramalho Ferreira[3], Ricardo José Soares Torquato[2], Renato Arruda Mortara[3], Flavia Ribeiro Machado[4], Murillo Assunção[5], Otelo Rigato[6] and Reinaldo Salomao[1*]

[1]Division of Infectious Diseases, Escola Paulista de Medicina, Hospital São Paulo, Universidade Federal de Sao Paulo, Sao Paulo, Brazil
[2]Departamento de Biochemistry, Escola Paulista de Medicina, Universidade Federal de São Paulo, São Paulo, SP, Brazil
[3]Department of Microbiology, Immunology and Parasitology, Escola Paulista de Medicina, Universidade Federal de Sao Paulo, Sao Paulo, Brazil
[4]Intensive Care Unit, Hospital São Paulo, Escola Paulista de Medicina, Universidade Federal de Sao Paulo, Sao Paulo, Brazil
[5] Intensive Care Unit, Hospital Israelita Albert Einstein, Sao Paulo, Brazil
[6] Intensive Care Unit, Hospital Sirio Libanes, Sao Paulo, Brazil

Supplementary data

Fig. S1. Gene ontology annotation for molecular function and biological processes for differentially expressed proteins after D7.

Fig. S2. IPA generated protein-protein interaction network to identify altered functional categories in septic patients.

Table S1. Quantitative proteomics data with protein description, number of peptides matched, Mascot score and fold changes in septic patients.

Table S2. Detailed gene ontology annotations for septic patients for molecular function, biological processes and cellular components.

Table S3. IPA-generated functional interaction network based on scores for septic patients.

Table S4. Altered functions with their activation and inhibition z-scores in septic patients.

Table S5. IPA-identified canonical pathways in septic patients.

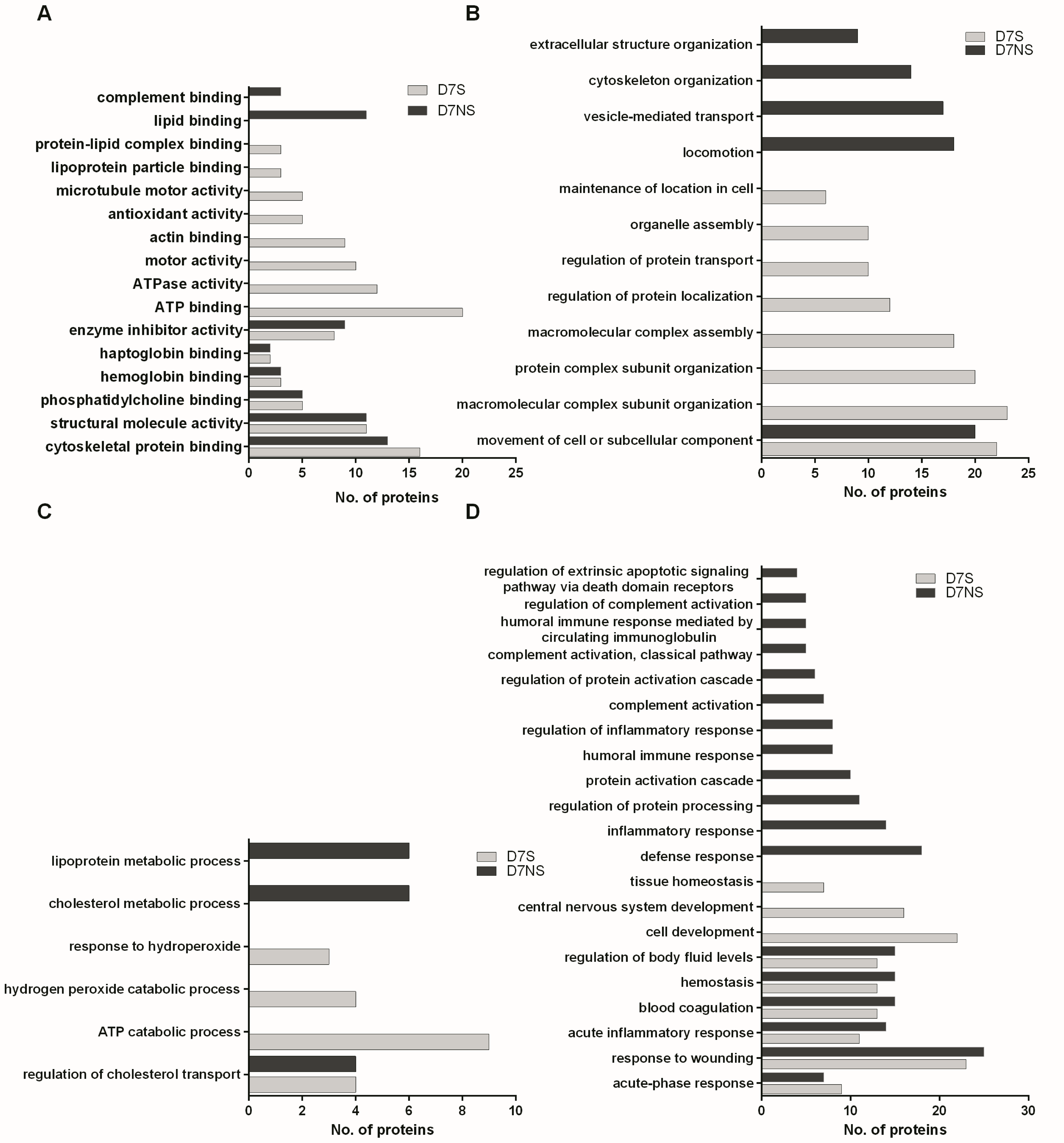

Fig. S1: Gene ontology annotation for molecular function and biological processes for differentially expressed proteins after D7. A refers to altered molecular functions between septic survivors and non-survivors. Biological processes were further categorized accordingly to the biological process: associated cellular organization and movement (B); alteration associated to metabolism (C) and to immune response related altered processes (D).

Fig. S2. IPA generated protein-protein interaction network to identified altered functional categories in septic patients.

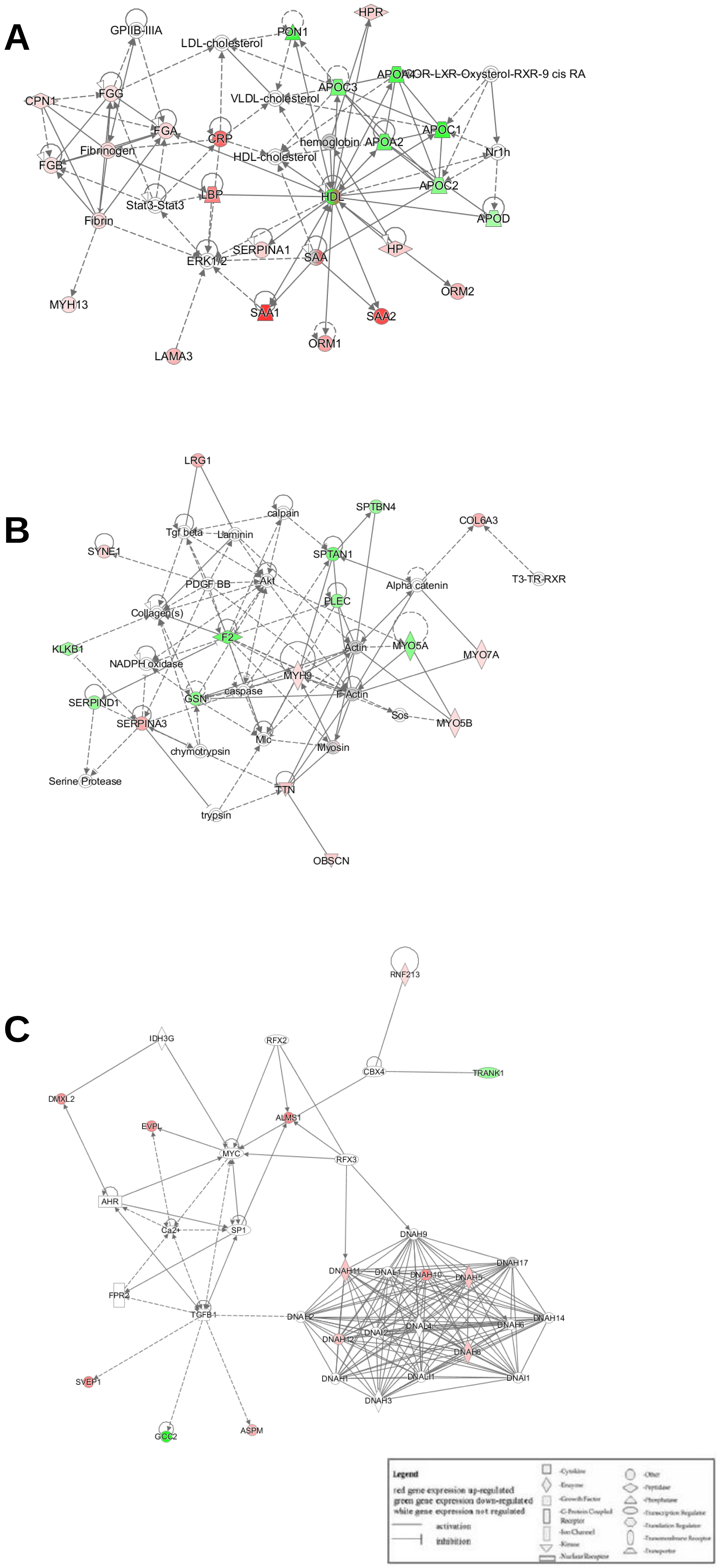

Fig. S2_1. Interaction network for day 0 survivors (D0S) where, A denote Metabolic Disease, Lipid Metabolism, Small Molecule Biochemistry, B, Cellular Assembly and Organization, Cellular Function and Maintenance, Cellular Movement and, C, developmental Disorder, Hereditary Disorder, Respiratory Disease. The green color represent down-regulation, red color represent up-regulation.

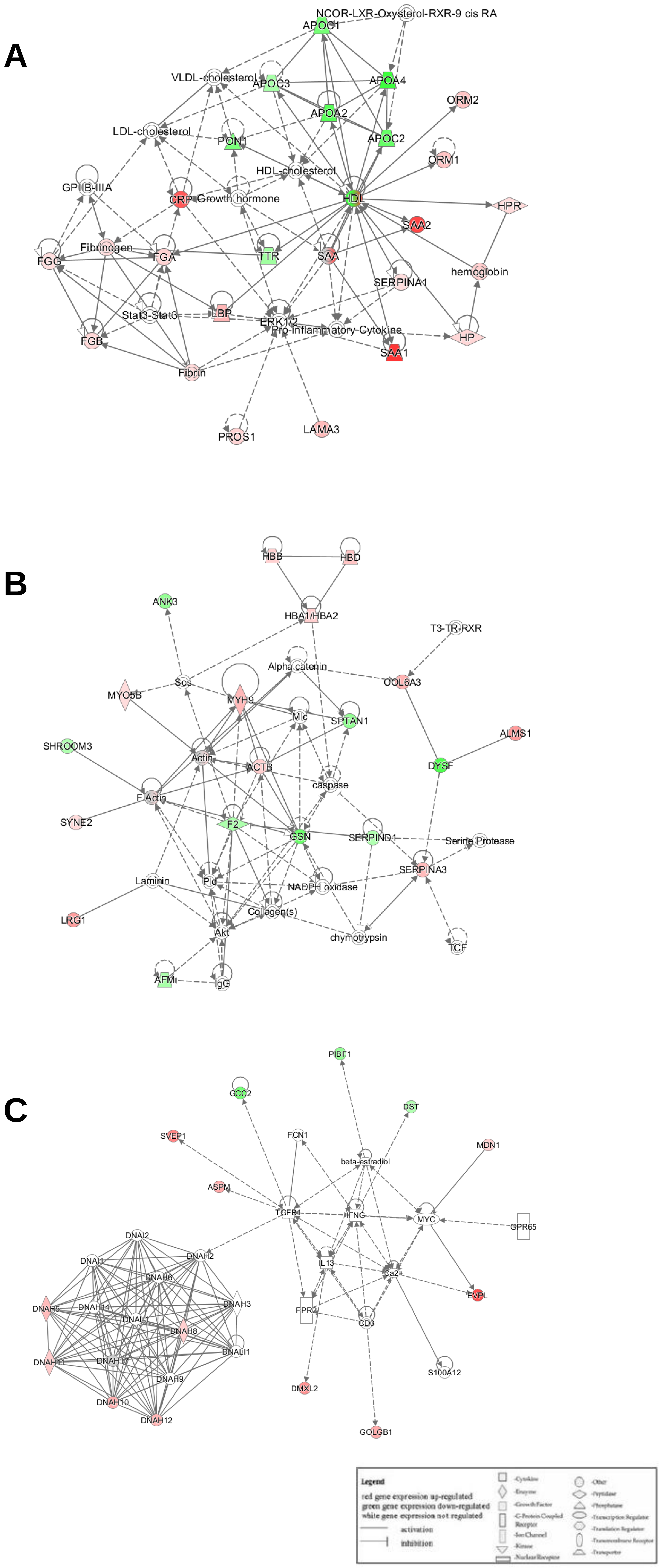

Fig.S2_2. Interaction network for day 0 non-survivors (D0NS) where, A denote Metabolic Disease, lipid Metabolism, Molecular Transport, B, Cell-to Cell Signaling and Interaction, Cellular Assembly and Organization, Connective Tissue Disorders, C, Developmental Disorder, Hereditary Disorder, Respiratory Disease. The green color represent down-regulation, red color represent up-regulation.

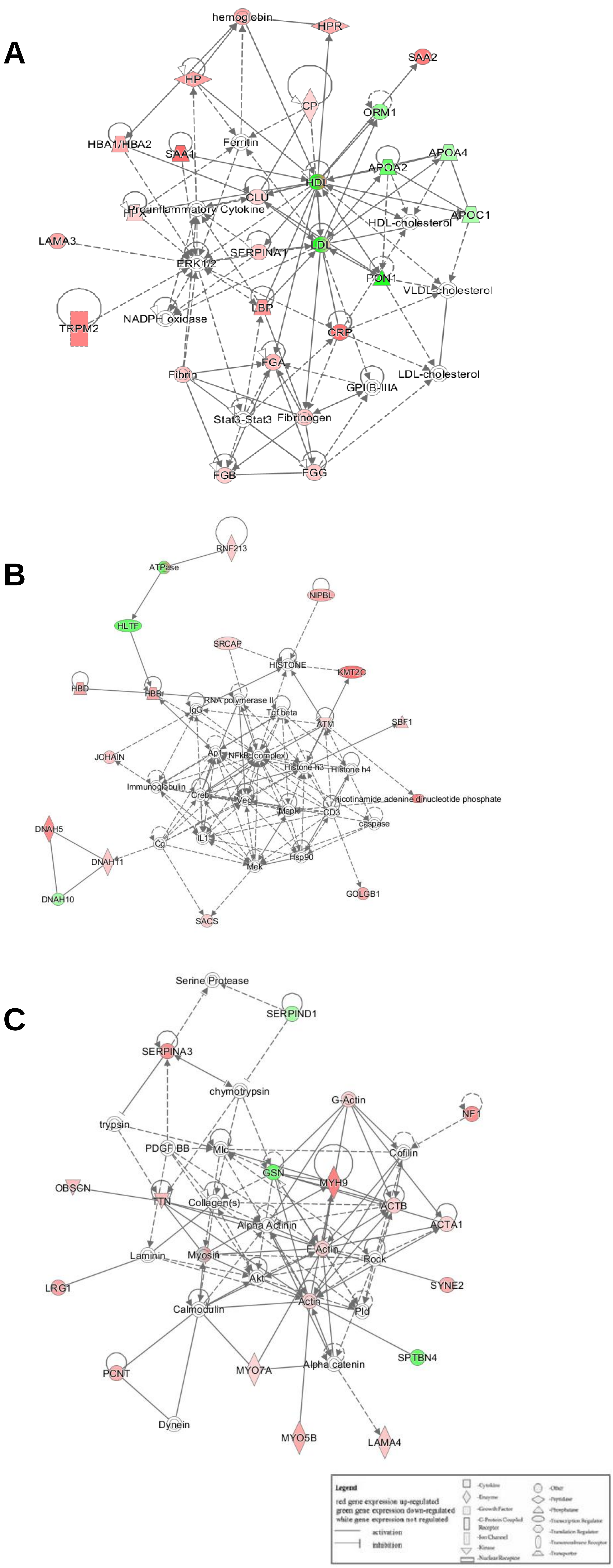

Fig.S2_3. Interaction network for day 7 survivors (D7S) where, A denote Metabolic Disease, Cardiovascular Disease, Developmental Disorder, B, Cancer, Organismal Injury arid Abnormalities, Endocrine System Disorders, C, Cellular Assembly and Organization, Cellular Development, Cellular Growth and Proliferation. The green color represent down-regulation, red color represent up-regulation.

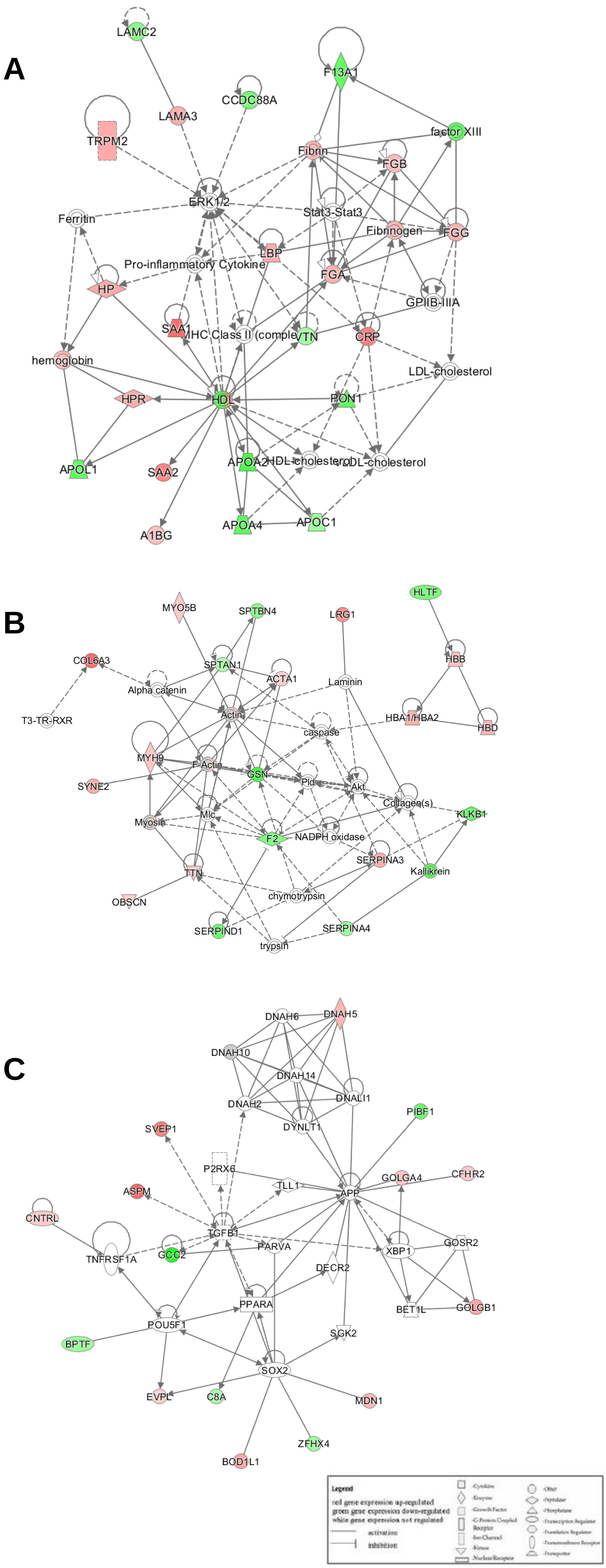

Fig.S2_4. Interaction network for day 7 non-survivors (D7NS) where, A denote Developmental Disorder, Hematological Disease, Hereditary Disorder, B, Cellular Assembly and Organization, Cellular Development, Cellular Growth and Proliferation, C, Cellular Development, Tissue Development, Cancer. The green color represent down-regulation, red color represent up-regulation.

Table S1. Quantitative proteomics data with protein description, number of peptides matched, Mascot score and fold changes in septic patients.

Protein Groups	Acces	Gene name	Protein description	Peptides matched	Score	D0A	D0D	D7A	D7D
CO3_HUMAN	P01024	C3	Complement C3	780	22992	-0.37707	-0.37707	-0.25154	-0.37707
A1AT_HUMAN	P01009	SERPINA1	Alpha-1-antitrypsin	897	19469	0.565597	0.389567	0.613532	0.263034
APOA1_HUMAN	P02647	APOA1	Apolipoprotein A-I	725	13962	-0.2863	-0.34008	0.163499	-0.05889
FIBA_HUMAN	P02671	FGA	Fibrinogen alpha chain	379	9810	0.536053	0.545968	0.695994	0.678072
SAA1_HUMAN	P0DJI8	SAA1	Serum amyloid A-1 protein	218	8379	2.462052	2.451541	1.475085	1.257011
CO4B_HUMAN	P0C0L5	C4B	Complement C4-B	323	8217	-0.34008	-0.34008	-0.32193	-0.37707
CO4A_HUMAN	P0C0L4	C4A	Complement C4-A	322	8014	-0.34008	-0.34008	-0.32193	-0.37707
SAA2_HUMAN	P0DJI9	SAA2	Serum amyloid A-2 protein	223	7805	2.179511	2.204767	1.286881	1.035624
A2MG_HUMAN	P01023	A2M	Alpha-2-macroglobulin	360	7199	-0.21759	0.163499	-0.20091	0.286881
FIBB_HUMAN	P02675	FGB	Fibrinogen beta chain	215	5741	0.422233	0.485427	0.485427	0.594549
CERU_HUMAN	P00450	CP	Ceruloplasmin	217	5622	-0.13606	0.014355	0.411426	0.378512
TRFE_HUMAN	P02787	TF	Serotransferrin	213	4252	-0.05889	-0.34008	-0.02915	-0.30401
AACT_HUMAN	P01011	SERPINA3	Alpha-1-antichymotrypsin	169	3946	1.035624	0.807355	1.111031	0.713696
APOB_HUMAN	P04114	APOB	Apolipoprotein B-100	201	3797	-0.35845	-0.18442	-0.30401	0
ITIH4_HUMAN	Q14624	ITIH4	Inter-alpha-trypsin inhibitor heavy chain H4	143	3563	0.097611	0.189034	0.344828	0.189034
APOA4_HUMAN	P06727	APOA4	Apolipoprotein A-IV	160	3136	-0.81097	-0.97143	-0.4344	-0.78588
CFAH_HUMAN	P08603	CFH	Complement factor H	164	2916	0.176323	-0.08927	0.321928	0.201634
HPT_HUMAN	P00738	HP	Haptoglobin	205	2778	0.613532	0.475085	0.895303	0.748461
VTNC_HUMAN	P04004	VTN	Vitronectin	103	2761	-0.04394	0.056584	-0.02915	-0.4344
A2GL_HUMAN	P02750	LRG1	Leucine-rich alpha-2-glycoprotein	115	2688	0.992768	1.163499	0.918386	0.978196
APOE_HUMAN	P02649	APOE	Apolipoprotein E	82	2465	-0.26882	-0.152	-0.074	-0.21759
FIBG_HUMAN	P02679	FGG	Fibrinogen gamma chain	127	2103	0.443607	0.389567	0.555816	0.678072
CFAB_HUMAN	P00751	CFB	Complement factor B	102	2086	0.15056	-0.08927	0.097611	-0.08927
ANGT_HUMAN	P01019	AGT	Angiotensinogen	59	2061	-0.02915	0.028569	0.189034	0
ANT3_HUMAN	P01008	SERPINC1	Antithrombin-III	106	2039	0	0.042644	0.333424	0.214125
HPTR_HUMAN	P00739	HPR	Haptoglobin-related protein	130	1917	0.594549	0.422233	0.815575	0.713696
THRB_HUMAN	P00734	F2	Prothrombin	85	1867	-0.68966	-0.41504	-0.68966	-0.62149
PLMN_HUMAN;PLGB_HUMAN	P00747	PLG	Plasminogen	79	1826	-0.08927	-0.18442	0.137504	0.028569
CLUS_HUMAN	P10909	CLU	Clusterin	65	1808	-0.13606	-0.05889	0.389567	0.176323
TTHY_HUMAN	P02766	TTR	Transthyretin	53	1609	-0.37707	-0.47393	-0.1047	-0.0145
HBB_HUMAN;HBE_HUMAN	P68871	HBB	Hemoglobin subunit beta	71	1590	-0.16812	0.545968	0.871844	0.555816
HEMO_HUMAN	P02790	HPX	Hemopexin	99	1558	0.097611	-0.05889	0.464668	0.137504
APOC3_HUMAN	P02656	APOC3	Apolipoprotein C-III	51	1549	-0.64386	-0.4344	-0.152	-0.25154
CRP_HUMAN	P02741	CRP	C-reactive protein	113	1512	1.85599	1.974529	1.400538	1.097611
PON1_HUMAN	P27169	PON1	Serum paraoxonase/arylesterase 1	56	1500	-0.8625	-0.66658	-1.08927	-0.78588
IGLL5_HUMAN;LAC1_HUMAN	B9A064	IGLL5	Immunoglobulin lambda-like polypeptide 5	68	1490	0.15056	0	0.226509	0.176323
CBPN_HUMAN	P15169	CPN1	Carboxypeptidase N catalytic chain	57	1362	0.454176	0.028569	0.137504	-0.074
APOA2_HUMAN	P02652	APOA2	Apolipoprotein A-II	125	1318	-0.71312	-0.8365	-0.73697	-0.8625
SAMP_HUMAN	P02743	APCS	Serum amyloid P-component	86	1277	-0.074	-0.32193	0.15056	-0.13606
GELS_HUMAN	P06396	GSN	Gelsolin	48	1058	-0.53533	-0.73697	-0.76121	-0.91594
APOD_HUMAN	P05090	APOD	Apolipoprotein D	50	970	-0.39593	-0.05889	0	0.189034
A1BG_HUMAN	P04217	A1BG	Alpha-1B-glycoprotein	40	969	0.226509	0.201634	0.214125	0.536053
CO5_HUMAN	P01031	C5	Complement C5	53	953	-0.25154	-0.152	0.214125	-0.02915
HBD_HUMAN	P02042	HBD	Hemoglobin subunit delta	37	828	-0.08927	0.704872	0.879706	0.659925
FHR1_HUMAN	Q03591	CFHR1	Complement factor H-related protein 1	33	743	0.124328	-0.02915	0.367371	0.378512
APOC2_HUMAN	P02655	APOC2	Apolipoprotein C-II	35	740	-0.53533	-0.76121	-0.18442	-0.37707
PZP_HUMAN	P20742	PZP	Pregnancy zone protein	55	718	-0.30401	0.15056	-0.30401	0.250962
C4BPA_HUMAN	P04003	C4BPA	C4b-binding protein alpha chain	36	716	0.238787	0.333424	-0.1047	0.137504
FBLN1_HUMAN	P23142	FBLN1	Fibulin-1	26	651	0.042644	-0.02915	0.214125	0.163499
LBP_HUMAN	P18428	LBP	Lipopolysaccharide-binding protein	22	631	1.505891	0.871844	1.042644	0.722466
VTDB_HUMAN	P02774	GC	Vitamin D-binding protein	39	602	0.201634	0.124328	0.201634	0.250962
AMBP_HUMAN	P02760	AMBP	Protein AMBP	12	470	-0.30401	-0.152	-0.12029	-0.18442
CD14_HUMAN	P08571	CD14	Monocyte differentiation antigen CD14	8	399	0.226509	0.111031	0.226509	-0.04394
IGJ_HUMAN	P01591	JCHAIN	Immunoglobulin J chain	13	378	0.163499	0.356144	0.594549	0.389567
CO2_HUMAN	P06681	C2	Complement C2	14	372	0.028569	-0.12029	-0.13606	0.124328
A2AP_HUMAN	P08697	SERPINF2	Alpha-2-antiplasmin	18	341	-0.0145	-0.18442	0.189034	0.070389
QSOX1_HUMAN	O00391	QSOX1	Sulfhydryl oxidase 1	17	336	0.189034	0.378512	0.422233	0.176323
ITIH2_HUMAN	P19823	ITIH2	Inter-alpha-trypsin inhibitor heavy chain H2	16	335	-0.30401	0.014355	-0.08927	-0.1047
APOC1_HUMAN	P02654	APOC1	Apolipoprotein C-I	37	330	-0.88897	-0.68966	-0.39593	-0.47393
CO8G_HUMAN	P07360	C8G	Complement component C8 gamma chain	15	328	0	0.084064	-0.25154	-0.30401
CO6_HUMAN	P13671	C6	Complement component C6	7	313	0.263034	0.042644	1.275007	0.286881
ITIH1_HUMAN	P19827	ITIH1	Inter-alpha-trypsin inhibitor heavy chain H1	19	300	-0.13606	-0.074	0.163499	-0.18442
CO9_HUMAN	P02748	C9	Complement component C9	25	291	0.097611	0.321928	0.367371	0.389567
CE350_HUMAN	Q5VT06	CEP350	Centrosome-associated protein 350	24	290	-0.18442	-0.20091	0.263034	0.028569
HRG_HUMAN	P04196	HRG	Histidine-rich glycoprotein	11	284	0.124328	-0.05889	0.097611	0.124328
TLN1_HUMAN	Q9Y490	TLN1	Talin-1	27	275	-0.08927	-0.05889	0.263034	-0.152
DYH8_HUMAN	Q96JB1	DNAH8	Dynein heavy chain 8, axonemal	21	270	0.669027	0.565597	0.124328	0.014355
CO8A_HUMAN	P07357	C8A	Complement component C8 alpha chain	10	267	-0.04394	-0.08927	-0.25154	-0.41504
OBSCN_HUMAN	Q5VST9	OBSCN	Obscurin	20	252	0.613532	0.31034	0.659925	0.411426
A1AG1_HUMAN	P02763	ORM1	Alpha-1-acid glycoprotein 1	19	243	1.144046	0.704872	-0.51457	-0.074
ACTB_HUMAN;ACTG_HUMAN;AC	P60709	ACTB	Actin, cytoplasmic 1	9	241	0.344828	0.565597	0.495695	0.163499
LAMA3_HUMAN	Q16787	LAMA3	Laminin subunit alpha-3	13	233	0.903038	0.807355	0.871844	0.704872
MYO5B_HUMAN	Q9ULV0	MYO5B	Unconventional myosin-Vb	16	228	0.411426	0.411426	0.807355	0.454176
APOL1_HUMAN	O14791	APOL1	Apolipoprotein L1	11	224	-0.20091	-0.32193	0.15056	-0.73697
DYH5_HUMAN	Q8TE73	DNAH5	Dynein heavy chain 5, axonemal	21	215	0.704872	0.815575	1.124328	0.641546
DYST_HUMAN	Q03001	DST	Dystonin	28	213	-0.35845	-0.39593	0.070389	-0.23447
HEP2_HUMAN	P05546	SERPIND1	Heparin cofactor 2	14	210	-0.47393	-0.39593	-0.45403	-0.68966
FHR2_HUMAN	P36980	CFHR2	Complement factor H-related protein 2	21	209	0.137504	0.028569	0.400538	0.464668
SYNE1_HUMAN	Q8NF91	SYNE1	Nesprin-1	18	203	0.555816	0.111031	0.333424	0.097611
LUM_HUMAN	P51884	LUM	Lumican	14	202	-0.04394	-0.16812	0.097611	-0.08927
TITIN_HUMAN	Q8WZ42	TTN	Titin	18	197	0.731183	0.367371	0.678072	0.432959
PEDF_HUMAN	P36955	SERPINF1	Pigment epithelium-derived factor	12	194	0.097611	-0.02915	-0.0145	0
KNG1_HUMAN	P01042	KNG1	Kininogen-1	8	188	0.084064	0.028569	0.111031	-0.0145
ITIH3_HUMAN	Q06033	ITIH3	Inter-alpha-trypsin inhibitor heavy chain H3	8	176	-0.0145	0.097611	0.042644	-0.05889
C1R_HUMAN	P00736	C1R	Complement C1r subcomponent	7	171	0.263034	0.632268	0.263034	0.286881
C1S_HUMAN	P09871	C1S	Complement C1s subcomponent	10	169	0.124328	0.056584	0.226509	0.263034
IC1_HUMAN	P05155	SERPING1	Plasma protease C1 inhibitor	10	169	0.163499	0.378512	-0.074	0.378512
CFAI_HUMAN	P05156	CFI	Complement factor I	8	164	-0.05889	-0.04394	0.189034	0.485427
DYH17_HUMAN	Q9UFH2	DNAH17	Dynein heavy chain 17, axonemal	17	160	-0.35845	-0.2863	0.250962	-0.05889
GPX3_HUMAN;GPX6_HUMAN	P22352	GPX3	Glutathione peroxidase 3	23	159	0.333424	0.137504	0.432959	0.189034
SPTA1_HUMAN	P02549	SPTA1	Spectrin alpha chain, erythrocytic 1	9	154	-0.2863	-0.30401	0.333424	0.084064
VP13B_HUMAN	Q7Z7G8	VPS13B	Vacuolar protein sorting-associated protein 13B	8	151	-0.18442	-0.34008	0	0
SAA4_HUMAN	P35542	SAA4	Serum amyloid A-4 protein	14	151	-0.16812	0.084064	-0.02915	-0.152
HLTF_HUMAN	Q14527	HLTF	Helicase-like transcription factor	18	146	0.056584	-0.21759	-0.76121	-0.64386
CE290_HUMAN	O15078	CEP290	Centrosomal protein of 290 kDa	21	142	0.042644	0.070389	0.378512	-0.08927
HBA_HUMAN;HBAZ_HUMAN	P69905	HBA1	Hemoglobin subunit alpha	15	142	-0.20091	0.565597	0.85599	0.687061
CO8B_HUMAN	P07358	C8B	Complement component C8 beta chain	6	138	-0.0145	-0.12029	-0.30401	-0.20091
PROS_HUMAN	P07225	PROS1	Vitamin K-dependent protein S	7	137	0.084064	0.505891	0.286881	-0.08927
GOGB1_HUMAN	Q14789	GOLGB1	Golgin subfamily B member 1	9	135	0.632268	1.007196	0.879706	0.773996
CNTRL_HUMAN	Q7Z7A1	CNTRL	Centriolin	6	134	0.014355	0.111031	0.443607	0.454176
SMC3_HUMAN	Q9UQE7	SMC3	Structural maintenance of chromosomes protein 3	6	131	0.454176	0.014355	0.201634	0.189034
SACS_HUMAN	Q9NZJ4	SACS	Sacsin	10	126	-0.25154	-0.35845	0.495695	0.263034
RN213_HUMAN	Q63HN8	RNF213	E3 ubiquitin-protein ligase RNF213	10	122	0.443607	0.250962	0.505891	0.275007
KAIN_HUMAN	P29622	SERPINA4	Kallistatin	6	118	-0.21759	-0.2863	-0.0145	-0.47393
CE112_HUMAN	Q8N8E3	CEP112	Centrosomal protein of 112 kDa	6	113	-0.1047	0.070389	-0.21759	-0.2863
PERQ1_HUMAN	O75420	GIGYF1	PERQ amino acid-rich with GYF domain-containing protein 1	8	103	-0.20091	-0.152	-0.30401	-0.35845
DMXL2_HUMAN	Q8TDJ6	DMXL2	DmX-like protein 2	7	102	1.372952	1.298658	0.879706	1.042644
MACF1_HUMAN	Q9UPN3	MACF1	Microtubule-actin cross-linking factor 1, isoforms 1/2/3/5	9	99	0.201634	0	0.15056	0.097611
K1109_HUMAN	Q2LD37	KIAA1109	Uncharacterized protein KIAA1109	5	90	0.594549	-0.05889	0.321928	0.124328
DYSF_HUMAN	O75923	DYSF	Dysferlin	5	90	-0.04394	-0.88897	-0.18442	-0.32193
NF1_HUMAN	P21359	NF1	Neurofibromin	7	89	1.232661	1.333424	1.104337	1.042644
LAMC2_HUMAN	Q13753	LAMC2	Laminin subunit gamma-2	7	89	-0.35845	0.028569	-0.30401	-0.57777
DYH11_HUMAN	Q96DT5	DNAH11	Dynein heavy chain 11, axonemal	11	88	0.722466	0.62293	0.536053	0.378512
PIBF1_HUMAN	Q8WXW3	PIBF1	Progesterone-induced-blocking factor 1	10	85	-0.23447	-0.53533	-0.25154	-0.71312
CROCC_HUMAN;CROL2_HUMAN	Q5TZA2	CROCC	Rootletin	6	84	-0.37707	-0.20091	-0.21759	-0.32193
A1AG2_HUMAN	P19652	ORM2	Alpha-1-acid glycoprotein 2	11	82	0.925999	0.704872	-0.35845	-0.12029
KLKB1_HUMAN	P03952	KLKB1	Plasma kallikrein	11	80	-0.4344	-0.37707	-0.34008	-0.73697
AFAM_HUMAN	P43652	AFM	Afamin	4	79	-0.152	-0.4344	-0.2863	-0.35845
DYH12_HUMAN	Q6ZR08	DNAH12	Dynein heavy chain 12, axonemal	5	79	0.722466	0.871844	-0.37707	-0.16812
ACTS_HUMAN	P68133	ACTA1	Actin, alpha skeletal muscle	4	77	0.356144	0.275007	0.454176	0.411426
ANR26_HUMAN	Q9UPS8	ANKRD26	Ankyrin repeat domain-containing protein 26	4	76	-0.25154	-0.08927	-0.04394	-0.05889
FSIP2_HUMAN	Q5CZC0	FSIP2	Fibrous sheath-interacting protein 2	6	73	-0.64386	-1.12029	-0.25154	-0.25154
ANK3_HUMAN	Q12955	ANK3	Ankyrin-3	6	73	-0.2863	-0.53533	0.014355	-0.23447
SVEP1_HUMAN	Q4LDE5	SVEP1	Sushi, von Willebrand factor type A, EGF and pentraxin domain-containing protein 1	4	69	1.516015	1.448901	1.195348	1.117695
GOGA4_HUMAN	Q13439	GOLGA4	Golgin subfamily A member 4	11	68	0.163499	0.070389	0.214125	0.516015
POTEI_HUMAN	P0CG38	POTEI	POTE ankyrin domain family member I	3	68	0.298658	0.275007	0.454176	0.15056
POTEE_HUMAN	Q6S8J3	POTEE	POTE ankyrin domain family member E	3	68	0.298658	0.275007	0.454176	0.15056
POTEF_HUMAN	A5A3E0	POTEF	POTE ankyrin domain family member F	3	68	0.298658	0.275007	0.454176	0.15056
SYCP2_HUMAN	Q9BX26	SYCP2	Synaptonemal complex protein 2	9	68	-0.13606	0.028569	0.443607	0.084064
SHRM3_HUMAN	Q8TF72	SHROOM3	Protein Shroom3	7	67	-0.12029	-0.39593	-0.26882	-0.02915
KMT2C_HUMAN	Q8NEZ4	KMT2C	Histone-lysine N-methyltransferase 2C	5	66	1.769772	1.521051	1.411426	1.201634
CO6A3_HUMAN	P12111	COL6A3	Collagen alpha-3(VI) chain	4	64	0.978196	0.903038	0.887525	1.275007
CLIP1_HUMAN	P30622	CLIP1	CAP-Gly domain-containing linker protein 1	4	64	-0.0145	0.214125	0.070389	0.176323
NUMA1_HUMAN	Q14980	NUMA1	Nuclear mitotic apparatus protein 1	4	58	-0.59946	-0.68966	-0.30401	-0.91594
TRNK1_HUMAN	O15050	TRANK1	TPR and ankyrin repeat-containing protein 1	4	58	-0.45403	-0.64386	-0.13606	-0.05889
DAPLE_HUMAN	Q9P219	CCDC88C	Protein Daple	9	56	0.042644	0.333424	0.432959	0.137504
SYNE2_HUMAN	Q8WXH0	SYNE2	Nesprin-2	8	55	0.298658	0.411426	0.823749	0.704872
PCNT_HUMAN	O95613	PCNT	Pericentrin	3	55	0.084064	0.443607	0.799087	0.275007
MYO7B_HUMAN	Q6PIF6	MYO7B	Unconventional myosin-VIIb	3	51	0.176323	-0.18442	0.084064	-0.05889
F13A_HUMAN	P00488	F13A1	Coagulation factor XIII A chain	2	51	0.137504	-0.16812	-0.02915	-0.73697
POTEJ_HUMAN	P0CG39	POTEJ	POTE ankyrin domain family member J	2	50	0.333424	0.201634	0.321928	-0.18442
PDZD2_HUMAN	O15018	PDZD2	PDZ domain-containing protein 2	3	47	0.286881	0.367371	-0.0145	-0.25154
DESP_HUMAN	P15924	DSP	Desmoplakin	4	44	0.070389	0.31034	-0.20091	-0.13606
MYO5A_HUMAN	Q9Y4I1	MYO5A	Unconventional myosin-Va	3	44	-0.51457	-0.16812	0.15056	-0.26882
PLEC_HUMAN	Q15149	PLEC	Plectin	3	42	-0.49411	0.028569	-0.25154	-0.20091
PCM1_HUMAN	Q15154	PCM1	Pericentriolar material 1 protein	3	41	0.163499	-0.26882	-0.78588	-0.8365
GRDN_HUMAN	Q3V6T2	CCDC88A	Girdin	6	41	0.238787	-0.25154	-0.30401	-0.66658
GCC2_HUMAN	Q8IWJ2	GCC2	GRIP and coiled-coil domain-containing protein 2	3	40	-1	-0.68966	-0.41504	-1.05889
HUWE1_HUMAN;F71E1_HUMAN	Q7Z6Z7	HUWE1	E3 ubiquitin-protein ligase HUWE1	3	39	0.250962	-0.26882	-0.0145	0.028569
BPTF_HUMAN	Q12830	BPTF	Nucleosome-remodeling factor subunit BPTF	4	39	-0.18442	-0.32193	-0.41504	-0.47393
MICA3_HUMAN	Q7RTP6	MICAL3	Protein-methionine sulfoxide oxidase MICAL3	3	39	-0.12029	0	-0.12029	0.111031
ZFHX4_HUMAN	Q86UP3	ZFHX4	Zinc finger homeobox protein 4	3	39	0.15056	-0.21759	0.263034	-0.47393
MYO7A_HUMAN	Q13402	MYO7A	Unconventional myosin-VIIa	5	39	0.400538	0.163499	0.411426	0.189034
SPTN1_HUMAN	Q13813	SPTAN1	Spectrin alpha chain, non-erythrocytic 1	4	38	-0.64386	-0.55639	-0.2863	-0.39593
DYH10_HUMAN	Q8IVF4	DNAH10	Dynein heavy chain 10, axonemal	2	38	1.31034	0.941106	-0.45403	-0.25154
UTP20_HUMAN;HEXI1_HUMAN	O94992	HEXIM1	Protein HEXIM1	3	38	1.575312	1.613532	1.475085	0.807355
NIPBL_HUMAN	Q6KC79	NIPBL	Nipped-B-like protein	6	36	0.367371	0.575312	0.85599	0.903038
MYH9_HUMAN	P35579	MYH9	Myosin-9	2	35	0.400538	0.831877	1.226509	0.485427
ALMS1_HUMAN	Q8TCU4	ALMS1	Alstrom syndrome protein 1	2	35	1.521051	1.201634	-0.21759	0.176323
ASPM_HUMAN	Q8IZT6	ASPM	Abnormal spindle-like microcephaly-associated protein	3	34	0.956057	1.028569	0.85599	1.31034
KIF27_HUMAN	Q86VH2	KIF27	Kinesin-like protein KIF27	2	34	1.726831	1.232661	2.025029	1.773996
SPTN4_HUMAN	Q9H254	SPTBN4	Spectrin beta chain, non-erythrocytic 4	3	33	-0.47393	-0.1047	-0.76121	-0.57777
AHNK2_HUMAN	Q8IVF2	AHNAK2	Protein AHNAK2	5	32	-0.30401	0.31034	-0.08927	-0.1047
MYH4_HUMAN	Q9Y623	MYH4	Myosin-4	5	32	0.014355	-0.12029	-0.30401	-0.12029
MTMR5_HUMAN	O95248	SBF1	Myotubularin-related protein 5	3	32	-0.25154	-0.55639	0.594549	-0.39593
MYH7B_HUMAN	A7E2Y1	MYH7B	Myosin-7B	3	23	0.137504	0.028569	-0.152	-0.37707
SRCAP_HUMAN	Q6ZRS2	SRCAP	Helicase SRCAP	2	22	0.475085	0.454176	0.443607	0.516015
MYO9A_HUMAN	B2RTY4	MYO9A	Unconventional myosin-IXa	4	21	0.31034	-0.1047	0.485427	0.275007
ATM_HUMAN	Q13315	ATM	Serine-protein kinase ATM	6	21	0.475085	0.176323	0.485427	0.565597
TRPM2_HUMAN	O94759	TRPM2	Transient receptor potential cation channel subfamily M member 2	2	20	0.028569	0.201634	1.214125	0.722466
CENPE_HUMAN	Q02224	CENPE	Centromere-associated protein E	2	18	0.526069	-0.23447	0.85599	0.344828
PRC2C_HUMAN	Q9Y520	PRRC2C	Protein PRRC2C	2	18	0.070389	0.176323	0.137504	0.275007
BD1L1_HUMAN	Q8NFC6	BOD1L1	Biorientation of chromosomes in cell division protein 1-like 1	4	18	0.137504	0.070389	0.411426	0.765535
EVPL_HUMAN	Q92817	EVPL	Envoplakin	4	17	1.356144	2.124328	0.895303	0.389567
LYST_HUMAN	Q99698	LYST	Lysosomal-trafficking regulator	11	17	-0.25154	-0.0145	-0.20091	-0.51457
MDN1_HUMAN	Q9NU22	MDN1	Midasin	4	16	0.333424	0.575312	0.879706	0.62293
MYH13_HUMAN	Q9UKX3	MYH13	Myosin-13	5	16	0.400538	0.070389	0.378512	0.321928
CC146_HUMAN	Q8IYE0	CCDC146	Coiled-coil domain-containing protein 146	10	16	-0.30401	-0.23447	-0.30401	-0.35845
LAMA4_HUMAN	Q16363	LAMA4	Laminin subunit alpha-4	5	16	0.263034	0.356144	0.536053	0.176323

Table S2. Detailed gene ontology annotations for septic patients for molecular function, biological processes and cellular components.

Table S2.1 Survivors patients D0 samples.

Category	ID	Name	p-value	q-value Bonferroni	q-value FDR B&H	q-value FDR B&Y	Hit Count in Query List	Hit Count in Genome	Hit in Query List
GO: Molecular Function	GO:0003774	motor activity	3.86E-16	8.61E-14	8.61E-14	5.16E-13	13	130	MYH9,DNAH12,KIF27,MYH13,DNAH10,MYO5A,MYO5B,SMC3,CENPE,MYO7A,DNAH5,DNAH8,DNAH11
GO: Molecular Function	GO:0003777	microtubule motor activity	1.88E-10	4.20E-08	2.10E-08	1.26E-07	8	77	DNAH12,KIF27,DNAH10,SMC3,CENPE,DNAH5,DNAH8,DNAH11
GO: Molecular Function	GO:0008092	cytoskeletal protein binding	6.42E-10	1.43E-07	4.77E-08	2.86E-07	17	792	SPTBN4,MYH9,NF1,KIF27,MYH13,ALMS1,MYO5A,MYO5B,CENPE,MYO7A,SYNE1,SPTAN1,NUMA1,OBSCN,PLEC,TTN,GSN
GO: Molecular Function	GO:0005516	calmodulin binding	7.06E-09	1.57E-06	3.27E-07	1.96E-06	9	175	MYH9,MYH13,MYO5A,MYO5B,MYO7A,SPTAN1,OBSCN,ASPM,TTN
GO: Molecular Function	GO:0000146	microfilament motor activity	7.34E-09	1.64E-06	3.27E-07	1.96E-06	5	21	MYH9,MYH13,MYO5A,MYO5B,MYO7A
GO: Molecular Function	GO:0017111	nucleoside-triphosphatase activity	3.48E-08	7.77E-06	1.29E-06	7.75E-06	15	781	MYH9,DNAH12,KIF27,MYH13,DNAH10,MYO5A,MYO5B,SMC3,CENPE,MYO7A,RNF213,SRCAP,DNAH5,DNAH8,DNAH11
GO: Molecular Function	GO:0043178	alcohol binding	4.80E-08	1.07E-05	1.45E-06	8.68E-06	7	101	NF1,APOA2,APOA4,APOC1,APOC3,APOD,CRP
GO: Molecular Function	GO:0016462	pyrophosphatase activity	6.71E-08	1.50E-05	1.45E-06	8.68E-06	15	821	MYH9,DNAH12,KIF27,MYH13,DNAH10,MYO5A,MYO5B,SMC3,CENPE,MYO7A,RNF213,SRCAP,DNAH5,DNAH8,DNAH11
GO: Molecular Function	GO:0016818	hydrolase activity, acting on acid anhydrides, in phosphorus-containing anhydrides	6.93E-08	1.55E-05	1.45E-06	8.68E-06	15	823	MYH9,DNAH12,KIF27,MYH13,DNAH10,MYO5A,MYO5B,SMC3,CENPE,MYO7A,RNF213,SRCAP,DNAH5,DNAH8,DNAH11
GO: Molecular Function	GO:0003779	actin binding	7.13E-08	1.59E-05	1.45E-06	8.68E-06	11	392	SPTBN4,MYH9,MYH13,MYO5A,MYO5B,MYO7A,SYNE1,SPTAN1,PLEC,TTN,GSN
GO: Molecular Function	GO:0016817	hydrolase activity, acting on acid anhydrides	7.15E-08	1.60E-05	1.45E-06	8.68E-06	15	825	MYH9,DNAH12,KIF27,MYH13,DNAH10,MYO5A,MYO5B,SMC3,CENPE,MYO7A,RNF213,SRCAP,DNAH5,DNAH8,DNAH11
GO: Molecular Function	GO:0050997	quaternary ammonium group binding	1.51E-07	3.36E-05	2.80E-06	1.68E-05	5	37	NF1,APOA2,APOA4,APOC1,CRP
GO: Molecular Function	GO:0005524	ATP binding	2.17E-07	4.85E-05	3.73E-06	2.23E-05	19	1476	MYH9,TRANK1,DNAH12,KIF27,MYH13,DNAH10,MYO5A,MYO5B,SMC3,CENPE,MYO7A,OBSCN,RNF213,ATM,SRCAP,DNAH5,DNAH8,TTN,DNAH11
GO: Molecular Function	GO:0031210	phosphatidylcholine binding	2.73E-07	6.10E-05	4.36E-06	2.61E-05	4	17	NF1,APOA2,APOA4,APOC1
GO: Molecular Function	GO:0032559	adenyl ribonucleotide binding	3.33E-07	7.42E-05	4.81E-06	2.88E-05	19	1517	MYH9,TRANK1,DNAH12,KIF27,MYH13,DNAH10,MYO5A,MYO5B,SMC3,CENPE,MYO7A,OBSCN,RNF213,ATM,SRCAP,DNAH5,DNAH8,TTN,DNAH11
GO: Molecular Function	GO:0055102	lipase inhibitor activity	3.51E-07	7.82E-05	4.81E-06	2.88E-05	4	18	APOA2,APOC1,APOC2,APOC3
GO: Molecular Function	GO:0060228	phosphatidylcholine-sterol O-acyltransferase activa	3.67E-07	8.18E-05	4.81E-06	2.88E-05	3	5	APOA2,APOA4,APOC1
GO: Molecular Function	GO:0030554	adenyl nucleotide binding	4.03E-07	8.99E-05	4.99E-06	2.99E-05	19	1536	MYH9,TRANK1,DNAH12,KIF27,MYH13,DNAH10,MYO5A,MYO5B,SMC3,CENPE,MYO7A,OBSCN,RNF213,ATM,SRCAP,DNAH5,DNAH8,TTN,DNAH11
GO: Molecular Function	GO:0016887	ATPase activity	1.00E-06	2.24E-04	1.18E-05	7.05E-05	10	407	MYH9,DNAH12,KIF27,DNAH10,CENPE,MYO7A,RNF213,DNAH5,DNAH8,DNAH11
GO: Molecular Function	GO:0004857	enzyme inhibitor activity	2.20E-06	4.91E-04	2.46E-05	1.47E-04	9	344	SERPINA3,COL6A3,SERPINA1,APOA2,APOC1,APOC2,APOC3,SERPIND1,HEXIM1
GO: Molecular Function	GO:0035639	purine ribonucleoside triphosphate binding	4.79E-06	1.07E-03	5.09E-05	3.04E-04	19	1810	MYH9,TRANK1,DNAH12,KIF27,MYH13,DNAH10,MYO5A,MYO5B,SMC3,CENPE,MYO7A,OBSCN,RNF213,ATM,SRCAP,DNAH5,DNAH8,TTN,DNAH11
GO: Molecular Function	GO:0032550	purine ribonucleoside binding	5.32E-06	1.19E-03	5.10E-05	3.06E-04	19	1823	MYH9,TRANK1,DNAH12,KIF27,MYH13,DNAH10,MYO5A,MYO5B,SMC3,CENPE,MYO7A,OBSCN,RNF213,ATM,SRCAP,DNAH5,DNAH8,TTN,DNAH11
GO: Molecular Function	GO:0001883	purine nucleoside binding	5.45E-06	1.22E-03	5.10E-05	3.06E-04	19	1826	MYH9,TRANK1,DNAH12,KIF27,MYH13,DNAH10,MYO5A,MYO5B,SMC3,CENPE,MYO7A,OBSCN,RNF213,ATM,SRCAP,DNAH5,DNAH8,TTN,DNAH11
GO: Molecular Function	GO:0032549	ribonucleoside binding	5.49E-06	1.23E-03	5.10E-05	3.06E-04	19	1827	MYH9,TRANK1,DNAH12,KIF27,MYH13,DNAH10,MYO5A,MYO5B,SMC3,CENPE,MYO7A,OBSCN,RNF213,ATM,SRCAP,DNAH5,DNAH8,TTN,DNAH11
GO: Molecular Function	GO:0001882	nucleoside binding	5.95E-06	1.33E-03	5.31E-05	3.18E-04	19	1837	MYH9,TRANK1,DNAH12,KIF27,MYH13,DNAH10,MYO5A,MYO5B,SMC3,CENPE,MYO7A,OBSCN,RNF213,ATM,SRCAP,DNAH5,DNAH8,TTN,DNAH11
GO: Molecular Function	GO:0008289	lipid binding	6.34E-06	1.41E-03	5.44E-05	3.26E-04	11	619	SPTBN4,NF1,PON1,APOA2,APOA4,APOC1,APOC2,APOC3,LBP,APOD,CRP
GO: Molecular Function	GO:0032555	purine ribonucleotide binding	6.97E-06	1.55E-03	5.76E-05	3.45E-04	19	1857	MYH9,TRANK1,DNAH12,KIF27,MYH13,DNAH10,MYO5A,MYO5B,SMC3,CENPE,MYO7A,OBSCN,RNF213,ATM,SRCAP,DNAH5,DNAH8,TTN,DNAH11
GO: Molecular Function	GO:0032553	ribonucleotide binding	7.90E-06	1.76E-03	6.18E-05	3.70E-04	19	1873	MYH9,TRANK1,DNAH12,KIF27,MYH13,DNAH10,MYO5A,MYO5B,SMC3,CENPE,MYO7A,OBSCN,RNF213,ATM,SRCAP,DNAH5,DNAH8,TTN,DNAH11
GO: Molecular Function	GO:0015485	cholesterol binding	8.04E-06	1.79E-03	6.18E-05	3.70E-04	4	38	APOA2,APOC3,APOD,CRP
GO: Molecular Function	GO:0017076	purine nucleotide binding	8.34E-06	1.86E-03	6.20E-05	3.71E-04	19	1880	MYH9,TRANK1,DNAH12,KIF27,MYH13,DNAH10,MYO5A,MYO5B,SMC3,CENPE,MYO7A,OBSCN,RNF213,ATM,SRCAP,DNAH5,DNAH8,TTN,DNAH11
GO: Molecular Function	GO:0005198	structural molecule activity	8.82E-06	1.97E-03	6.35E-05	3.80E-04	11	641	SPTBN4,SPTAN1,NUMA1,OBSCN,FGA,FGB,LAMA3,EVPL,FGG,PLEC,TTN
GO: Molecular Function	GO:0032934	sterol binding	1.21E-05	2.69E-03	8.41E-05	5.04E-04	4	42	APOA2,APOC3,APOD,CRP
GO: Molecular Function	GO:0070653	high-density lipoprotein particle receptor binding	3.37E-05	7.52E-03	2.28E-04	1.36E-03	2	3	APOA2,APOC3
GO: Molecular Function	GO:0030506	ankyrin binding	4.70E-05	1.05E-02	3.08E-04	1.84E-03	3	21	SPTBN4,OBSCN,PLEC
GO: Molecular Function	GO:0005543	phospholipid binding	8.38E-05	1.87E-02	5.34E-04	3.20E-03	7	311	SPTBN4,NF1,PON1,APOA2,APOA4,APOC1,APOC3
GO: Molecular Function	GO:0071813	lipoprotein particle binding	9.07E-05	2.02E-02	5.47E-04	3.27E-03	3	26	PON1,APOA2,CRP
GO: Molecular Function	GO:0071814	protein-lipid complex binding	9.07E-05	2.02E-02	5.47E-04	3.27E-03	3	26	PON1,APOA2,CRP
GO: Molecular Function	GO:0030507	spectrin binding	1.14E-04	2.54E-02	6.67E-04	4.00E-03	3	28	SPTBN4,MYO7A,SPTAN1
GO: Molecular Function	GO:0046983	protein dimerization activity	1.23E-04	2.74E-02	7.03E-04	4.21E-03	13	1172	MYH9,MYO5A,SMC3,MYO7A,HP,SYNE1,SPTAN1,PON1,APOA2,APOA4,APOC2,ATM,CRP
GO: Molecular Function	GO:0030234	enzyme regulator activity	2.10E-04	4.67E-02	1.17E-03	6.99E-03	11	909	SERPINA3,COL6A3,SERPINA1,NF1,APOA2,APOA4,APOC1,APOC2,APOC3,SERPIND1,HEXIM1
GO: Molecular Function	GO:0032403	protein complex binding	2.41E-04	5.38E-02	1.31E-03	7.86E-03	11	924	MYH9,NF1,KIF27,SMC3,CENPE,MYO7A,SYNE1,SPTAN1,NUMA1,ATM,TTN
GO: Molecular Function	GO:0005496	steroid binding	2.55E-04	5.68E-02	1.35E-03	8.10E-03	4	91	APOA2,APOC3,APOD,CRP
GO: Molecular Function	GO:0030492	hemoglobin binding	3.11E-04	6.94E-02	1.58E-03	9.48E-03	2	8	HP,HPR
GO: Molecular Function	GO:0004867	serine-type endopeptidase inhibitor activity	3.13E-04	6.97E-02	1.58E-03	9.48E-03	4	96	SERPINA3,COL6A3,SERPINA1,SERPIND1
GO: Biological Process	GO:0006953	acute-phase response	8.78E-16	1.75E-12	1.75E-12	1.43E-11	10	49	SERPINA3,ORM1,ORM2,SAA1,SERPINA1,SAA2,HP,LBP,F2,CRP
GO: Biological Process	GO:0002526	acute inflammatory response	5.19E-14	1.03E-10	5.17E-11	4.22E-10	12	140	SERPINA3,ORM1,ORM2,SAA1,SERPINA1,SAA2,HP,APOA2,LBP,F2,KLKB1,CRP
GO: Biological Process	GO:0009611	response to wounding	8.51E-11	1.69E-07	5.65E-08	4.62E-07	22	1255	SERPINA3,ORM1,ORM2,SAA1,SERPINA1,SAA2,NF1,CENPE,HP,FGA,FGB,APOA2,APOC3,LBP,FGG,APOD,F2,TTN,KLKB1,SERPIND1,GSN,CRP
GO: Biological Process	GO:0033700	phospholipid efflux	3.30E-10	6.57E-07	1.64E-07	1.34E-06	5	12	APOA2,APOA4,APOC1,APOC2,APOC3
GO: Biological Process	GO:0032374	regulation of cholesterol transport	2.04E-09	4.05E-06	6.76E-07	5.52E-06	6	35	PON1,APOA2,APOA4,APOC1,APOC2,APOC3
GO: Biological Process	GO:0032371	regulation of sterol transport	2.04E-09	4.05E-06	6.76E-07	5.52E-06	6	35	PON1,APOA2,APOA4,APOC1,APOC2,APOC3
GO: Biological Process	GO:0033344	cholesterol efflux	4.75E-09	9.46E-06	1.35E-06	1.10E-05	6	40	PON1,APOA2,APOA4,APOC1,APOC2,APOC3
GO: Biological Process	GO:0006954	inflammatory response	1.30E-08	2.60E-05	2.47E-06	2.02E-05	14	599	SERPINA3,ORM1,ORM2,SAA1,SERPINA1,SAA2,HP,APOA2,APOC3,LBP,APOD,F2,KLKB1,CRP
GO: Biological Process	GO:0034369	plasma lipoprotein particle remodeling	1.36E-08	2.71E-05	2.47E-06	2.02E-05	5	23	APOA2,APOA4,APOC1,APOC2,APOC3
GO: Biological Process	GO:0034368	protein-lipid complex remodeling	1.36E-08	2.71E-05	2.47E-06	2.02E-05	5	23	APOA2,APOA4,APOC1,APOC2,APOC3
GO: Biological Process	GO:0034367	macromolecular complex remodeling	1.36E-08	2.71E-05	2.47E-06	2.02E-05	5	23	APOA2,APOA4,APOC1,APOC2,APOC3
GO: Biological Process	GO:0042157	lipoprotein metabolic process	1.63E-08	3.25E-05	2.70E-06	2.21E-05	8	131	PON1,APOA2,APOA4,APOC1,APOC2,ATM,APOC3,APOD
GO: Biological Process	GO:0034372	very-low-density lipoprotein particle remodeling	4.24E-08	8.44E-05	6.03E-06	4.93E-05	4	11	APOA2,APOA4,APOC2,APOC3
GO: Biological Process	GO:0034370	triglyceride-rich lipoprotein particle remodeling	4.24E-08	8.44E-05	6.03E-06	4.93E-05	4	11	APOA2,APOA4,APOC2,APOC3
GO: Biological Process	GO:0042060	wound healing	6.47E-08	1.29E-04	8.59E-06	7.02E-05	14	681	SAA1,SERPINA1,NF1,CENPE,FGA,FGB,FGG,APOD,F2,TTN,KLKB1,SERPIND1,GSN,CRP
GO: Biological Process	GO:0032372	negative regulation of sterol transport	9.13E-08	1.82E-04	1.04E-05	8.46E-05	4	13	APOA2,APOC1,APOC2,APOC3
GO: Biological Process	GO:0032375	negative regulation of cholesterol transport	9.13E-08	1.82E-04	1.04E-05	8.46E-05	4	13	APOA2,APOC1,APOC2,APOC3
GO: Biological Process	GO:0071827	plasma lipoprotein particle organization	9.35E-08	1.86E-04	1.04E-05	8.46E-05	5	33	APOA2,APOA4,APOC1,APOC2,APOC3
GO: Biological Process	GO:0010876	lipid localization	1.07E-07	2.13E-04	1.09E-05	8.90E-05	10	311	KIAA1109,PON1,APOA2,APOA4,APOC1,APOC2,APOC3,LBP,APOD,CRP
GO: Biological Process	GO:0015918	sterol transport	1.15E-07	2.29E-04	1.09E-05	8.90E-05	6	67	PON1,APOA2,APOA4,APOC1,APOC2,APOC3
GO: Biological Process	GO:0030301	cholesterol transport	1.15E-07	2.29E-04	1.09E-05	8.90E-05	6	67	PON1,APOA2,APOA4,APOC1,APOC2,APOC3
GO: Biological Process	GO:0034375	high-density lipoprotein particle remodeling	1.28E-07	2.54E-04	1.16E-05	9.44E-05	4	14	APOA2,APOA4,APOC1,APOC3
GO: Biological Process	GO:0006928	movement of cell or subcellular component	1.34E-07	2.66E-04	1.16E-05	9.46E-05	21	1703	SPTBN4,COL6A3,SAA1,MYH9,DNAH12,NF1,KIF27,DNAH10,MYO5A,CENPE,MYO7A,SPTAN1,ASPM,LAMA3,LBP,APOD,F2,DNAH5,DNAH8,TTN,DNAH11
GO: Biological Process	GO:0071825	protein-lipid complex subunit organization	1.47E-07	2.94E-04	1.17E-05	9.53E-05	5	36	APOA2,APOA4,APOC1,APOC2,APOC3
GO: Biological Process	GO:0034443	negative regulation of lipoprotein oxidation	1.58E-07	3.15E-04	1.17E-05	9.53E-05	3	4	PON1,APOA4,APOD
GO: Biological Process	GO:0010915	regulation of very-low-density lipoprotein particle	1.58E-07	3.15E-04	1.17E-05	9.53E-05	3	4	APOC1,APOC2,APOC3
GO: Biological Process	GO:0010916	negative regulation of very-low-density lipoprotein	1.58E-07	3.15E-04	1.17E-05	9.53E-05	3	4	APOC1,APOC2,APOC3
GO: Biological Process	GO:0046503	glycerolipid catabolic process	2.23E-07	4.45E-04	1.59E-05	1.30E-04	5	39	APOA2,APOA4,APOC1,APOC2,APOC3
GO: Biological Process	GO:0043691	reverse cholesterol transport	3.01E-07	5.99E-04	2.07E-05	1.69E-04	4	17	APOA2,APOA4,APOC2,APOC3
GO: Biological Process	GO:0016192	vesicle-mediated transport	3.58E-07	7.13E-04	2.38E-05	1.94E-04	17	1184	SPTBN4,SERPINA1,MYO5A,MYO5B,MYO7A,GCC2,SYNE1,FGA,FGB,APOC1,APOC2,APOC3,LBP,FGG,TTN,GSN,CRP
GO: Biological Process	GO:0034442	regulation of lipoprotein oxidation	3.94E-07	7.85E-04	2.45E-05	2.01E-04	3	5	PON1,APOA4,APOD
GO: Biological Process	GO:0010901	regulation of very-low-density lipoprotein particle	3.94E-07	7.85E-04	2.45E-05	2.01E-04	3	5	APOA2,APOC2,APOC3
GO: Biological Process	GO:0007017	microtubule-based process	4.08E-07	8.12E-04	2.46E-05	2.01E-04	12	559	MYH9,DNAH12,KIF27,DNAH10,SMC3,CENPE,GCC2,NUMA1,ASPM,DNAH5,DNAH8,DNAH11
GO: Biological Process	GO:0034377	plasma lipoprotein particle assembly	4.87E-07	9.71E-04	2.86E-05	2.33E-04	4	19	APOA2,APOA4,APOC1,APOC3
GO: Biological Process	GO:0050994	regulation of lipid catabolic process	5.82E-07	1.16E-03	3.31E-05	2.71E-04	5	47	APOA2,APOA4,APOC1,APOC2,APOC3
GO: Biological Process	GO:0032368	regulation of lipid transport	7.67E-07	1.53E-03	4.02E-05	3.28E-04	6	92	PON1,APOA2,APOA4,APOC1,APOC2,APOC3
GO: Biological Process	GO:0034447	very-low-density lipoprotein particle clearance	7.86E-07	1.57E-03	4.02E-05	3.28E-04	3	6	APOC1,APOC2,APOC3
GO: Biological Process	GO:0042160	lipoprotein modification	7.86E-07	1.57E-03	4.02E-05	3.28E-04	3	6	PON1,APOA4,APOD
GO: Biological Process	GO:0042161	lipoprotein oxidation	7.86E-07	1.57E-03	4.02E-05	3.28E-04	3	6	PON1,APOA4,APOD
GO: Biological Process	GO:0065005	protein-lipid complex assembly	9.13E-07	1.82E-03	4.55E-05	3.72E-04	4	22	APOA2,APOA4,APOC1,APOC3
GO: Biological Process	GO:0097006	regulation of plasma lipoprotein particle levels	1.18E-06	2.35E-03	5.73E-05	4.68E-04	5	54	APOA2,APOA4,APOC1,APOC2,APOC3
GO: Biological Process	GO:0010985	negative regulation of lipoprotein particle clearanc	1.37E-06	2.73E-03	6.08E-05	4.97E-04	3	7	APOC1,APOC2,APOC3
GO: Biological Process	GO:0071830	triglyceride-rich lipoprotein particle clearance	1.37E-06	2.73E-03	6.08E-05	4.97E-04	3	7	APOC1,APOC2,APOC3
GO: Biological Process	GO:0050748	negative regulation of lipoprotein metabolic proces	1.37E-06	2.73E-03	6.08E-05	4.97E-04	3	7	PON1,APOA4,APOD
GO: Biological Process	GO:0034382	chylomicron remnant clearance	1.37E-06	2.73E-03	6.08E-05	4.97E-04	3	7	APOC1,APOC2,APOC3
GO: Biological Process	GO:0051241	negative regulation of multicellular organismal pro	1.47E-06	2.93E-03	6.36E-05	5.20E-04	10	414	SPTBN4,ALMS1,APOA2,APOC1,APOC2,APOC3,LBP,APOD,F2,KLKB1
GO: Biological Process	GO:0010035	response to inorganic substance	1.50E-06	2.99E-03	6.36E-05	5.20E-04	10	415	SERPINA1,HP,FGA,FGB,PON1,APOA4,FGG,TTN,GSN,CRP
GO: Biological Process	GO:0015914	phospholipid transport	1.55E-06	3.08E-03	6.42E-05	5.25E-04	5	57	APOA2,APOA4,APOC1,APOC2,APOC3
GO: Biological Process	GO:0010873	positive regulation of cholesterol esterification	2.19E-06	4.36E-03	8.91E-05	7.28E-04	3	8	APOA2,APOA4,APOC1
GO: Biological Process	GO:0032369	negative regulation of lipid transport	2.52E-06	5.01E-03	9.63E-05	7.88E-04	4	28	APOA2,APOC1,APOC2,APOC3
GO: Biological Process	GO:0046464	acylglycerol catabolic process	2.52E-06	5.01E-03	9.63E-05	7.88E-04	4	28	APOA2,APOA4,APOC2,APOC3
GO: Biological Process	GO:0046461	neutral lipid catabolic process	2.52E-06	5.01E-03	9.63E-05	7.88E-04	4	28	APOA2,APOA4,APOC2,APOC3
GO: Biological Process	GO:0034384	high-density lipoprotein particle clearance	3.28E-06	6.53E-03	1.21E-04	9.88E-04	3	9	APOA2,APOC2,APOC3
GO: Biological Process	GO:0034116	positive regulation of heterotypic cell-cell adhesio	3.28E-06	6.53E-03	1.21E-04	9.88E-04	3	9	FGA,FGB,FGG
GO: Biological Process	GO:0045833	negative regulation of lipid metabolic process	3.48E-06	6.92E-03	1.26E-04	1.03E-03	5	67	APOA2,APOC1,APOC2,APOC3,APOD
GO: Biological Process	GO:0051128	regulation of cellular component organization	3.76E-06	7.49E-03	1.34E-04	1.09E-03	19	1733	SPTBN4,MYH9,ALMS1,CENPE,SYNE1,SPTAN1,FGA,KIAA1109,FGB,APOA2,APOC1,APOC2,ATM,APOC3,FGG,APOD,F2,GSN,CRP
GO: Biological Process	GO:0034381	plasma lipoprotein particle clearance	3.84E-06	7.64E-03	1.34E-04	1.10E-03	4	31	APOA2,APOC1,APOC2,APOC3
GO: Biological Process	GO:0042632	cholesterol homeostasis	4.32E-06	8.61E-03	1.43E-04	1.17E-03	5	70	ALMS1,APOA2,APOA4,APOC2,APOC3
GO: Biological Process	GO:0042304	regulation of fatty acid biosynthetic process	4.37E-06	8.71E-03	1.43E-04	1.17E-03	4	32	APOA4,APOC1,APOC2,APOC3
GO: Biological Process	GO:0043933	macromolecular complex subunit organization	4.42E-06	8.80E-03	1.43E-04	1.17E-03	19	1752	SPTBN4,MYH9,NF1,ALMS1,SMC3,CENPE,SPTAN1,ASPM,FGA,FGB,APOA2,APOA4,APOC1,APOC2,APOC3,FGG,TTN,GSN,CRP
GO: Biological Process	GO:0051258	protein polymerization	4.56E-06	9.09E-03	1.43E-04	1.17E-03	7	193	SPTBN4,SPTAN1,FGA,FGB,FGG,GSN,CRP
GO: Biological Process	GO:0008203	cholesterol metabolic process	4.62E-06	9.19E-03	1.43E-04	1.17E-03	6	125	SAA1,PON1,APOA2,APOA4,APOC1,APOC3
GO: Biological Process	GO:0055092	sterol homeostasis	4.64E-06	9.24E-03	1.43E-04	1.17E-03	5	71	ALMS1,APOA2,APOA4,APOC2,APOC3
GO: Biological Process	GO:0010872	regulation of cholesterol esterification	4.67E-06	9.30E-03	1.43E-04	1.17E-03	3	10	APOA2,APOA4,APOC1
GO: Biological Process	GO:0010896	regulation of triglyceride catabolic process	4.67E-06	9.30E-03	1.43E-04	1.17E-03	3	10	APOA4,APOC2,APOC3
GO: Biological Process	GO:0006869	lipid transport	4.93E-06	9.81E-03	1.49E-04	1.22E-03	8	277	PON1,APOA2,APOA4,APOC1,APOC2,APOC3,LBP,APOD
GO: Biological Process	GO:0007010	cytoskeleton organization	5.48E-06	1.09E-02	1.63E-04	1.33E-03	14	987	SPTBN4,MYH9,NF1,ALMS1,SMC3,CENPE,GCC2,SYNE1,SPTAN1,NUMA1,OBSCN,ASPM,TTN,GSN
GO: Biological Process	GO:0051004	regulation of lipoprotein lipase activity	5.61E-06	1.12E-02	1.64E-04	1.34E-03	4	34	APOA4,APOC1,APOC2,APOC3
GO: Biological Process	GO:0048468	cell development	6.23E-06	1.24E-02	1.80E-04	1.47E-03	20	1970	SPTBN4,COL6A3,MYH9,NF1,ALMS1,SMC3,MYO7A,SYNE1,SPTAN1,OBSCN,ASPM,FGA,FGB,ATM,FGG,APOD,F2,TTN,GSN,CRP
GO: Biological Process	GO:0007018	microtubule-based movement	6.57E-06	1.31E-02	1.87E-04	1.53E-03	7	204	DNAH12,KIF27,DNAH10,CENPE,DNAH5,DNAH8,DNAH11
GO: Biological Process	GO:0051129	negative regulation of cellular component organiza	7.18E-06	1.43E-02	2.02E-04	1.65E-03	10	495	SPTBN4,CENPE,SPTAN1,APOA2,APOC1,APOC2,ATM,APOC3,APOD,GSN
GO: Biological Process	GO:0016125	sterol metabolic process	8.16E-06	1.63E-02	2.26E-04	1.85E-03	6	138	SAA1,PON1,APOA2,APOA4,APOC1,APOC3
GO: Biological Process	GO:0050746	regulation of lipoprotein metabolic process	8.52E-06	1.70E-02	2.33E-04	1.90E-03	3	12	PON1,APOA4,APOD
GO: Biological Process	GO:0051235	maintenance of location	8.83E-06	1.76E-02	2.38E-04	1.94E-03	8	300	SPTBN4,CENPE,GCC2,SYNE1,ASPM,KIAA1109,F2,CRP
GO: Biological Process	GO:0051640	organelle localization	1.04E-05	2.08E-02	2.67E-04	2.18E-03	8	307	MYH9,MYO5A,CENPE,MYO7A,SYNE1,NUMA1,ASPM,KIAA1109
GO: Biological Process	GO:0051049	regulation of transport	1.06E-05	2.11E-02	2.67E-04	2.18E-03	17	1517	SPTBN4,SAA1,NF1,MYO5A,GCC2,SYNE1,FGA,FGB,PON1,APOA2,APOA4,APOC1,APOC2,APOC3,FGG,APOD,F2
GO: Biological Process	GO:0015748	organophosphate ester transport	1.06E-05	2.12E-02	2.67E-04	2.18E-03	5	84	APOA2,APOA4,APOC1,APOC2,APOC3
GO: Biological Process	GO:0034433	steroid esterification	1.11E-05	2.20E-02	2.67E-04	2.18E-03	3	13	APOA2,APOA4,APOC1
GO: Biological Process	GO:0010984	regulation of lipoprotein particle clearance	1.11E-05	2.20E-02	2.67E-04	2.18E-03	3	13	APOC1,APOC2,APOC3
GO: Biological Process	GO:0034435	cholesterol esterification	1.11E-05	2.20E-02	2.67E-04	2.18E-03	3	13	APOA2,APOA4,APOC1
GO: Biological Process	GO:0034434	sterol esterification	1.11E-05	2.20E-02	2.67E-04	2.18E-03	3	13	APOA2,APOA4,APOC1
GO: Biological Process	GO:0040011	locomotion	1.11E-05	2.21E-02	2.67E-04	2.18E-03	17	1522	SPTBN4,COL6A3,SAA1,MYH9,NF1,MYO5A,SPTAN1,ASPM,LAMA3,LBP,APOD,F2,DNAH5,DNAH8,TTN,SERPIND1,DNAH11
GO: Biological Process	GO:0043623	cellular protein complex assembly	1.12E-05	2.22E-02	2.67E-04	2.18E-03	9	410	SPTBN4,CENPE,SPTAN1,FGA,FGB,FGG,TTN,GSN,CRP
GO: Biological Process	GO:0002576	platelet degranulation	1.13E-05	2.24E-02	2.67E-04	2.18E-03	5	85	SERPINA1,FGA,FGB,FGG,TTN
GO: Biological Process	GO:2000910	negative regulation of sterol import	1.18E-05	2.36E-02	2.74E-04	2.24E-03	2	2	APOA2,APOC3
GO: Biological Process	GO:0060621	negative regulation of cholesterol import	1.18E-05	2.36E-02	2.74E-04	2.24E-03	2	2	APOA2,APOC3
GO: Biological Process	GO:0060627	regulation of vesicle-mediated transport	1.20E-05	2.39E-02	2.75E-04	2.25E-03	8	313	MYO5A,SYNE1,FGA,FGB,APOC1,APOC2,APOC3,FGG
GO: Biological Process	GO:0007596	blood coagulation	1.28E-05	2.55E-02	2.90E-04	2.37E-03	10	529	SAA1,SERPINA1,CENPE,FGA,FGB,FGG,F2,TTN,KLKB1,SERPIND1
GO: Biological Process	GO:0050817	coagulation	1.35E-05	2.68E-02	3.01E-04	2.46E-03	10	532	SAA1,SERPINA1,CENPE,FGA,FGB,FGG,F2,TTN,KLKB1,SERPIND1
GO: Biological Process	GO:0007599	hemostasis	1.39E-05	2.77E-02	3.05E-04	2.50E-03	10	534	SAA1,SERPINA1,CENPE,FGA,FGB,FGG,F2,TTN,KLKB1,SERPIND1
GO: Biological Process	GO:0048261	negative regulation of receptor-mediated endocyto	1.40E-05	2.79E-02	3.05E-04	2.50E-03	3	14	APOC1,APOC2,APOC3
GO: Biological Process	GO:0006200	ATP catabolic process	1.41E-05	2.81E-02	3.05E-04	2.50E-03	8	320	MYH9,DNAH12,DNAH10,MYO7A,RNF213,DNAH5,DNAH8,DNAH11
GO: Biological Process	GO:0030168	platelet activation	1.56E-05	3.11E-02	3.34E-04	2.73E-03	7	233	SAA1,SERPINA1,FGA,FGB,FGG,F2,TTN
GO: Biological Process	GO:0009158	ribonucleoside monophosphate catabolic process	1.61E-05	3.21E-02	3.38E-04	2.76E-03	8	326	MYH9,DNAH12,DNAH10,MYO7A,RNF213,DNAH5,DNAH8,DNAH11
GO: Biological Process	GO:0009169	purine ribonucleoside monophosphate catabolic pr	1.61E-05	3.21E-02	3.38E-04	2.76E-03	8	326	MYH9,DNAH12,DNAH10,MYO7A,RNF213,DNAH5,DNAH8,DNAH11
GO: Biological Process	GO:0009128	purine nucleoside monophosphate catabolic proces	1.65E-05	3.28E-02	3.40E-04	2.78E-03	8	327	MYH9,DNAH12,DNAH10,MYO7A,RNF213,DNAH5,DNAH8,DNAH11
GO: Biological Process	GO:0032989	cellular component morphogenesis	1.65E-05	3.30E-02	3.40E-04	2.78E-03	15	1242	SPTBN4,COL6A3,MYH9,KIF27,ALMS1,MYO7A,SYNE1,SPTAN1,OBSCN,FGA,FGB,FGG,F2,TTN,GSN
GO: Biological Process	GO:0009125	nucleoside monophosphate catabolic process	1.72E-05	3.43E-02	3.50E-04	2.86E-03	8	329	MYH9,DNAH12,DNAH10,MYO7A,RNF213,DNAH5,DNAH8,DNAH11
GO: Biological Process	GO:0060192	negative regulation of lipase activity	1.75E-05	3.48E-02	3.52E-04	2.88E-03	3	15	APOA2,APOC1,APOC3
GO: Biological Process	GO:1902041	regulation of extrinsic apoptotic signaling pathway	1.91E-05	3.81E-02	3.81E-04	3.12E-03	4	46	NF1,FGA,FGB,FGG
GO: Biological Process	GO:0032880	regulation of protein localization	1.94E-05	3.86E-02	3.82E-04	3.12E-03	10	555	SAA1,NF1,MYO5B,CENPE,GCC2,FGA,FGB,APOA2,FGG,APOD
GO: Biological Process	GO:0030155	regulation of cell adhesion	2.42E-05	4.82E-02	4.73E-04	3.87E-03	8	345	SAA1,NF1,FGA,FGB,LAMA3,FGG,APOD,GSN
GO: Biological Process	GO:0034114	regulation of heterotypic cell-cell adhesion	2.60E-05	5.18E-02	5.03E-04	4.11E-03	3	17	FGA,FGB,FGG
GO: Biological Process	GO:0051592	response to calcium ion	3.00E-05	5.98E-02	5.69E-04	4.65E-03	5	104	FGA,FGB,FGG,TTN,CRP
GO: Biological Process	GO:0055088	lipid homeostasis	3.00E-05	5.98E-02	5.69E-04	4.65E-03	5	104	ALMS1,APOA2,APOA4,APOC2,APOC3
GO: Biological Process	GO:0030029	actin filament-based process	3.36E-05	6.69E-02	6.31E-04	5.16E-03	10	592	SPTBN4,MYH9,NF1,ALMS1,MYO5A,MYO7A,SPTAN1,OBSCN,TTN,GSN
GO: Biological Process	GO:2000909	regulation of sterol import	3.53E-05	7.04E-02	6.34E-04	5.18E-03	2	3	APOA2,APOC3
GO: Biological Process	GO:0034444	regulation of plasma lipoprotein particle oxidation	3.53E-05	7.04E-02	6.34E-04	5.18E-03	2	3	PON1,APOA4
GO: Biological Process	GO:0060620	regulation of cholesterol import	3.53E-05	7.04E-02	6.34E-04	5.18E-03	2	3	APOA2,APOC3
GO: Biological Process	GO:0034445	negative regulation of plasma lipoprotein particle c	3.53E-05	7.04E-02	6.34E-04	5.18E-03	2	3	PON1,APOA4
GO: Biological Process	GO:0010903	negative regulation of very-low-density lipoprotein	3.53E-05	7.04E-02	6.34E-04	5.18E-03	2	3	APOA2,APOC3
GO: Biological Process	GO:0019216	regulation of lipid metabolic process	3.74E-05	7.44E-02	6.64E-04	5.43E-03	7	267	SERPINA3,APOA2,APOA4,APOC1,APOC2,APOC3,APOD
GO: Biological Process	GO:2000352	negative regulation of endothelial cell apoptotic pr	4.33E-05	8.62E-02	7.56E-04	6.18E-03	3	20	FGA,FGB,FGG
GO: Biological Process	GO:1900026	positive regulation of substrate adhesion-depende	4.33E-05	8.62E-02	7.56E-04	6.18E-03	3	20	FGA,FGB,FGG
GO: Biological Process	GO:0006641	triglyceride metabolic process	4.86E-05	9.69E-02	8.42E-04	6.89E-03	5	115	APOA2,APOA4,APOC1,APOC2,APOC3
GO: Biological Process	GO:0050996	positive regulation of lipid catabolic process	5.04E-05	1.00E-01	8.52E-04	6.97E-03	3	21	APOA2,APOA4,APOC2
GO: Biological Process	GO:0050995	negative regulation of lipid catabolic process	5.04E-05	1.00E-01	8.52E-04	6.97E-03	3	21	APOA2,APOC1,APOC3
GO: Biological Process	GO:0010038	response to metal ion	5.05E-05	1.01E-01	8.52E-04	6.97E-03	7	280	SERPINA1,FGA,FGB,FGG,TTN,GSN,CRP
GO: Biological Process	GO:0030198	extracellular matrix organization	5.27E-05	1.05E-01	8.82E-04	7.21E-03	8	385	COL6A3,NF1,FGA,FGB,LAMA3,FGG,PLEC,KLKB1
GO: Biological Process	GO:0043062	extracellular structure organization	5.37E-05	1.07E-01	8.91E-04	7.28E-03	8	386	COL6A3,NF1,FGA,FGB,LAMA3,FGG,PLEC,KLKB1
GO: Biological Process	GO:0015850	organic hydroxy compound transport	5.76E-05	1.15E-01	9.41E-04	7.69E-03	6	195	PON1,APOA2,APOA4,APOC1,APOC2,APOC3
GO: Biological Process	GO:0046434	organophosphate catabolic process	5.76E-05	1.15E-01	9.41E-04	7.69E-03	14	1218	MYH9,DNAH12,NF1,DNAH10,MYO7A,OBSCN,PON1,RNF213,APOA2,APOC1,APOC2,DNAH5,DNAH8,DNAH11
GO: Biological Process	GO:0043086	negative regulation of catalytic activity	6.11E-05	1.22E-01	9.89E-04	8.08E-03	11	771	SERPINA3,COL6A3,SERPINA1,NF1,HP,APOA2,APOC1,APOC2,APOC3,SERPIND1,HEXIM1
GO: Biological Process	GO:0045184	establishment of protein localization	6.41E-05	1.28E-01	1.03E-03	8.42E-03	16	1567	SPTBN4,SAA1,MYH9,NF1,MYO5A,MYO5B,CENPE,MYO7A,GCC2,FGA,FGB,APOA2,APOC2,APOC3,FGG,APOD
GO: Biological Process	GO:0072378	blood coagulation, fibrin clot formation	6.68E-05	1.33E-01	1.06E-03	8.66E-03	3	23	FGA,F2,KLKB1
GO: Biological Process	GO:0006639	acylglycerol metabolic process	6.70E-05	1.34E-01	1.06E-03	8.66E-03	5	123	APOA2,APOA4,APOC1,APOC2,APOC3
GO: Biological Process	GO:0006638	neutral lipid metabolic process	6.97E-05	1.39E-01	1.07E-03	8.76E-03	5	124	APOA2,APOA4,APOC1,APOC2,APOC3
GO: Biological Process	GO:0060191	regulation of lipase activity	6.97E-05	1.39E-01	1.07E-03	8.76E-03	5	124	APOA2,APOA4,APOC1,APOC2,APOC3
GO: Biological Process	GO:0051651	maintenance of location in cell	6.97E-05	1.39E-01	1.07E-03	8.76E-03	5	124	SPTBN4,CENPE,GCC2,SYNE1,ASPM
GO: Biological Process	GO:0034371	chylomicron remodeling	7.05E-05	1.41E-01	1.07E-03	8.76E-03	2	4	APOA4,APOC2
GO: Biological Process	GO:0034441	plasma lipoprotein particle oxidation	7.05E-05	1.41E-01	1.07E-03	8.76E-03	2	4	PON1,APOA4
GO: Biological Process	GO:0051223	regulation of protein transport	7.38E-05	1.47E-01	1.11E-03	9.11E-03	8	404	SAA1,NF1,GCC2,FGA,FGB,APOA2,FGG,APOD
GO: Biological Process	GO:0044712	single-organism catabolic process	7.49E-05	1.49E-01	1.12E-03	9.18E-03	18	1950	COL6A3,MYH9,DNAH12,NF1,DNAH10,MYO7A,HP,OBSCN,PON1,RNF213,APOA2,APOA4,APOC1,APOC2,APOC3,DNAH5,DNAH8,DNAH11
GO: Biological Process	GO:0048259	regulation of receptor-mediated endocytosis	7.56E-05	1.51E-01	1.12E-03	9.18E-03	4	65	SYNE1,APOC1,APOC2,APOC3
GO: Biological Process	GO:0001539	cilium or flagellum-dependent cell motility	7.61E-05	1.52E-01	1.12E-03	9.18E-03	3	24	DNAH5,DNAH8,DNAH11
GO: Biological Process	GO:0008104	protein localization	7.85E-05	1.56E-01	1.15E-03	9.40E-03	18	1957	SPTBN4,SAA1,MYH9,NF1,MYO5A,MYO5B,CENPE,MYO7A,GCC2,SYNE1,OBSCN,FGA,FGB,APOA2,APOC2,APOC3,FGG,APOD
GO: Cellular Component	GO:0005615	extracellular space	1.62E-16	4.22E-14	2.83E-14	1.74E-13	28	1284	SERPINA3,ORM1,ORM2,COL6A3,SAA1,DMXL2,SERPINA1,SAA2,HP,HPR,FGA,FGB,PON1,APOA2,APOA4,APOC1,APOC2,APOC3,CPN1,LB P,FGG,APOD,F2,KLKB1,LRG1,SERPIND1,GSN,CRP
GO: Cellular Component	GO:0034364	high-density lipoprotein particle	2.17E-16	5.65E-14	2.83E-14	1.74E-13	9	28	SAA1,SAA2,HPR,PON1,APOA2,APOA4,APOC1,APOC2,APOC3
GO: Cellular Component	GO:0072562	blood microparticle	5.12E-16	1.33E-13	4.43E-14	2.72E-13	13	132	SERPINA3,ORM1,ORM2,HP,HPR,FGA,FGB,PON1,APOA2,APOA4,FGG,F2,GSN
GO: Cellular Component	GO:0034358	plasma lipoprotein particle	8.34E-15	2.17E-12	5.42E-13	3.33E-12	9	40	SAA1,SAA2,HPR,PON1,APOA2,APOA4,APOC1,APOC2,APOC3
GO: Cellular Component	GO:0032994	protein-lipid complex	1.35E-14	3.52E-12	7.03E-13	4.32E-12	9	42	SAA1,SAA2,HPR,PON1,APOA2,APOA4,APOC1,APOC2,APOC3
GO: Cellular Component	GO:0034366	spherical high-density lipoprotein particle	2.13E-11	5.54E-09	9.23E-10	5.66E-09	5	8	HPR,PON1,APOA2,APOC2,APOC3
GO: Cellular Component	GO:0044430	cytoskeletal part	1.94E-10	5.05E-08	7.21E-09	4.43E-08	23	1477	SPTBN4,MYH9,DNAH12,NF1,KIF27,MYH13,ALMS1,DNAH10,MYO5A,MYO5B,SMC3,CENPE,MYO7A,SYNE1,SPTAN1,NUMA1,OBSCN,ASPM ,ATM,DNAH5,DNAH8,TTN,DNAH11
GO: Cellular Component	GO:0042627	chylomicron	2.98E-10	7.75E-08	9.68E-09	5.95E-08	5	12	APOA2,APOA4,APOC1,APOC2,APOC3
GO: Cellular Component	GO:0005856	cytoskeleton	3.90E-10	1.01E-07	1.13E-08	6.92E-08	26	1993	SPTBN4,MYH9,DNAH12,NF1,KIF27,MYH13,ALMS1,DNAH10,MYO5A,MYO5B,SMC3,CENPE,MYO7A,SYNE1,SPTAN1,NUMA1,OBSCN,ASPM ,EVPL,ATM,PLEC,DNAH5,DNAH8,TTN,GSN,DNAH11
GO: Cellular Component	GO:0016459	myosin complex	2.20E-09	5.72E-07	5.72E-08	3.51E-07	7	65	MYH9,MYH13,MYO5A,MYO5B,MYO7A,OBSCN,TTN
GO: Cellular Component	GO:0034385	triglyceride-rich lipoprotein particle	5.71E-09	1.49E-06	1.24E-07	7.60E-07	5	20	APOA2,APOA4,APOC1,APOC2,APOC3
GO: Cellular Component	GO:0034361	very-low-density lipoprotein particle	5.71E-09	1.49E-06	1.24E-07	7.60E-07	5	20	APOA2,APOA4,APOC1,APOC2,APOC3
GO: Cellular Component	GO:0015630	microtubule cytoskeleton	1.02E-07	2.65E-05	2.04E-06	1.25E-05	16	971	MYH9,DNAH12,KIF27,ALMS1,DNAH10,MYO5A,SMC3,CENPE,SYNE1,SPTAN1,NUMA1,ASPM,ATM,DNAH5,DNAH8,DNAH11
GO: Cellular Component	GO:0005858	axonemal dynein complex	2.13E-07	5.53E-05	3.95E-06	2.42E-05	4	16	DNAH10,DNAH5,DNAH8,DNAH11
GO: Cellular Component	GO:0030286	dynein complex	2.61E-07	6.80E-05	4.47E-06	2.74E-05	5	41	DNAH12,DNAH10,DNAH5,DNAH8,DNAH11
GO: Cellular Component	GO:0060205	cytoplasmic membrane-bounded vesicle lumen	2.75E-07	7.14E-05	4.47E-06	2.74E-05	6	79	SAA1,SERPINA1,HP,FGA,FGB,FGG
GO: Cellular Component	GO:0031983	vesicle lumen	3.43E-07	8.93E-05	5.25E-06	3.23E-05	6	82	SAA1,SERPINA1,HP,FGA,FGB,FGG
GO: Cellular Component	GO:0005875	microtubule associated complex	4.22E-07	1.10E-04	6.10E-06	3.74E-05	7	138	DNAH12,KIF27,DNAH10,CENPE,DNAH5,DNAH8,DNAH11
GO: Cellular Component	GO:0042995	cell projection	5.02E-07	1.31E-04	6.87E-06	4.22E-05	20	1711	SPTBN4,FSIP2,MYH9,DNAH12,NF1,KIF27,ALMS1,DNAH10,MYO5A,MYO5B,MYO7A,SYNE1,SPTAN1,NUMA1,APOD,DNAH5,DNAH8,GSN,CRP,DNAH11
GO: Cellular Component	GO:0005874	microtubule	6.80E-07	1.77E-04	8.84E-06	5.43E-05	10	388	DNAH12,KIF27,DNAH10,MYO5A,CENPE,NUMA1,ASPM,DNAH5,DNAH8,DNAH11
GO: Cellular Component	GO:0015629	actin cytoskeleton	1.25E-06	3.25E-04	1.55E-05	9.50E-05	10	415	SPTBN4,MYH9,MYH13,MYO5A,MYO5B,MYO7A,SPTAN1,OBSCN,TTN,GSN
GO: Cellular Component	GO:0005929	cilium	1.39E-06	3.62E-04	1.65E-05	1.01E-04	10	420	FSIP2,DNAH12,KIF27,ALMS1,DNAH10,MYO5A,MYO7A,DNAH5,DNAH8,DNAH11
GO: Cellular Component	GO:0016460	myosin II complex	1.99E-06	5.18E-04	2.23E-05	1.37E-04	4	27	MYH9,MYH13,OBSCN,TTN
GO: Cellular Component	GO:0005577	fibrinogen complex	2.06E-06	5.36E-04	2.23E-05	1.37E-04	3	8	FGA,FGB,FGG
GO: Cellular Component	GO:0044447	axoneme part	3.09E-06	8.03E-04	3.09E-05	1.90E-04	4	30	DNAH10,DNAH5,DNAH8,DNAH11
GO: Cellular Component	GO:0005868	cytoplasmic dynein complex	3.09E-06	8.03E-04	3.09E-05	1.90E-04	4	30	DNAH10,DNAH5,DNAH8,DNAH11
GO: Cellular Component	GO:0005819	spindle	3.51E-06	9.13E-04	3.38E-05	2.08E-04	8	270	MYH9,ALMS1,SMC3,CENPE,SYNE1,NUMA1,ASPM,ATM
GO: Cellular Component	GO:0005938	cell cortex	1.26E-05	3.27E-03	1.17E-04	7.17E-04	7	230	SPTBN4,MYH9,MYO7A,SPTAN1,FGA,FGB,FGG
GO: Cellular Component	GO:0031093	platelet alpha granule lumen	2.10E-05	5.45E-03	1.88E-04	1.15E-03	4	48	SERPINA1,FGA,FGB,FGG
GO: Cellular Component	GO:0005859	muscle myosin complex	4.07E-05	1.06E-02	3.53E-04	2.17E-03	3	20	MYH13,OBSCN,TTN
GO: Cellular Component	GO:0044449	contractile fiber part	4.86E-05	1.26E-02	4.07E-04	2.50E-03	6	193	MYH13,SYNE1,SPTAN1,OBSCN,PLEC,TTN
GO: Cellular Component	GO:0031091	platelet alpha granule	5.44E-05	1.41E-02	4.31E-04	2.64E-03	4	61	SERPINA1,FGA,FGB,FGG
GO: Cellular Component	GO:0032982	myosin filament	5.48E-05	1.42E-02	4.31E-04	2.64E-03	3	22	MYH9,MYH13,OBSCN
GO: Cellular Component	GO:0030016	myofibril	5.76E-05	1.50E-02	4.31E-04	2.64E-03	6	199	MYH13,SYNE1,SPTAN1,OBSCN,PLEC,TTN
GO: Cellular Component	GO:0034774	secretory granule lumen	5.80E-05	1.51E-02	4.31E-04	2.64E-03	4	62	SERPINA1,FGA,FGB,FGG
GO: Cellular Component	GO:0034363	intermediate-density lipoprotein particle	6.77E-05	1.76E-02	4.89E-04	3.00E-03	2	4	APOC2,APOC3
GO: Cellular Component	GO:0043292	contractile fiber	8.17E-05	2.13E-02	5.74E-04	3.53E-03	6	212	MYH13,SYNE1,SPTAN1,OBSCN,PLEC,TTN
GO: Cellular Component	GO:0044463	cell projection part	1.08E-04	2.81E-02	7.40E-04	4.55E-03	11	839	SPTBN4,ALMS1,DNAH10,MYO5A,MYO5B,SYNE1,SPTAN1,DNAH5,DNAH8,CRP,DNAH11
GO: Cellular Component	GO:0070161	anchoring junction	1.37E-04	3.57E-02	9.15E-04	5.62E-03	6	233	SPTBN4,MYH9,SPTAN1,LAMA3,EVPL,PLEC
GO: Cellular Component	GO:0097014	ciliary cytoplasm	1.50E-04	3.89E-02	9.48E-04	5.82E-03	4	79	DNAH10,DNAH5,DNAH8,DNAH11
GO: Cellular Component	GO:0005930	axoneme	1.50E-04	3.89E-02	9.48E-04	5.82E-03	4	79	DNAH10,DNAH5,DNAH8,DNAH11

Table S2.2 Non-survivors patients D0 samples.

Category	ID	Name	p-value	q-value Bonferroni	q-value FDR B&H	q-value FDR B&Y	Hit Count in Query List	Hit Count in Genome	Hit in Query List
GO: Molecular Function	GO:0004857	enzyme inhibitor activity	3.14E-09	7.40E-07	7.40E-07	4.47E-06	12	344	SERPINA3,COL6A3,SERPINA1,SBF1,SERPING1,APOA2,APOC1,APOC2,APOC3,SERPIND1,HEXIM1,PROS1
GO: Molecular Function	GO:0003774	motor activity	2.10E-08	4.95E-06	2.48E-06	1.50E-05	8	130	MYH9,DNAH12,KIF27,DNAH10,MYO5B,DNAH5,DNAH8,DNAH11
GO: Molecular Function	GO:0050997	quaternary ammonium group binding	2.07E-07	4.89E-05	1.52E-05	9.21E-05	5	37	NF1,APOA2,APOA4,APOC1,CRP
GO: Molecular Function	GO:0003777	microtubule motor activity	3.36E-07	7.92E-05	1.52E-05	9.21E-05	6	77	DNAH12,KIF27,DNAH10,DNAH5,DNAH8,DNAH11
GO: Molecular Function	GO:0031210	phosphatidylcholine binding	3.53E-07	8.32E-05	1.52E-05	9.21E-05	4	17	NF1,APOA2,APOA4,APOC1
GO: Molecular Function	GO:0060228	phosphatidylcholine-sterol O-acyltransferase activator activity	4.44E-07	1.05E-04	1.52E-05	9.21E-05	3	5	APOA2,APOA4,APOC1
GO: Molecular Function	GO:0055102	lipase inhibitor activity	4.52E-07	1.07E-04	1.52E-05	9.21E-05	4	18	APOA2,APOC1,APOC2,APOC3
GO: Molecular Function	GO:0043178	alcohol binding	1.68E-06	3.96E-04	4.95E-05	2.99E-04	6	101	NF1,APOA2,APOA4,APOC1,APOC3,CRP
GO: Molecular Function	GO:0030492	hemoglobin binding	2.47E-06	5.82E-04	6.47E-05	3.91E-04	3	8	HP,HPR,HBB
GO: Molecular Function	GO:0030234	enzyme regulator activity	3.43E-06	8.09E-04	8.09E-05	4.89E-04	14	909	SERPINA3,COL6A3,SERPINA1,NF1,SBF1,SERPING1,APOA2,APOA4,APOC1,APOC2,APOC3,SERPIND1,HEXIM1,PROS1
GO: Molecular Function	GO:0008092	cytoskeletal protein binding	4.07E-06	9.61E-04	8.74E-05	5.28E-04	13	792	SHROOM3,MYH9,DST,NF1,KIF27,ANK3,ALMS1,MYO5B,SPTAN1,SYNE2,ACTB,NUMA1,GSN
GO: Molecular Function	GO:0004867	serine-type endopeptidase inhibitor activity	2.47E-05	5.82E-03	4.85E-04	2.93E-03	5	96	SERPINA3,COL6A3,SERPINA1,SERPING1,SERPIND1
GO: Molecular Function	GO:0004866	endopeptidase inhibitor activity	2.83E-05	6.67E-03	5.13E-04	3.10E-03	6	165	SERPINA3,COL6A3,SERPINA1,SERPING1,SERPIND1,PROS1
GO: Molecular Function	GO:0061135	endopeptidase regulator activity	3.46E-05	8.16E-03	5.31E-04	3.21E-03	6	171	SERPINA3,COL6A3,SERPINA1,SERPING1,SERPIND1,PROS1
GO: Molecular Function	GO:0030414	peptidase inhibitor activity	3.57E-05	8.43E-03	5.31E-04	3.21E-03	6	172	SERPINA3,COL6A3,SERPINA1,SERPING1,SERPIND1,PROS1
GO: Molecular Function	GO:0070653	high-density lipoprotein particle receptor binding	3.82E-05	9.02E-03	5.31E-04	3.21E-03	2	3	APOA2,APOC3
GO: Molecular Function	GO:0031720	haptoglobin binding	3.82E-05	9.02E-03	5.31E-04	3.21E-03	2	3	HBA1,HBB
GO: Molecular Function	GO:0008289	lipid binding	6.89E-05	1.63E-02	9.03E-04	5.46E-03	10	619	NF1,PON1,APOA2,APOA4,APOC1,APOC2,APOC3,LBP,DYSF,CRP
GO: Molecular Function	GO:0016887	ATPase activity	1.03E-04	2.43E-02	1.23E-03	7.43E-03	8	407	MYH9,DNAH12,MDN1,KIF27,DNAH10,DNAH5,DNAH8,DNAH11
GO: Molecular Function	GO:0071813	lipoprotein particle binding	1.09E-04	2.58E-02	1.23E-03	7.43E-03	3	26	PON1,APOA2,CRP
GO: Molecular Function	GO:0071814	protein-lipid complex binding	1.09E-04	2.58E-02	1.23E-03	7.43E-03	3	26	PON1,APOA2,CRP
GO: Molecular Function	GO:0061134	peptidase regulator activity	1.17E-04	2.76E-02	1.25E-03	7.57E-03	6	213	SERPINA3,COL6A3,SERPINA1,SERPING1,SERPIND1,PROS1
GO: Molecular Function	GO:0005543	phospholipid binding	1.25E-04	2.96E-02	1.29E-03	7.76E-03	7	311	NF1,PON1,APOA2,APOA4,APOC1,APOC3,DYSF
GO: Molecular Function	GO:0016209	antioxidant activity	1.31E-04	3.10E-02	1.29E-03	7.80E-03	4	72	HP,APOA4,HBA1,HBB
GO: Molecular Function	GO:0030674	protein binding, bridging	1.49E-04	3.51E-02	1.41E-03	8.49E-03	5	140	ANK3,FGA,FGB,EVPL,FGG
GO: Molecular Function	GO:0005509	calcium ion binding	1.76E-04	4.16E-02	1.60E-03	9.67E-03	10	694	DST,SPTAN1,PON1,C1R,F2,DYSF,SVEP1,GSN,CRP,PROS1
GO: Biological Process	GO:0006953	acute-phase response	1.67E-15	3.00E-12	3.00E-12	2.42E-11	10	49	SERPINA3,ORM1,ORM2,SAA1,SERPINA1,SAA2,HP,LBP,F2,CRP
GO: Biological Process	GO:0002526	acute inflammatory response	3.37E-15	6.05E-12	3.02E-12	2.44E-11	13	140	SERPINA3,ORM1,ORM2,SAA1,SERPINA1,SAA2,HP,SERPING1,APOA2,LBP,F2,CRP,PROS1
GO: Biological Process	GO:0009611	response to wounding	7.72E-13	1.38E-09	4.61E-10	3.72E-09	25	1255	SERPINA3,ORM1,ORM2,SAA1,SERPINA1,SAA2,DST,NF1,HP,ACTB,FGA,FGB,SERPING1,APOA2,APOC3,LBP,FGG,F2,HBB,DYSF,HBD,SERPIND1,GSN,CRP,PROS1
GO: Biological Process	GO:0033700	phospholipid efflux	4.51E-10	8.08E-07	2.02E-07	1.63E-06	5	12	APOA2,APOA4,APOC1,APOC2,APOC3
GO: Biological Process	GO:0042060	wound healing	2.39E-09	4.29E-06	7.57E-07	6.11E-06	16	681	SAA1,SERPINA1,NF1,ACTB,FGA,FGB,SERPING1,FGG,F2,HBB,DYSF,HBD,SERPIND1,GSN,CRP,PROS1
GO: Biological Process	GO:0032374	regulation of cholesterol transport	2.96E-09	5.30E-06	7.57E-07	6.11E-06	6	35	PON1,APOA2,APOA4,APOC1,APOC2,APOC3
GO: Biological Process	GO:0032371	regulation of sterol transport	2.96E-09	5.30E-06	7.57E-07	6.11E-06	6	35	PON1,APOA2,APOA4,APOC1,APOC2,APOC3
GO: Biological Process	GO:0033344	cholesterol efflux	6.89E-09	1.24E-05	1.54E-06	1.25E-05	6	40	PON1,APOA2,APOA4,APOC1,APOC2,APOC3
GO: Biological Process	GO:0034369	plasma lipoprotein particle remodeling	1.86E-08	3.33E-05	3.03E-06	2.44E-05	5	23	APOA2,APOA4,APOC1,APOC2,APOC3
GO: Biological Process	GO:0034368	protein-lipid complex remodeling	1.86E-08	3.33E-05	3.03E-06	2.44E-05	5	23	APOA2,APOA4,APOC1,APOC2,APOC3
GO: Biological Process	GO:0034367	macromolecular complex remodeling	1.86E-08	3.33E-05	3.03E-06	2.44E-05	5	23	APOA2,APOA4,APOC1,APOC2,APOC3
GO: Biological Process	GO:0006954	inflammatory response	2.98E-08	5.34E-05	4.45E-06	3.59E-05	14	599	SERPINA3,ORM1,ORM2,SAA1,SERPINA1,SAA2,HP,SERPING1,APOA2,APOC3,LBP,F2,CRP,PROS1
GO: Biological Process	GO:0034372	very-low-density lipoprotein particle remodeling	5.42E-08	9.73E-05	6.95E-06	5.61E-05	4	11	APOA2,APOA4,APOC2,APOC3
GO: Biological Process	GO:0034370	triglyceride-rich lipoprotein particle remodeling	5.42E-08	9.73E-05	6.95E-06	5.61E-05	4	11	APOA2,APOA4,APOC2,APOC3
GO: Biological Process	GO:0032372	negative regulation of sterol transport	1.17E-07	2.10E-04	1.31E-05	1.06E-04	4	13	APOA2,APOC1,APOC2,APOC3
GO: Biological Process	GO:0032375	negative regulation of cholesterol transport	1.17E-07	2.10E-04	1.31E-05	1.06E-04	4	13	APOA2,APOC1,APOC2,APOC3
GO: Biological Process	GO:0071827	plasma lipoprotein particle organization	1.27E-07	2.28E-04	1.34E-05	1.08E-04	5	33	APOA2,APOA4,APOC1,APOC2,APOC3
GO: Biological Process	GO:0034375	high-density lipoprotein particle remodeling	1.63E-07	2.93E-04	1.49E-05	1.20E-04	4	14	APOA2,APOA4,APOC1,APOC3
GO: Biological Process	GO:0015918	sterol transport	1.66E-07	2.97E-04	1.49E-05	1.20E-04	6	67	PON1,APOA2,APOA4,APOC1,APOC2,APOC3
GO: Biological Process	GO:0030301	cholesterol transport	1.66E-07	2.97E-04	1.49E-05	1.20E-04	6	67	PON1,APOA2,APOA4,APOC1,APOC2,APOC3
GO: Biological Process	GO:0010915	regulation of very-low-density lipoprotein particle clearance	1.90E-07	3.41E-04	1.55E-05	1.25E-04	3	4	APOC1,APOC2,APOC3
GO: Biological Process	GO:0010916	negative regulation of very-low-density lipoprotein particle clearance	1.90E-07	3.41E-04	1.55E-05	1.25E-04	3	4	APOC1,APOC2,APOC3
GO: Biological Process	GO:0071825	protein-lipid complex subunit organization	2.00E-07	3.59E-04	1.56E-05	1.26E-04	5	36	APOA2,APOA4,APOC1,APOC2,APOC3
GO: Biological Process	GO:0046503	glycerolipid catabolic process	3.03E-07	5.44E-04	2.27E-05	1.83E-04	5	39	APOA2,APOA4,APOC1,APOC2,APOC3
GO: Biological Process	GO:0043691	reverse cholesterol transport	3.85E-07	6.90E-04	2.76E-05	2.23E-04	4	17	APOA2,APOA4,APOC2,APOC3
GO: Biological Process	GO:0006928	movement of cell or subcellular component	4.26E-07	7.63E-04	2.94E-05	2.37E-04	21	1703	COL6A3,SAA1,MYH9,DNAH12,DST,NF1,KIF27,ANK3,DNAH10,SPTAN1,SYNE2,ACTB,ASPM,LAMA3,LBP,F2,DNAH5,DNAH8,PROS1,PCNT,DNAH11
GO: Biological Process	GO:0007596	blood coagulation	4.52E-07	8.11E-04	2.97E-05	2.40E-04	12	529	SAA1,SERPINA1,ACTB,FGA,FGB,SERPING1,FGG,F2,HBB,HBD,SERPIND1,PROS1
GO: Biological Process	GO:0010901	regulation of very-low-density lipoprotein particle remodeling	4.74E-07	8.50E-04	2.97E-05	2.40E-04	3	5	APOA2,APOC2,APOC3
GO: Biological Process	GO:0050817	coagulation	4.80E-07	8.61E-04	2.97E-05	2.40E-04	12	532	SAA1,SERPINA1,ACTB,FGA,FGB,SERPING1,FGG,F2,HBB,HBD,SERPIND1,PROS1
GO: Biological Process	GO:0007599	hemostasis	5.00E-07	8.96E-04	2.99E-05	2.41E-04	12	534	SAA1,SERPINA1,ACTB,FGA,FGB,SERPING1,FGG,F2,HBB,HBD,SERPIND1,PROS1
GO: Biological Process	GO:0034377	plasma lipoprotein particle assembly	6.23E-07	1.12E-03	3.61E-05	2.91E-04	4	19	APOA2,APOA4,APOC1,APOC3
GO: Biological Process	GO:0043086	negative regulation of catalytic activity	6.52E-07	1.17E-03	3.66E-05	2.95E-04	14	771	SERPINA3,COL6A3,SERPINA1,NF1,SBF1,HP,SERPING1,APOA2,APOC1,APOC2,APOC3,SERPIND1,HEXIM1,PROS1
GO: Biological Process	GO:0002576	platelet degranulation	6.89E-07	1.24E-03	3.75E-05	3.02E-04	6	85	SERPINA1,FGA,FGB,SERPING1,FGG,PROS1
GO: Biological Process	GO:0050994	regulation of lipid catabolic process	7.90E-07	1.42E-03	4.15E-05	3.35E-04	5	47	APOA2,APOA4,APOC1,APOC2,APOC3
GO: Biological Process	GO:0007017	microtubule-based process	8.10E-07	1.45E-03	4.15E-05	3.35E-04	12	559	MYH9,DNAH12,DST,KIF27,DNAH10,GCC2,NUMA1,ASPM,DNAH5,DNAH8,PCNT,DNAH11
GO: Biological Process	GO:0016192	vesicle-mediated transport	9.14E-07	1.64E-03	4.55E-05	3.67E-04	17	1184	SERPINA1,ANK3,MYO5B,GCC2,ACTB,FGA,FGB,SERPING1,APOC1,APOC2,APOC3,LBP,FGG,DYSF,GSN,CRP,PROS1
GO: Biological Process	GO:0034447	very-low-density lipoprotein particle clearance	9.45E-07	1.70E-03	4.58E-05	3.70E-04	3	6	APOC1,APOC2,APOC3
GO: Biological Process	GO:0072376	protein activation cascade	1.03E-06	1.85E-03	4.88E-05	3.93E-04	6	91	FGA,SERPING1,C1R,F2,CRP,PROS1
GO: Biological Process	GO:0032368	regulation of lipid transport	1.10E-06	1.98E-03	5.07E-05	4.09E-04	6	92	PON1,APOA2,APOA4,APOC1,APOC2,APOC3
GO: Biological Process	GO:0065005	protein-lipid complex assembly	1.17E-06	2.09E-03	5.23E-05	4.22E-04	4	22	APOA2,APOA4,APOC1,APOC3
GO: Biological Process	GO:0040011	locomotion	1.49E-06	2.67E-03	6.52E-05	5.26E-04	19	1522	COL6A3,SAA1,MYH9,DST,NF1,ANK3,SPTAN1,SYNE2,ACTB,ASPM,LAMA3,LBP,F2,DNAH5,DNAH8,SERPIND1,PROS1,PCNT,DNAH11
GO: Biological Process	GO:0097006	regulation of plasma lipoprotein particle levels	1.60E-06	2.87E-03	6.58E-05	5.31E-04	5	54	APOA2,APOA4,APOC1,APOC2,APOC3
GO: Biological Process	GO:0010985	negative regulation of lipoprotein particle clearance	1.65E-06	2.96E-03	6.58E-05	5.31E-04	3	7	APOC1,APOC2,APOC3
GO: Biological Process	GO:0071830	triglyceride-rich lipoprotein particle clearance	1.65E-06	2.96E-03	6.58E-05	5.31E-04	3	7	APOC1,APOC2,APOC3
GO: Biological Process	GO:0034382	chylomicron remnant clearance	1.65E-06	2.96E-03	6.58E-05	5.31E-04	3	7	APOC1,APOC2,APOC3
GO: Biological Process	GO:0015914	phospholipid transport	2.10E-06	3.76E-03	8.18E-05	6.60E-04	5	57	APOA2,APOA4,APOC1,APOC2,APOC3
GO: Biological Process	GO:0030168	platelet activation	2.18E-06	3.91E-03	8.33E-05	6.72E-04	8	233	SAA1,SERPINA1,FGA,FGB,SERPING1,FGG,F2,PROS1
GO: Biological Process	GO:0010873	positive regulation of cholesterol esterification	2.63E-06	4.72E-03	9.74E-05	7.86E-04	3	8	APOA2,APOA4,APOC1
GO: Biological Process	GO:0010035	response to inorganic substance	2.66E-06	4.77E-03	9.74E-05	7.86E-04	10	415	SERPINA1,HP,FGA,FGB,PON1,APOA4,FGG,HBB,GSN,CRP
GO: Biological Process	GO:0032369	negative regulation of lipid transport	3.21E-06	5.76E-03	1.11E-04	8.94E-04	4	28	APOA2,APOC1,APOC2,APOC3
GO: Biological Process	GO:0046464	acylglycerol catabolic process	3.21E-06	5.76E-03	1.11E-04	8.94E-04	4	28	APOA2,APOA4,APOC2,APOC3
GO: Biological Process	GO:0046461	neutral lipid catabolic process	3.21E-06	5.76E-03	1.11E-04	8.94E-04	4	28	APOA2,APOA4,APOC2,APOC3
GO: Biological Process	GO:0034384	high-density lipoprotein particle clearance	3.94E-06	7.07E-03	1.31E-04	1.06E-03	3	9	APOA2,APOC2,APOC3
GO: Biological Process	GO:0034116	positive regulation of heterotypic cell-cell adhesion	3.94E-06	7.07E-03	1.31E-04	1.06E-03	3	9	FGA,FGB,FGG
GO: Biological Process	GO:0034381	plasma lipoprotein particle clearance	4.89E-06	8.78E-03	1.60E-04	1.29E-03	4	31	APOA2,APOC1,APOC2,APOC3
GO: Biological Process	GO:0050878	regulation of body fluid levels	5.29E-06	9.49E-03	1.69E-04	1.37E-03	12	670	SAA1,SERPINA1,ACTB,FGA,FGB,SERPING1,FGG,F2,HBB,HBD,SERPIND1,PROS1
GO: Biological Process	GO:0042304	regulation of fatty acid biosynthetic process	5.58E-06	1.00E-02	1.71E-04	1.38E-03	4	32	APOA4,APOC1,APOC2,APOC3
GO: Biological Process	GO:0010872	regulation of cholesterol esterification	5.61E-06	1.01E-02	1.71E-04	1.38E-03	3	10	APOA2,APOA4,APOC1
GO: Biological Process	GO:0010896	regulation of triglyceride catabolic process	5.61E-06	1.01E-02	1.71E-04	1.38E-03	3	10	APOA4,APOC2,APOC3
GO: Biological Process	GO:0042632	cholesterol homeostasis	5.84E-06	1.05E-02	1.75E-04	1.41E-03	5	70	ALMS1,APOA2,APOA4,APOC2,APOC3
GO: Biological Process	GO:0055092	sterol homeostasis	6.26E-06	1.12E-02	1.84E-04	1.49E-03	5	71	ALMS1,APOA2,APOA4,APOC2,APOC3
GO: Biological Process	GO:0008203	cholesterol metabolic process	6.59E-06	1.18E-02	1.90E-04	1.54E-03	6	125	SAA1,PON1,APOA2,APOA4,APOC1,APOC3
GO: Biological Process	GO:0051346	negative regulation of hydrolase activity	6.69E-06	1.20E-02	1.90E-04	1.54E-03	9	361	SERPINA3,COL6A3,SERPINA1,SERPING1,APOA2,APOC1,APOC3,SERPIND1,PROS1
GO: Biological Process	GO:0051004	regulation of lipoprotein lipase activity	7.15E-06	1.28E-02	2.01E-04	1.62E-03	4	34	APOA4,APOC1,APOC2,APOC3
GO: Biological Process	GO:0042157	lipoprotein metabolic process	8.63E-06	1.55E-02	2.38E-04	1.92E-03	6	131	PON1,APOA2,APOA4,APOC1,APOC2,APOC3
GO: Biological Process	GO:0044092	negative regulation of molecular function	9.71E-06	1.74E-02	2.64E-04	2.13E-03	14	972	SERPINA3,COL6A3,SERPINA1,NF1,SBF1,HP,SERPING1,APOA2,APOC1,APOC2,APOC3,SERPIND1,HEXIM1,PROS1
GO: Biological Process	GO:0007018	microtubule-based movement	9.87E-06	1.77E-02	2.64E-04	2.13E-03	7	204	DNAH12,DST,KIF27,DNAH10,DNAH5,DNAH8,DNAH11
GO: Biological Process	GO:0051128	regulation of cellular component organization	1.00E-05	1.80E-02	2.65E-04	2.14E-03	19	1733	SHROOM3,MYH9,DST,ANK3,ALMS1,SBF1,SPTAN1,FGA,FGB,APOA2,APOC1,APOC2,APOC3,FGG,F2,NIPBL,GSN,CRP,PROS1
GO: Biological Process	GO:0016125	sterol metabolic process	1.16E-05	2.09E-02	3.01E-04	2.43E-03	6	138	SAA1,PON1,APOA2,APOA4,APOC1,APOC3
GO: Biological Process	GO:0043933	macromolecular complex subunit organization	1.17E-05	2.11E-02	3.01E-04	2.43E-03	19	1752	MYH9,DST,NF1,MDN1,ALMS1,SPTAN1,ASPM,FGA,FGB,APOA2,APOA4,APOC1,APOC2,APOC3,FGG,HBB,GSN,CRP,PCNT
GO: Biological Process	GO:0034433	steroid esterification	1.33E-05	2.38E-02	3.16E-04	2.55E-03	3	13	APOA2,APOA4,APOC1
GO: Biological Process	GO:0010984	regulation of lipoprotein particle clearance	1.33E-05	2.38E-02	3.16E-04	2.55E-03	3	13	APOC1,APOC2,APOC3
GO: Biological Process	GO:0034435	cholesterol esterification	1.33E-05	2.38E-02	3.16E-04	2.55E-03	3	13	APOA2,APOA4,APOC1
GO: Biological Process	GO:0034434	sterol esterification	1.33E-05	2.38E-02	3.16E-04	2.55E-03	3	13	APOA2,APOA4,APOC1
GO: Biological Process	GO:2000910	negative regulation of sterol import	1.34E-05	2.40E-02	3.16E-04	2.55E-03	2	2	APOA2,APOC3
GO: Biological Process	GO:0060621	negative regulation of cholesterol import	1.34E-05	2.40E-02	3.16E-04	2.55E-03	2	2	APOA2,APOC3
GO: Biological Process	GO:0015748	organophosphate ester transport	1.43E-05	2.57E-02	3.34E-04	2.69E-03	5	84	APOA2,APOA4,APOC1,APOC2,APOC3
GO: Biological Process	GO:0001523	retinoid metabolic process	1.61E-05	2.88E-02	3.69E-04	2.98E-03	5	86	APOA2,APOA4,APOC2,APOC3,TTR
GO: Biological Process	GO:0051223	regulation of protein transport	1.64E-05	2.95E-02	3.73E-04	3.01E-03	9	404	SAA1,NF1,ANK3,GCC2,FGA,FGB,APOA2,FGG,PCNT
GO: Biological Process	GO:0048261	negative regulation of receptor-mediated endocytosis	1.68E-05	3.02E-02	3.74E-04	3.02E-03	3	14	APOC1,APOC2,APOC3
GO: Biological Process	GO:0000902	cell morphogenesis	1.69E-05	3.03E-02	3.74E-04	3.02E-03	15	1165	COL6A3,SHROOM3,MYH9,DST,KIF27,ANK3,ALMS1,SPTAN1,ACTB,FGA,FGB,FGG,F2,GSN,PCNT
GO: Biological Process	GO:0010876	lipid localization	1.81E-05	3.24E-02	3.95E-04	3.19E-03	8	311	PON1,APOA2,APOA4,APOC1,APOC2,APOC3,LBP,CRP
GO: Biological Process	GO:0051241	negative regulation of multicellular organismal process	1.99E-05	3.58E-02	4.31E-04	3.48E-03	9	414	ALMS1,SERPING1,APOA2,APOC1,APOC2,APOC3,LBP,F2,PROS1
GO: Biological Process	GO:0022409	positive regulation of cell-cell adhesion	2.04E-05	3.65E-02	4.35E-04	3.51E-03	4	44	ANK3,FGA,FGB,FGG
GO: Biological Process	GO:0060192	negative regulation of lipase activity	2.10E-05	3.77E-02	4.43E-04	3.58E-03	3	15	APOA2,APOC1,APOC3
GO: Biological Process	GO:0007603	phototransduction, visible light	2.23E-05	4.00E-02	4.60E-04	3.71E-03	5	92	APOA2,APOA4,APOC2,APOC3,TTR
GO: Biological Process	GO:0016101	diterpenoid metabolic process	2.23E-05	4.00E-02	4.60E-04	3.71E-03	5	92	APOA2,APOA4,APOC2,APOC3,TTR
GO: Biological Process	GO:1902041	regulation of extrinsic apoptotic signaling pathway via	2.43E-05	4.37E-02	4.96E-04	4.00E-03	4	46	NF1,FGA,FGB,FGG
GO: Biological Process	GO:0006952	defense response	2.48E-05	4.45E-02	5.00E-04	4.04E-03	17	1515	SERPINA3,ORM1,ORM2,SAA1,SERPINA1,SAA2,HP,ACTB,SERPING1,C1R,APOA2,APOA4,APOC3,LBP,F2,CRP,PROS1
GO: Biological Process	GO:0034114	regulation of heterotypic cell-cell adhesion	3.12E-05	5.60E-02	6.22E-04	5.02E-03	3	17	FGA,FGB,FGG
GO: Biological Process	GO:0032880	regulation of protein localization	3.34E-05	5.99E-02	6.58E-04	5.31E-03	10	555	SAA1,NF1,ANK3,MYO5B,GCC2,FGA,FGB,APOA2,FGG,PCNT
GO: Biological Process	GO:0032989	cellular component morphogenesis	3.57E-05	6.40E-02	6.96E-04	5.62E-03	15	1242	COL6A3,SHROOM3,MYH9,DST,KIF27,ANK3,ALMS1,SPTAN1,ACTB,FGA,FGB,FGG,F2,GSN,PCNT
GO: Biological Process	GO:0006721	terpenoid metabolic process	3.67E-05	6.58E-02	7.08E-04	5.71E-03	5	102	APOA2,APOA4,APOC2,APOC3,TTR
GO: Biological Process	GO:0030155	regulation of cell adhesion	3.79E-05	6.80E-02	7.23E-04	5.83E-03	8	345	SAA1,NF1,ANK3,FGA,FGB,LAMA3,FGG,GSN
GO: Biological Process	GO:2000909	regulation of sterol import	3.99E-05	7.16E-02	7.23E-04	5.83E-03	2	3	APOA2,APOC3
GO: Biological Process	GO:0034444	regulation of plasma lipoprotein particle oxidation	3.99E-05	7.16E-02	7.23E-04	5.83E-03	2	3	PON1,APOA4
GO: Biological Process	GO:0060620	regulation of cholesterol import	3.99E-05	7.16E-02	7.23E-04	5.83E-03	2	3	APOA2,APOC3
GO: Biological Process	GO:0034445	negative regulation of plasma lipoprotein particle oxidation	3.99E-05	7.16E-02	7.23E-04	5.83E-03	2	3	PON1,APOA4
GO: Biological Process	GO:0010903	negative regulation of very-low-density lipoprotein particle remodeling	3.99E-05	7.16E-02	7.23E-04	5.83E-03	2	3	APOA2,APOC3
GO: Biological Process	GO:0055088	lipid homeostasis	4.03E-05	7.23E-02	7.23E-04	5.83E-03	5	104	ALMS1,APOA2,APOA4,APOC2,APOC3
GO: Biological Process	GO:0009584	detection of visible light	4.83E-05	8.66E-02	8.58E-04	6.92E-03	5	108	APOA2,APOA4,APOC2,APOC3,TTR
GO: Biological Process	GO:0070201	regulation of establishment of protein localization	5.19E-05	9.31E-02	8.96E-04	7.23E-03	9	468	SAA1,NF1,ANK3,GCC2,FGA,FGB,APOA2,FGG,PCNT
GO: Biological Process	GO:2000352	negative regulation of endothelial cell apoptotic process	5.19E-05	9.32E-02	8.96E-04	7.23E-03	3	20	FGA,FGB,FGG
GO: Biological Process	GO:1900026	positive regulation of substrate adhesion-dependent cell spreading	5.19E-05	9.32E-02	8.96E-04	7.23E-03	3	20	FGA,FGB,FGG
GO: Biological Process	GO:0007010	cytoskeleton organization	5.38E-05	9.66E-02	9.20E-04	7.42E-03	13	987	SHROOM3,MYH9,DST,NF1,ANK3,ALMS1,GCC2,SPTAN1,SYNE2,NUMA1,ASPM,GSN,PCNT
GO: Biological Process	GO:0007602	phototransduction	5.75E-05	1.03E-01	9.72E-04	7.85E-03	5	112	APOA2,APOA4,APOC2,APOC3,TTR
GO: Biological Process	GO:0050996	positive regulation of lipid catabolic process	6.04E-05	1.08E-01	1.00E-03	8.10E-03	3	21	APOA2,APOA4,APOC2
GO: Biological Process	GO:0050995	negative regulation of lipid catabolic process	6.04E-05	1.08E-01	1.00E-03	8.10E-03	3	21	APOA2,APOC1,APOC3
GO: Biological Process	GO:0006641	triglyceride metabolic process	6.52E-05	1.17E-01	1.07E-03	8.66E-03	5	115	APOA2,APOA4,APOC1,APOC2,APOC3
GO: Biological Process	GO:0042744	hydrogen peroxide catabolic process	6.98E-05	1.25E-01	1.13E-03	9.12E-03	3	22	HP,APOA4,HBB
GO: Biological Process	GO:0006869	lipid transport	6.99E-05	1.26E-01	1.13E-03	9.12E-03	7	277	PON1,APOA2,APOA4,APOC1,APOC2,APOC3,LBP
GO: Biological Process	GO:0048870	cell motility	7.54E-05	1.35E-01	1.20E-03	9.66E-03	14	1170	SAA1,MYH9,DST,NF1,SYNE2,ASPM,LAMA3,LBP,F2,DNAH5,DNAH8,PROS1,PCNT,DNAH11
GO: Biological Process	GO:0051674	localization of cell	7.54E-05	1.35E-01	1.20E-03	9.66E-03	14	1170	SAA1,MYH9,DST,NF1,SYNE2,ASPM,LAMA3,LBP,F2,DNAH5,DNAH8,PROS1,PCNT,DNAH11
GO: Biological Process	GO:0051258	protein polymerization	7.67E-05	1.38E-01	1.21E-03	9.74E-03	6	193	SPTAN1,FGA,FGB,FGG,GSN,CRP
GO: Biological Process	GO:0034443	negative regulation of lipoprotein oxidation	7.97E-05	1.43E-01	1.22E-03	9.82E-03	2	4	PON1,APOA4
GO: Biological Process	GO:0034371	chylomicron remodeling	7.97E-05	1.43E-01	1.22E-03	9.82E-03	2	4	APOA4,APOC2
GO: Biological Process	GO:0034441	plasma lipoprotein particle oxidation	7.97E-05	1.43E-01	1.22E-03	9.82E-03	2	4	PON1,APOA4
GO: Biological Process	GO:0072378	blood coagulation, fibrin clot formation	8.00E-05	1.44E-01	1.22E-03	9.82E-03	3	23	FGA,SERPING1,F2
GO: Biological Process	GO:0015850	organic hydroxy compound transport	8.12E-05	1.46E-01	1.22E-03	9.88E-03	6	195	PON1,APOA2,APOA4,APOC1,APOC2,APOC3
GO: Biological Process	GO:0030198	extracellular matrix organization	8.19E-05	1.47E-01	1.22E-03	9.88E-03	8	385	COL6A3,DST,NF1,FGA,FGB,LAMA3,FGG,TTR
GO: Biological Process	GO:0043062	extracellular structure organization	8.34E-05	1.50E-01	1.24E-03	9.97E-03	8	386	COL6A3,DST,NF1,FGA,FGB,LAMA3,FGG,TTR
GO: Cellular Component	GO:0072562	blood microparticle	1.50E-29	3.80E-27	3.80E-27	2.32E-26	21	132	SERPINA3,ORM1,ORM2,HP,AFM,HPR,ACTB,FGA,FGB,PON1,SERPING1,C1R,APOA2,APOA4,FGG,HBA1,F2,HBB,HBD,GSN,PROS1
GO: Cellular Component	GO:0005615	extracellular space	2.81E-23	7.11E-21	3.55E-21	2.17E-20	35	1284	QSOX1,SERPINA3,ORM1,ORM2,COL6A3,SAA1,DMXL2,SERPINA1,SAA2,HP,AFM,HPR,ACTB,FGA,FGB,PON1,SERPING1,C1R,APOA2,APOA4,APOC1,APOC2,APOC3,LBP,FGG,HBA1,F2,HBB,HBD,TTR,LRG1,SERPIND1,GSN,CRP,PROS1
GO: Cellular Component	GO:0034364	high-density lipoprotein particle	3.88E-16	9.81E-14	3.27E-14	2.00E-13	9	28	SAA1,SAA2,HPR,PON1,APOA2,APOA4,APOC1,APOC2,APOC3
GO: Cellular Component	GO:0034358	plasma lipoprotein particle	1.48E-14	3.75E-12	9.38E-13	5.74E-12	9	40	SAA1,SAA2,HPR,PON1,APOA2,APOA4,APOC1,APOC2,APOC3
GO: Cellular Component	GO:0032994	protein-lipid complex	2.41E-14	6.09E-12	1.22E-12	7.44E-12	9	42	SAA1,SAA2,HPR,PON1,APOA2,APOA4,APOC1,APOC2,APOC3
GO: Cellular Component	GO:0060205	cytoplasmic membrane-bounded vesicle lumen	1.00E-11	2.53E-09	4.22E-10	2.58E-09	9	79	SAA1,SERPINA1,HP,FGA,FGB,SERPING1,FGG,HBB,PROS1
GO: Cellular Component	GO:0031983	vesicle lumen	1.41E-11	3.57E-09	5.10E-10	3.12E-09	9	82	SAA1,SERPINA1,HP,FGA,FGB,SERPING1,FGG,HBB,PROS1
GO: Cellular Component	GO:0034366	spherical high-density lipoprotein particle	2.91E-11	7.36E-09	9.20E-10	5.62E-09	5	8	HPR,PON1,APOA2,APOC2,APOC3
GO: Cellular Component	GO:0042627	chylomicron	4.07E-10	1.03E-07	1.14E-08	6.99E-08	5	12	APOA2,APOA4,APOC1,APOC2,APOC3
GO: Cellular Component	GO:0034385	triglyceride-rich lipoprotein particle	7.79E-09	1.97E-06	1.79E-07	1.10E-06	5	20	APOA2,APOA4,APOC1,APOC2,APOC3
GO: Cellular Component	GO:0034361	very-low-density lipoprotein particle	7.79E-09	1.97E-06	1.79E-07	1.10E-06	5	20	APOA2,APOA4,APOC1,APOC2,APOC3
GO: Cellular Component	GO:0031093	platelet alpha granule lumen	1.91E-08	4.83E-06	4.02E-07	2.46E-06	6	48	SERPINA1,FGA,FGB,SERPING1,FGG,PROS1
GO: Cellular Component	GO:0031091	platelet alpha granule	8.32E-08	2.11E-05	1.62E-06	9.90E-06	6	61	SERPINA1,FGA,FGB,SERPING1,FGG,PROS1
GO: Cellular Component	GO:0034774	secretory granule lumen	9.19E-08	2.32E-05	1.66E-06	1.02E-05	6	62	SERPINA1,FGA,FGB,SERPING1,FGG,PROS1
GO: Cellular Component	GO:0005874	microtubule	1.30E-07	3.28E-05	2.19E-06	1.34E-05	11	388	SHROOM3,DNAH12,DST,KIF27,DNAH10,NUMA1,ASPM,DNAH5,DNAH8,PCNT,DNAH11
GO: Cellular Component	GO:0031838	haptoglobin-hemoglobin complex	1.79E-07	4.52E-05	2.83E-06	1.73E-05	3	4	HP,HBA1,HBB
GO: Cellular Component	GO:0005856	cytoskeleton	2.23E-07	5.64E-05	3.32E-06	2.03E-05	23	1993	SHROOM3,MYH9,DNAH12,DST,NF1,MDN1,KIF27,ANK3,ALMS1,DNAH10,MYO5B,SPTAN1,SYNE2,ACTB,NUMA1,ASPM,EVPL,PIBF1,DNAH5,DNAH8,GSN,PCNT,DNAH11
GO: Cellular Component	GO:0005858	axonemal dynein complex	2.72E-07	6.88E-05	3.82E-06	2.34E-05	4	16	DNAH10,DNAH5,DNAH8,DNAH11
GO: Cellular Component	GO:0044433	cytoplasmic vesicle part	3.50E-07	8.85E-05	4.49E-06	2.75E-05	12	527	SAA1,DMXL2,SERPINA1,MYO5B,HP,FGA,FGB,SERPING1,FGG,HBB,DYSF,PROS1
GO: Cellular Component	GO:0030286	dynein complex	3.55E-07	8.98E-05	4.49E-06	2.75E-05	5	41	DNAH12,DNAH10,DNAH5,DNAH8,DNAH11
GO: Cellular Component	GO:0015630	microtubule cytoskeleton	1.44E-06	3.65E-04	1.70E-05	1.04E-04	15	971	SHROOM3,MYH9,DNAH12,DST,KIF27,ALMS1,DNAH10,SPTAN1,NUMA1,ASPM,PIBF1,DNAH5,DNAH8,PCNT,DNAH11
GO: Cellular Component	GO:0042995	cell projection	1.48E-06	3.73E-04	1.70E-05	1.04E-04	20	1711	FSIP2,MYH9,DNAH12,DST,NF1,KIF27,ANK3,ALMS1,DNAH10,MYO5B,SPTAN1,SYNE2,ACTB,NUMA1,DYSF,DNAH5,DNAH8,GSN,CRP,DNAH11
GO: Cellular Component	GO:0005577	fibrinogen complex	2.48E-06	6.27E-04	2.72E-05	1.67E-04	3	8	FGA,FGB,FGG
GO: Cellular Component	GO:0044430	cytoskeletal part	3.20E-06	8.09E-04	3.37E-05	2.06E-04	18	1477	SHROOM3,MYH9,DNAH12,DST,NF1,KIF27,ALMS1,DNAH10,MYO5B,SPTAN1,ACTB,NUMA1,ASPM,PIBF1,DNAH5,DNAH8,PCNT,DNAH11
GO: Cellular Component	GO:0044447	axoneme part	3.94E-06	9.97E-04	3.84E-05	2.35E-04	4	30	DNAH10,DNAH5,DNAH8,DNAH11
GO: Cellular Component	GO:0005868	cytoplasmic dynein complex	3.94E-06	9.97E-04	3.84E-05	2.35E-04	4	30	DNAH10,DNAH5,DNAH8,DNAH11
GO: Cellular Component	GO:0005833	hemoglobin complex	9.63E-06	2.44E-03	9.02E-05	5.52E-04	3	12	HBA1,HBB,HBD
GO: Cellular Component	GO:0005875	microtubule associated complex	1.04E-05	2.62E-03	9.36E-05	5.72E-04	6	138	DNAH12,KIF27,DNAH10,DNAH5,DNAH8,DNAH11
GO: Cellular Component	GO:0005938	cell cortex	1.88E-05	4.77E-03	1.64E-04	1.00E-03	7	230	MYH9,DST,SPTAN1,ACTB,FGA,FGB,FGG
GO: Cellular Component	GO:0071682	endocytic vesicle lumen	2.43E-05	6.14E-03	2.05E-04	1.25E-03	3	16	SAA1,HP,HBB
GO: Cellular Component	GO:0034363	intermediate-density lipoprotein particle	7.65E-05	1.93E-02	6.24E-04	3.81E-03	2	4	APOC2,APOC3
GO: Cellular Component	GO:0016023	cytoplasmic membrane-bounded vesicle	9.22E-05	2.33E-02	7.29E-04	4.45E-03	13	1062	SAA1,DMXL2,SERPINA1,DST,MYO5B,HP,FGA,FGB,SERPING1,FGG,HBB,DYSF,PROS1
GO: Cellular Component	GO:0005929	cilium	1.30E-04	3.28E-02	9.95E-04	6.08E-03	8	420	FSIP2,DNAH12,KIF27,ALMS1,DNAH10,DNAH5,DNAH8,DNAH11
GO: Cellular Component	GO:0097014	ciliary cytoplasm	1.89E-04	4.79E-02	1.35E-03	8.26E-03	4	79	DNAH10,DNAH5,DNAH8,DNAH11
GO: Cellular Component	GO:0005930	axoneme	1.89E-04	4.79E-02	1.35E-03	8.26E-03	4	79	DNAH10,DNAH5,DNAH8,DNAH11
GO: Cellular Component	GO:0070161	anchoring junction	1.92E-04	4.86E-02	1.35E-03	8.26E-03	6	233	SHROOM3,MYH9,SPTAN1,SYNE2,LAMA3,EVPL
GO: Cellular Component	GO:0031410	cytoplasmic vesicle	2.28E-04	5.76E-02	1.56E-03	9.51E-03	13	1163	SAA1,DMXL2,SERPINA1,DST,MYO5B,HP,FGA,FGB,SERPING1,FGG,HBB,DYSF,PROS1

Table S2.3 Survivors patients D7 samples.

Category	ID	Name	p-value	q-value Bonferroni	q-value FDR B&H	q-value FDR B&Y	Hit Count in Query List	Hit Count in Genome	Hit in Query List
GO: Molecular Function	GO:0003774	motor activity	1.46E-10	4.48E-08	4.48E-08	2.83E-07	10	130	MYH9,KIF27,MYH13,DNAH10,MYO5B,CENPE,MYO7A,MYO9A,DNAH5,DNAH11
GO: Molecular Function	GO:0031210	phosphatidylcholine binding	6.39E-09	1.96E-06	8.94E-07	5.64E-06	5	17	NF1,JCHAIN,APOA2,APOA4,APOC1
GO: Molecular Function	GO:0050997	quaternary ammonium group binding	8.73E-09	2.68E-06	8.94E-07	5.64E-06	6	37	NF1,JCHAIN,APOA2,APOA4,APOC1,CRP
GO: Molecular Function	GO:0016462	pyrophosphatase activity	3.18E-08	9.76E-06	1.75E-06	1.10E-05	17	821	BPTF,MYH9,MDN1,KIF27,MYH13,DNAH10,MYO5B,CENPE,CLU,MYO7A,MYO9A,TRPM2,HLTF,RNF213,SRCAP,DNAH5,DNAH11
GO: Molecular Function	GO:0016818	hydrolase activity, acting on acid anhydrides, in phosphorus-containing anhydrides	3.30E-08	1.01E-05	1.75E-06	1.10E-05	17	823	BPTF,MYH9,MDN1,KIF27,MYH13,DNAH10,MYO5B,CENPE,CLU,MYO7A,MYO9A,TRPM2,HLTF,RNF213,SRCAP,DNAH5,DNAH11
GO: Molecular Function	GO:0016817	hydrolase activity, acting on acid anhydrides	3.41E-08	1.05E-05	1.75E-06	1.10E-05	17	825	BPTF,MYH9,MDN1,KIF27,MYH13,DNAH10,MYO5B,CENPE,CLU,MYO7A,MYO9A,TRPM2,HLTF,RNF213,SRCAP,DNAH5,DNAH11
GO: Molecular Function	GO:0017111	nucleoside-triphosphatase activity	1.00E-07	3.08E-05	4.05E-06	2.55E-05	16	781	BPTF,MYH9,MDN1,KIF27,MYH13,DNAH10,MYO5B,CENPE,CLU,MYO7A,MYO9A,HLTF,RNF213,SRCAP,DNAH5,DNAH11
GO: Molecular Function	GO:0016887	ATPase activity	1.05E-07	3.24E-05	4.05E-06	2.55E-05	12	407	BPTF,MYH9,MDN1,KIF27,DNAH10,CENPE,CLU,MYO7A,HLTF,RNF213,DNAH5,DNAH11
GO: Molecular Function	GO:0008092	cytoskeletal protein binding	1.22E-07	3.73E-05	4.14E-06	2.61E-05	16	792	SPTBN4,MYH9,NF1,KIF27,MYH13,MYO5B,CENPE,MYO7A,MYO9A,SYNE2,CEP290,ACTA1,ACTB,OBSCN,TTN,GSN
GO: Molecular Function	GO:0005516	calmodulin binding	6.39E-07	1.96E-04	1.90E-05	1.20E-04	8	175	MYH9,MYH13,MYO5B,MYO7A,OBSCN,ASPM,TTN,PCNT
GO: Molecular Function	GO:0060228	phosphatidylcholine-sterol O-acyltransferase activator activity	6.81E-07	2.09E-04	1.90E-05	1.20E-04	3	5	APOA2,APOA4,APOC1
GO: Molecular Function	GO:0000146	microfilament motor activity	1.55E-06	4.76E-04	3.74E-05	2.36E-04	4	21	MYH9,MYH13,MYO5B,MYO7A
GO: Molecular Function	GO:0005524	ATP binding	1.58E-06	4.86E-04	3.74E-05	2.36E-04	20	1476	MYH9,MDN1,KIF27,MYH13,DNAH10,MYO5B,SACS,CENPE,MYO7A,MYO9A,ACTA1,ACTB,OBSCN,HLTF,RNF213,ATM,SRCAP,DNAH5,TTN,DNAH11
GO: Molecular Function	GO:0032559	adenyl ribonucleotide binding	2.42E-06	7.42E-04	5.30E-05	3.34E-04	20	1517	MYH9,MDN1,KIF27,MYH13,DNAH10,MYO5B,SACS,CENPE,MYO7A,MYO9A,ACTA1,ACTB,OBSCN,HLTF,RNF213,ATM,SRCAP,DNAH5,TTN,DNAH11
GO: Molecular Function	GO:0030554	adenyl nucleotide binding	2.93E-06	8.98E-04	5.99E-05	3.78E-04	20	1536	MYH9,MDN1,KIF27,MYH13,DNAH10,MYO5B,SACS,CENPE,MYO7A,MYO9A,ACTA1,ACTB,OBSCN,HLTF,RNF213,ATM,SRCAP,DNAH5,TTN,DNAH11
GO: Molecular Function	GO:0030492	hemoglobin binding	3.78E-06	1.16E-03	6.97E-05	4.40E-04	3	8	HP,HPR,HBB
GO: Molecular Function	GO:0043178	alcohol binding	3.86E-06	1.19E-03	6.97E-05	4.40E-04	6	101	NF1,JCHAIN,APOA2,APOA4,APOC1,CRP
GO: Molecular Function	GO:0016209	antioxidant activity	1.21E-05	3.71E-03	2.06E-04	1.30E-03	5	72	HP,GPX3,APOA4,HBA1,HBB
GO: Molecular Function	GO:0003777	microtubule motor activity	1.68E-05	5.16E-03	2.72E-04	1.71E-03	5	77	KIF27,DNAH10,CENPE,DNAH5,DNAH11
GO: Molecular Function	GO:0035639	purine ribonucleoside triphosphate binding	3.36E-05	1.03E-02	4.92E-04	3.10E-03	20	1810	MYH9,MDN1,KIF27,MYH13,DNAH10,MYO5B,SACS,CENPE,MYO7A,MYO9A,ACTA1,ACTB,OBSCN,HLTF,RNF213,ATM,SRCAP,DNAH5,TTN,DNAH11
GO: Molecular Function	GO:0003779	actin binding	3.44E-05	1.06E-02	4.92E-04	3.10E-03	9	392	SPTBN4,MYH9,MYH13,MYO5B,MYO7A,MYO9A,SYNE2,TTN,GSN
GO: Molecular Function	GO:0032550	purine ribonucleoside binding	3.73E-05	1.14E-02	4.92E-04	3.10E-03	20	1823	MYH9,MDN1,KIF27,MYH13,DNAH10,MYO5B,SACS,CENPE,MYO7A,MYO9A,ACTA1,ACTB,OBSCN,HLTF,RNF213,ATM,SRCAP,DNAH5,TTN,DNAH11
GO: Molecular Function	GO:0001883	purine nucleoside binding	3.81E-05	1.17E-02	4.92E-04	3.10E-03	20	1826	MYH9,MDN1,KIF27,MYH13,DNAH10,MYO5B,SACS,CENPE,MYO7A,MYO9A,ACTA1,ACTB,OBSCN,HLTF,RNF213,ATM,SRCAP,DNAH5,TTN,DNAH11
GO: Molecular Function	GO:0032549	ribonucleoside binding	3.84E-05	1.18E-02	4.92E-04	3.10E-03	20	1827	MYH9,MDN1,KIF27,MYH13,DNAH10,MYO5B,SACS,CENPE,MYO7A,MYO9A,ACTA1,ACTB,OBSCN,HLTF,RNF213,ATM,SRCAP,DNAH5,TTN,DNAH11
GO: Molecular Function	GO:0001882	nucleoside binding	4.16E-05	1.28E-02	5.10E-04	3.22E-03	20	1837	MYH9,MDN1,KIF27,MYH13,DNAH10,MYO5B,SACS,CENPE,MYO7A,MYO9A,ACTA1,ACTB,OBSCN,HLTF,RNF213,ATM,SRCAP,DNAH5,TTN,DNAH11
GO: Molecular Function	GO:0032555	purine ribonucleotide binding	4.85E-05	1.49E-02	5.73E-04	3.61E-03	20	1857	MYH9,MDN1,KIF27,MYH13,DNAH10,MYO5B,SACS,CENPE,MYO7A,MYO9A,ACTA1,ACTB,OBSCN,HLTF,RNF213,ATM,SRCAP,DNAH5,TTN,DNAH11
GO: Molecular Function	GO:0031720	haptoglobin binding	5.08E-05	1.56E-02	5.77E-04	3.64E-03	2	3	HBA1,HBB
GO: Molecular Function	GO:0032553	ribonucleotide binding	5.48E-05	1.68E-02	6.01E-04	3.79E-03	20	1873	MYH9,MDN1,KIF27,MYH13,DNAH10,MYO5B,SACS,CENPE,MYO7A,MYO9A,ACTA1,ACTB,OBSCN,HLTF,RNF213,ATM,SRCAP,DNAH5,TTN,DNAH11
GO: Molecular Function	GO:0017076	purine nucleotide binding	5.78E-05	1.77E-02	6.11E-04	3.86E-03	20	1880	MYH9,MDN1,KIF27,MYH13,DNAH10,MYO5B,SACS,CENPE,MYO7A,MYO9A,ACTA1,ACTB,OBSCN,HLTF,RNF213,ATM,SRCAP,DNAH5,TTN,DNAH11
GO: Molecular Function	GO:0005198	structural molecule activity	6.38E-05	1.96E-02	6.53E-04	4.12E-03	11	641	SPTBN4,ACTA1,ACTB,OBSCN,FGA,FGB,LAMA3,LAMA4,EVPL,FGG,TTN
GO: Molecular Function	GO:0004857	enzyme inhibitor activity	8.83E-05	2.71E-02	8.74E-04	5.51E-03	8	344	SERPINA3,COL6A3,SERPINA1,SBF1,APOA2,APOC1,SERPIND1,HEXIM1
GO: Molecular Function	GO:0071813	lipoprotein particle binding	1.66E-04	5.11E-02	1.55E-03	9.76E-03	3	26	PON1,APOA2,CRP
GO: Molecular Function	GO:0071814	protein-lipid complex binding	1.66E-04	5.11E-02	1.55E-03	9.76E-03	3	26	PON1,APOA2,CRP
GO: Biological Process	GO:0006953	acute-phase response	4.73E-13	1.01E-09	1.01E-09	8.32E-09	9	49	SERPINA3,ORM1,SAA1,SERPINA1,SAA2,HP,LBP,F2,CRP
GO: Biological Process	GO:0002526	acute inflammatory response	1.60E-11	3.41E-08	1.70E-08	1.41E-07	11	140	SERPINA3,ORM1,SAA1,SERPINA1,SAA2,HP,APOA2,LBP,C6,F2,CRP
GO: Biological Process	GO:0009611	response to wounding	1.03E-09	2.20E-06	7.35E-07	6.06E-06	23	1255	SERPINA3,ORM1,SAA1,SERPINA1,SAA2,NF1,CENPE,CLU,HP,ACTB,FGA,FGB,APOA2,LBP,C6,FGG,F2,HBB,HBD,TTN,SERPIND1,GSN,CRP
GO: Biological Process	GO:0010035	response to inorganic substance	1.85E-09	3.94E-06	9.84E-07	8.11E-06	14	415	SERPINA1,HP,ACTA1,GPX3,FGA,FGB,PON1,CP,APOA4,FGG,HBB,TTN,GSN,CRP
GO: Biological Process	GO:0042060	wound healing	1.98E-08	4.23E-05	8.47E-06	6.98E-05	16	681	SAA1,SERPINA1,NF1,CENPE,CLU,ACTB,FGA,FGB,FGG,F2,HBB,HBD,TTN,SERPIND1,GSN,CRP
GO: Biological Process	GO:0007596	blood coagulation	3.01E-07	6.42E-04	8.93E-05	7.36E-04	13	529	SAA1,SERPINA1,CENPE,CLU,ACTB,FGA,FGB,FGG,F2,HBB,HBD,TTN,SERPIND1
GO: Biological Process	GO:0050817	coagulation	3.21E-07	6.84E-04	8.93E-05	7.36E-04	13	532	SAA1,SERPINA1,CENPE,CLU,ACTB,FGA,FGB,FGG,F2,HBB,HBD,TTN,SERPIND1
GO: Biological Process	GO:0007599	hemostasis	3.35E-07	7.14E-04	8.93E-05	7.36E-04	13	534	SAA1,SERPINA1,CENPE,CLU,ACTB,FGA,FGB,FGG,F2,HBB,HBD,TTN,SERPIND1
GO: Biological Process	GO:0007417	central nervous system development	4.73E-07	1.01E-03	1.09E-04	9.01E-04	16	859	SPTBN4,POTEE,BPTF,NF1,KIF27,CLU,CEP290,ACTB,ASPM,ATM,F2,DNAH5,NIPBL,PCM1,GSN,PCNT
GO: Biological Process	GO:0043933	macromolecular complex subunit organization	5.12E-07	1.09E-03	1.09E-04	9.01E-04	23	1752	SPTBN4,CCDC88C,MYH9,NF1,MDN1,CENPE,CLU,JCHAIN,ACTA1,GPX3,ASPM,FGA,FGB,APOA2,APOA4,APOC1,FGG,HBB,TTN,PCM1,GSN,CRP,PCNT
GO: Biological Process	GO:0006928	movement of cell or subcellular component	1.29E-06	2.75E-03	2.50E-04	2.06E-03	22	1703	SPTBN4,COL6A3,SAA1,MYH9,NF1,KIF27,DNAH10,CENPE,MYO7A,SYNE2,ACTA1,ACTB,ASPM,LAMA3,LAMA4,LBP,F2,DNAH5,TTN,PCM1,PCNT,DNAH11
GO: Biological Process	GO:0002576	platelet degranulation	1.56E-06	3.33E-03	2.77E-04	2.29E-03	6	85	SERPINA1,CLU,FGA,FGB,FGG,TTN
GO: Biological Process	GO:0042744	hydrogen peroxide catabolic process	2.03E-06	4.33E-03	3.33E-04	2.74E-03	4	22	HP,GPX3,APOA4,HBB
GO: Biological Process	GO:0010038	response to metal ion	2.70E-06	5.77E-03	4.12E-04	3.40E-03	9	280	SERPINA1,ACTA1,FGA,FGB,CP,FGG,TTN,GSN,CRP
GO: Biological Process	GO:0032989	cellular component morphogenesis	3.08E-06	6.57E-03	4.26E-04	3.52E-03	18	1242	SPTBN4,COL6A3,MYH9,KIF27,CLU,MYO7A,CEP290,ACTA1,ACTB,OBSCN,FGA,FGB,FGG,F2,TTN,PCM1,GSN,PCNT
GO: Biological Process	GO:0070925	organelle assembly	3.20E-06	6.82E-03	4.26E-04	3.52E-03	10	367	KIF27,CENPE,MYO7A,CEP290,ACTA1,OBSCN,ASPM,TTN,PCM1,PCNT
GO: Biological Process	GO:0032880	regulation of protein localization	3.41E-06	7.28E-03	4.28E-04	3.53E-03	12	555	SAA1,NF1,MYO5B,CENPE,GCC2,CEP290,FGA,FGB,APOA2,FGG,PCM1,PCNT
GO: Biological Process	GO:0010873	positive regulation of cholesterol esterification	3.99E-06	8.51E-03	4.70E-04	3.88E-03	3	8	APOA2,APOA4,APOC1
GO: Biological Process	GO:0050878	regulation of body fluid levels	4.19E-06	8.93E-03	4.70E-04	3.88E-03	13	670	SAA1,SERPINA1,CENPE,CLU,ACTB,FGA,FGB,FGG,F2,HBB,HBD,TTN,SERPIND1
GO: Biological Process	GO:0071822	protein complex subunit organization	5.54E-06	1.18E-02	5.91E-04	4.87E-03	20	1570	SPTBN4,CCDC88C,MYH9,NF1,MDN1,CENPE,CLU,JCHAIN,ACTA1,GPX3,ASPM,FGA,FGB,FGG,HBB,TTN,PCM1,GSN,CRP,PCNT
GO: Biological Process	GO:0034116	positive regulation of heterotypic cell-cell adhesion	5.96E-06	1.27E-02	6.02E-04	4.97E-03	3	9	FGA,FGB,FGG
GO: Biological Process	GO:0030168	platelet activation	6.21E-06	1.33E-02	6.02E-04	4.97E-03	8	233	SAA1,SERPINA1,CLU,FGA,FGB,FGG,F2,TTN
GO: Biological Process	GO:0051223	regulation of protein transport	7.46E-06	1.59E-02	6.54E-04	5.39E-03	10	404	SAA1,NF1,GCC2,CEP290,FGA,FGB,APOA2,FGG,PCM1,PCNT
GO: Biological Process	GO:0001895	retina homeostasis	7.97E-06	1.70E-02	6.54E-04	5.39E-03	5	65	POTEE,POTEF,JCHAIN,ACTB,POTEI
GO: Biological Process	GO:0006200	ATP catabolic process	7.99E-06	1.70E-02	6.54E-04	5.39E-03	9	320	BPTF,MYH9,MDN1,DNAH10,MYO7A,HLTF,RNF213,DNAH5,DNAH11
GO: Biological Process	GO:0010872	regulation of cholesterol esterification	8.50E-06	1.81E-02	6.54E-04	5.39E-03	3	10	APOA2,APOA4,APOC1
GO: Biological Process	GO:0015918	sterol transport	9.26E-06	1.98E-02	6.54E-04	5.39E-03	5	67	CLU,PON1,APOA2,APOA4,APOC1
GO: Biological Process	GO:0030301	cholesterol transport	9.26E-06	1.98E-02	6.54E-04	5.39E-03	5	67	CLU,PON1,APOA2,APOA4,APOC1
GO: Biological Process	GO:0009158	ribonucleoside monophosphate catabolic process	9.27E-06	1.98E-02	6.54E-04	5.39E-03	9	326	BPTF,MYH9,MDN1,DNAH10,MYO7A,HLTF,RNF213,DNAH5,DNAH11
GO: Biological Process	GO:0009169	purine ribonucleoside monophosphate catabolic process	9.27E-06	1.98E-02	6.54E-04	5.39E-03	9	326	BPTF,MYH9,MDN1,DNAH10,MYO7A,HLTF,RNF213,DNAH5,DNAH11
GO: Biological Process	GO:0009128	purine nucleoside monophosphate catabolic process	9.50E-06	2.03E-02	6.54E-04	5.39E-03	9	327	BPTF,MYH9,MDN1,DNAH10,MYO7A,HLTF,RNF213,DNAH5,DNAH11
GO: Biological Process	GO:0009125	nucleoside monophosphate catabolic process	9.98E-06	2.13E-02	6.65E-04	5.48E-03	9	329	BPTF,MYH9,MDN1,DNAH10,MYO7A,HLTF,RNF213,DNAH5,DNAH11
GO: Biological Process	GO:0065003	macromolecular complex assembly	1.14E-05	2.44E-02	7.38E-04	6.09E-03	18	1365	SPTBN4,CCDC88C,MDN1,CENPE,CLU,JCHAIN,GPX3,ASPM,FGA,FGB,APOA2,APOA4,APOC1,FGG,HBB,TTN,GSN,CRP
GO: Biological Process	GO:0048468	cell development	1.39E-05	2.97E-02	8.06E-04	6.65E-03	22	1970	SPTBN4,COL6A3,MYH9,NF1,CLU,MYO7A,CEP290,ACTA1,ACTB,OBSCN,ASPM,FGA,FGB,ATM,FGG,HBA1,F2,TTN,NIPBL,PCM1,GSN,CRP
GO: Biological Process	GO:0032374	regulation of cholesterol transport	1.39E-05	2.97E-02	8.06E-04	6.65E-03	4	35	PON1,APOA2,APOA4,APOC1
GO: Biological Process	GO:0032371	regulation of sterol transport	1.39E-05	2.97E-02	8.06E-04	6.65E-03	4	35	PON1,APOA2,APOA4,APOC1
GO: Biological Process	GO:0051651	maintenance of location in cell	1.40E-05	2.98E-02	8.06E-04	6.65E-03	6	124	SPTBN4,CENPE,GCC2,SYNE2,ASPM,PCM1
GO: Biological Process	GO:0033700	phospholipid efflux	1.55E-05	3.30E-02	8.64E-04	7.13E-03	3	12	APOA2,APOA4,APOC1
GO: Biological Process	GO:0007420	brain development	1.58E-05	3.37E-02	8.64E-04	7.13E-03	12	646	POTEE,BPTF,NF1,KIF27,CEP290,ACTB,ASPM,ATM,DNAH5,NIPBL,PCM1,PCNT
GO: Biological Process	GO:0034433	steroid esterification	2.01E-05	4.28E-02	9.96E-04	8.21E-03	3	13	APOA2,APOA4,APOC1
GO: Biological Process	GO:0033194	response to hydroperoxide	2.01E-05	4.28E-02	9.96E-04	8.21E-03	3	13	TRPM2,GPX3,APOA4
GO: Biological Process	GO:0034435	cholesterol esterification	2.01E-05	4.28E-02	9.96E-04	8.21E-03	3	13	APOA2,APOA4,APOC1
GO: Biological Process	GO:0034434	sterol esterification	2.01E-05	4.28E-02	9.96E-04	8.21E-03	3	13	APOA2,APOA4,APOC1
GO: Biological Process	GO:0001894	tissue homeostasis	2.38E-05	5.07E-02	1.13E-03	9.34E-03	7	203	POTEE,SERPINA3,NF1,POTEF,JCHAIN,ACTB,POTEI
GO: Biological Process	GO:0033344	cholesterol efflux	2.39E-05	5.10E-02	1.13E-03	9.34E-03	4	40	PON1,APOA2,APOA4,APOC1
GO: Biological Process	GO:0034375	high-density lipoprotein particle remodeling	2.55E-05	5.43E-02	1.18E-03	9.73E-03	3	14	APOA2,APOA4,APOC1
GO: Biological Process	GO:0070201	regulation of establishment of protein localization	2.66E-05	5.67E-02	1.21E-03	9.94E-03	10	468	SAA1,NF1,GCC2,CEP290,FGA,FGB,APOA2,FGG,PCM1,PCNT
GO: Cellular Component	GO:0072562	blood microparticle	9.11E-32	2.69E-29	2.69E-29	1.68E-28	23	132	POTEE,SERPINA3,ORM1,CLU,HP,POTEF,HPR,JCHAIN,ACTA1,ACTB,HPX,FGA,FGB,PON1,CP,APOA2,APOA4,FGG,HBA1,F2,HBB,HBD,GSN
GO: Cellular Component	GO:0005615	extracellular space	6.45E-23	1.90E-20	9.51E-21	5.96E-20	37	1284	QSOX1,POTEE,SERPINA3,ORM1,COL6A3,SAA1,DMXL2,SERPINA1,SAA2,CLU,HP,POTEF,HPR,JCHAIN,ACTA1,ACTB,GPX3,HPX,FGA,FGB,PON1,CP,APOA2,APOA4,APOC1,LBP,C6,FGG,POTEI,HBA1,F2,HBB,HBD,LRG1,SERPIND1,GSN,CRP
GO: Cellular Component	GO:0034364	high-density lipoprotein particle	1.72E-13	5.07E-11	1.69E-11	1.06E-10	8	28	SAA1,SAA2,CLU,HPR,PON1,APOA2,APOA4,APOC1
GO: Cellular Component	GO:0034358	plasma lipoprotein particle	4.08E-12	1.21E-09	3.01E-10	1.89E-09	8	40	SAA1,SAA2,CLU,HPR,PON1,APOA2,APOA4,APOC1
GO: Cellular Component	GO:0032994	protein-lipid complex	6.23E-12	1.84E-09	3.67E-10	2.30E-09	8	42	SAA1,SAA2,CLU,HPR,PON1,APOA2,APOA4,APOC1
GO: Cellular Component	GO:0060205	cytoplasmic membrane-bounded vesicle lumen	3.58E-11	1.06E-08	1.76E-09	1.10E-08	9	79	SAA1,SERPINA1,CLU,HP,HPX,FGA,FGB,FGG,HBB
GO: Cellular Component	GO:0031983	vesicle lumen	5.05E-11	1.49E-08	2.13E-09	1.33E-08	9	82	SAA1,SERPINA1,CLU,HP,HPX,FGA,FGB,FGG,HBB
GO: Cellular Component	GO:0016459	myosin complex	9.01E-09	2.66E-06	3.32E-07	2.08E-06	7	65	MYH9,MYH13,MYO5B,MYO7A,MYO9A,OBSCN,TTN
GO: Cellular Component	GO:0005856	cytoskeleton	1.15E-08	3.38E-06	3.76E-07	2.35E-06	27	1993	SPTBN4,BPTF,MYH9,NF1,MDN1,KIF27,MYH13,DNAH10,MYO5B,CENPE,MYO7A,MYO9A,CNTRL,SYNE2,CEP290,ACTA1,ACTB,OBSCN,ASPM,EVPL,ATM,DNAH5,TTN,PCM1,GSN,PCNT,DNAH11
GO: Cellular Component	GO:0034366	spherical high-density lipoprotein particle	1.87E-08	5.51E-06	5.51E-07	3.46E-06	4	8	CLU,HPR,PON1,APOA2
GO: Cellular Component	GO:0044430	cytoskeletal part	7.77E-08	2.29E-05	2.09E-06	1.31E-05	22	1477	SPTBN4,MYH9,NF1,KIF27,MYH13,DNAH10,MYO5B,CENPE,MYO7A,MYO9A,CNTRL,CEP290,ACTA1,ACTB,OBSCN,ASPM,ATM,DNAH5,TTN,PCM1,PCNT,DNAH11
GO: Cellular Component	GO:0031838	haptoglobin-hemoglobin complex	2.71E-07	8.00E-05	6.67E-06	4.18E-05	3	4	HP,HBA1,HBB
GO: Cellular Component	GO:0071682	endocytic vesicle lumen	4.74E-07	1.40E-04	1.08E-05	6.74E-05	4	16	SAA1,HP,HPX,HBB
GO: Cellular Component	GO:0031093	platelet alpha granule lumen	1.58E-06	4.66E-04	3.33E-05	2.09E-04	5	48	SERPINA1,CLU,FGA,FGB,FGG
GO: Cellular Component	GO:0005577	fibrinogen complex	3.75E-06	1.11E-03	7.38E-05	4.62E-04	3	8	FGA,FGB,FGG
GO: Cellular Component	GO:0016460	myosin II complex	4.41E-06	1.30E-03	8.14E-05	5.10E-04	4	27	MYH9,MYH13,OBSCN,TTN
GO: Cellular Component	GO:0005938	cell cortex	4.86E-06	1.44E-03	8.44E-05	5.29E-04	8	230	SPTBN4,MYH9,MYO7A,POTEF,ACTB,FGA,FGB,FGG
GO: Cellular Component	GO:0031091	platelet alpha granule	5.27E-06	1.55E-03	8.63E-05	5.41E-04	5	61	SERPINA1,CLU,FGA,FGB,FGG
GO: Cellular Component	GO:0034774	secretory granule lumen	5.71E-06	1.69E-03	8.87E-05	5.56E-04	5	62	SERPINA1,CLU,FGA,FGB,FGG
GO: Cellular Component	GO:0015629	actin cytoskeleton	7.90E-06	2.33E-03	1.17E-04	7.30E-04	10	415	SPTBN4,MYH9,MYH13,MYO5B,MYO7A,MYO9A,ACTA1,OBSCN,TTN,GSN
GO: Cellular Component	GO:0044433	cytoplasmic vesicle part	1.04E-05	3.08E-03	1.47E-04	9.19E-04	11	527	SAA1,DMXL2,SERPINA1,MYO5B,CLU,HP,HPX,FGA,FGB,FGG,HBB
GO: Cellular Component	GO:0042627	chylomicron	1.46E-05	4.30E-03	1.87E-04	1.17E-03	3	12	APOA2,APOA4,APOC1
GO: Cellular Component	GO:0005833	hemoglobin complex	1.46E-05	4.30E-03	1.87E-04	1.17E-03	3	12	HBA1,HBB,HBD
GO: Cellular Component	GO:0005858	axonemal dynein complex	3.66E-05	1.08E-02	4.32E-04	2.71E-03	3	16	DNAH10,DNAH5,DNAH11
GO: Cellular Component	GO:0034451	centriolar satellite	3.66E-05	1.08E-02	4.32E-04	2.71E-03	3	16	CEP290,PCM1,PCNT
GO: Cellular Component	GO:0005859	muscle myosin complex	7.37E-05	2.17E-02	7.76E-04	4.86E-03	3	20	MYH13,OBSCN,TTN
GO: Cellular Component	GO:0034385	triglyceride-rich lipoprotein particle	7.37E-05	2.17E-02	7.76E-04	4.86E-03	3	20	APOA2,APOA4,APOC1
GO: Cellular Component	GO:0034361	very-low-density lipoprotein particle	7.37E-05	2.17E-02	7.76E-04	4.86E-03	3	20	APOA2,APOA4,APOC1
GO: Cellular Component	GO:0032982	myosin filament	9.90E-05	2.92E-02	1.01E-03	6.31E-03	3	22	MYH9,MYH13,OBSCN
GO: Cellular Component	GO:0036379	myofilament	1.14E-04	3.35E-02	1.12E-03	6.99E-03	3	23	ACTA1,OBSCN,TTN
GO: Cellular Component	GO:0015630	microtubule cytoskeleton	1.61E-04	4.75E-02	1.53E-03	9.60E-03	13	971	BPTF,MYH9,KIF27,DNAH10,CENPE,CNTRL,CEP290,ASPM,ATM,DNAH5,PCM1,PCNT,DNAH11

Table S2.4 Non-survivors patients D7 samples.

Category	ID	Name	p-value	q-value Bonferroni	q-value FDR B&H	q-value FDR B&Y	Hit Count in Query List	Hit Count in Genome	Hit in Query List
GO: Molecular Function	GO:0031210	phosphatidylcholine binding	4.85E-09	1.35E-06	8.76E-07	5.44E-06	5	17	NF1,JCHAIN,APOA2,APOA4,APOC1
GO: Molecular Function	GO:0050997	quaternary ammonium group binding	6.28E-09	1.75E-06	8.76E-07	5.44E-06	6	37	NF1,JCHAIN,APOA2,APOA4,APOC1,CRP
GO: Molecular Function	GO:0060228	phosphatidylcholine-sterol O-acyltransferase activator activity	5.78E-07	1.61E-04	5.38E-05	3.34E-04	3	5	APOA2,APOA4,APOC1
GO: Molecular Function	GO:0043178	alcohol binding	2.81E-06	7.84E-04	1.79E-04	1.11E-03	6	101	NF1,JCHAIN,APOA2,APOA4,APOC1,CRP
GO: Molecular Function	GO:0030492	hemoglobin binding	3.21E-06	8.96E-04	1.79E-04	1.11E-03	3	8	HP,HPR,HBB
GO: Molecular Function	GO:0004857	enzyme inhibitor activity	7.84E-06	2.19E-03	3.64E-04	2.26E-03	9	344	SERPINA3,COL6A3,SERPINA4,SBF1,SERPING1,APOA2,APOC1,SERPIND1,HEXIM1
GO: Molecular Function	GO:0008092	cytoskeletal protein binding	1.11E-05	3.10E-03	4.43E-04	2.75E-03	13	792	SPTBN4,MYH9,CCDC88A,NF1,KIF27,MYO5B,SPTAN1,SYNE2,ACTA1,NUMA1,OBSCN,TTN,GSN
GO: Molecular Function	GO:0008289	lipid binding	2.78E-05	7.75E-03	9.09E-04	5.64E-03	11	619	SPTBN4,CCDC88A,NF1,JCHAIN,PON1,APOA2,APOA4,APOC1,LBP,APOL1,CRP
GO: Molecular Function	GO:0005543	phospholipid binding	2.93E-05	8.18E-03	9.09E-04	5.64E-03	8	311	SPTBN4,CCDC88A,NF1,JCHAIN,PON1,APOA2,APOA4,APOC1
GO: Molecular Function	GO:0004867	serine-type endopeptidase inhibitor activity	3.77E-05	1.05E-02	9.69E-04	6.02E-03	5	96	SERPINA3,COL6A3,SERPINA4,SERPING1,SERPIND1
GO: Molecular Function	GO:0005198	structural molecule activity	3.82E-05	1.07E-02	9.69E-04	6.02E-03	11	641	SPTBN4,SPTAN1,ACTA1,NUMA1,OBSCN,FGA,FGB,LAMA3,EVPL,FGG,TTN
GO: Molecular Function	GO:0031720	haptoglobin binding	4.56E-05	1.27E-02	1.06E-03	6.58E-03	2	3	HBA1,HBB
GO: Molecular Function	GO:0001848	complement binding	6.32E-05	1.76E-02	1.29E-03	7.99E-03	3	20	SERPING1,C8A,CRP
GO: Molecular Function	GO:0005516	calmodulin binding	6.45E-05	1.80E-02	1.29E-03	7.99E-03	6	175	MYH9,MYO5B,SPTAN1,OBSCN,ASPM,TTN
GO: Biological Process	GO:0002526	acute inflammatory response	3.27E-16	6.40E-13	6.40E-13	5.22E-12	14	140	SERPINA3,SAA1,SAA2,VTN,HP,SERPING1,APOA2,LBP,C8A,C9,CFI,F2,KLKB1,CRP
GO: Biological Process	GO:0072376	protein activation cascade	2.80E-12	5.48E-09	2.74E-09	2.23E-08	10	91	CFHR1,VTN,FGA,SERPING1,C8A,C9,CFI,F2,KLKB1,CRP
GO: Biological Process	GO:0009611	response to wounding	6.88E-12	1.35E-08	4.49E-09	3.66E-08	25	1255	SERPINA3,SAA1,SAA2,VTN,NF1,HP,FGA,FGB,SERPING1,APOA2,LBP,FGG,C8A,C9,CFI,F2,HBB,HBD,TTN,KLKB1,LYST,SERPIND1,F13A1,GSN,CRP
GO: Biological Process	GO:0042060	wound healing	1.32E-10	2.59E-07	6.47E-08	5.28E-07	18	681	SAA1,VTN,NF1,FGA,FGB,SERPING1,FGG,C9,F2,HBB,HBD,TTN,KLKB1,LYST,SERPIND1,F13A1,GSN,CRP
GO: Biological Process	GO:0006953	acute-phase response	9.27E-10	1.81E-06	3.63E-07	2.96E-06	7	49	SERPINA3,SAA1,SAA2,HP,LBP,F2,CRP
GO: Biological Process	GO:2000257	regulation of protein activation cascade	1.84E-09	3.61E-06	5.54E-07	4.52E-06	6	30	VTN,FGA,SERPING1,C8A,C9,CFI
GO: Biological Process	GO:0007596	blood coagulation	2.24E-09	4.39E-06	5.54E-07	4.52E-06	15	529	SAA1,VTN,FGA,FGB,SERPING1,FGG,C9,F2,HBB,HBD,TTN,KLKB1,LYST,SERPIND1,F13A1
GO: Biological Process	GO:0050817	coagulation	2.42E-09	4.74E-06	5.54E-07	4.52E-06	15	532	SAA1,VTN,FGA,FGB,SERPING1,FGG,C9,F2,HBB,HBD,TTN,KLKB1,LYST,SERPIND1,F13A1
GO: Biological Process	GO:0007599	hemostasis	2.55E-09	4.99E-06	5.54E-07	4.52E-06	15	534	SAA1,VTN,FGA,FGB,SERPING1,FGG,C9,F2,HBB,HBD,TTN,KLKB1,LYST,SERPIND1,F13A1
GO: Biological Process	GO:0006956	complement activation	1.09E-08	2.14E-05	2.14E-06	1.74E-05	7	69	CFHR1,VTN,SERPING1,C8A,C9,CFI,CRP
GO: Biological Process	GO:0050878	regulation of body fluid levels	5.26E-08	1.03E-04	9.36E-06	7.63E-05	15	670	SAA1,VTN,FGA,FGB,SERPING1,FGG,C9,F2,HBB,HBD,TTN,KLKB1,LYST,SERPIND1,F13A1
GO: Biological Process	GO:0030449	regulation of complement activation	8.21E-08	1.61E-04	1.29E-05	1.05E-04	5	28	VTN,SERPING1,C8A,C9,CFI
GO: Biological Process	GO:0010035	response to inorganic substance	8.75E-08	1.71E-04	1.29E-05	1.05E-04	12	415	HP,ACTA1,FGA,FGB,PON1,CP,APOA4,FGG,HBB,TTN,GSN,CRP
GO: Biological Process	GO:0006954	inflammatory response	9.21E-08	1.80E-04	1.29E-05	1.05E-04	14	599	SERPINA3,SAA1,SAA2,VTN,HP,SERPING1,APOA2,LBP,C8A,C9,CFI,F2,KLKB1,CRP
GO: Biological Process	GO:0002673	regulation of acute inflammatory response	5.01E-07	9.80E-04	6.53E-05	5.33E-04	6	74	VTN,SERPING1,C8A,C9,CFI,KLKB1
GO: Biological Process	GO:0006959	humoral immune response	6.04E-07	1.18E-03	7.39E-05	6.03E-04	8	180	CFHR1,VTN,JCHAIN,SERPING1,C8A,C9,CFI,CRP
GO: Biological Process	GO:0002576	platelet degranulation	1.14E-06	2.24E-03	1.32E-04	1.07E-03	6	85	FGA,FGB,SERPING1,FGG,TTN,F13A1
GO: Biological Process	GO:0002920	regulation of humoral immune response	1.34E-06	2.63E-03	1.46E-04	1.19E-03	5	48	VTN,SERPING1,C8A,C9,CFI
GO: Biological Process	GO:0072378	blood coagulation, fibrin clot formation	1.98E-06	3.88E-03	1.97E-04	1.61E-03	4	23	FGA,SERPING1,F2,KLKB1
GO: Biological Process	GO:0006958	complement activation, classical pathway	2.01E-06	3.94E-03	1.97E-04	1.61E-03	5	52	SERPING1,C8A,C9,CFI,CRP
GO: Biological Process	GO:1903034	regulation of response to wounding	2.61E-06	5.11E-03	2.43E-04	1.99E-03	10	379	SAA1,VTN,FGA,SERPING1,LBP,C8A,C9,CFI,F2,KLKB1
GO: Biological Process	GO:0016192	vesicle-mediated transport	3.24E-06	6.34E-03	2.88E-04	2.35E-03	17	1184	SPTBN4,VTN,MYO5B,GCC2,FGA,FGB,SERPING1,APOC1,LBP,FGG,CFI,TTN,LYST,F13A1,GOLGA4,GSN,CRP
GO: Biological Process	GO:0010873	positive regulation of cholesterol esterification	3.40E-06	6.66E-03	2.90E-04	2.36E-03	3	8	APOA2,APOA4,APOC1
GO: Biological Process	GO:0030168	platelet activation	4.17E-06	8.16E-03	3.40E-04	2.77E-03	8	233	SAA1,FGA,FGB,SERPING1,FGG,F2,TTN,F13A1
GO: Biological Process	GO:0034116	positive regulation of heterotypic cell-cell adhesion	5.09E-06	9.96E-03	3.98E-04	3.25E-03	3	9	FGA,FGB,FGG
GO: Biological Process	GO:0010872	regulation of cholesterol esterification	7.25E-06	1.42E-02	5.46E-04	4.45E-03	3	10	APOA2,APOA4,APOC1
GO: Biological Process	GO:0006928	movement of cell or subcellular component	7.89E-06	1.55E-02	5.72E-04	4.67E-03	20	1703	SPTBN4,COL6A3,SAA1,MYH9,VTN,CCDC88A,NF1,KIF27,SPTAN1,SYNE2,ACTA1,ASPM,LAMA3,LBP,F2,DNAH5,TTN,LYST,PCM1,DNAH11
GO: Biological Process	GO:0002455	humoral immune response mediated by circulating immunoglobulin	8.87E-06	1.74E-02	6.20E-04	5.06E-03	5	70	SERPING1,C8A,C9,CFI,CRP
GO: Biological Process	GO:0008203	cholesterol metabolic process	1.08E-05	2.11E-02	7.10E-04	5.79E-03	6	125	SAA1,PON1,APOA2,APOA4,APOC1,APOL1
GO: Biological Process	GO:0032374	regulation of cholesterol transport	1.13E-05	2.21E-02	7.10E-04	5.79E-03	4	35	PON1,APOA2,APOA4,APOC1
GO: Biological Process	GO:0032371	regulation of sterol transport	1.13E-05	2.21E-02	7.10E-04	5.79E-03	4	35	PON1,APOA2,APOA4,APOC1
GO: Biological Process	GO:0050727	regulation of inflammatory response	1.16E-05	2.27E-02	7.10E-04	5.79E-03	8	268	SAA1,VTN,SERPING1,LBP,C8A,C9,CFI,KLKB1
GO: Biological Process	GO:0051258	protein polymerization	1.21E-05	2.37E-02	7.18E-04	5.86E-03	7	193	SPTBN4,SPTAN1,FGA,FGB,FGG,GSN,CRP
GO: Biological Process	GO:0033700	phospholipid efflux	1.32E-05	2.59E-02	7.61E-04	6.21E-03	3	12	APOA2,APOA4,APOC1
GO: Biological Process	GO:0042157	lipoprotein metabolic process	1.41E-05	2.76E-02	7.89E-04	6.44E-03	6	131	PON1,APOA2,APOA4,APOC1,ATM,APOL1
GO: Biological Process	GO:0010038	response to metal ion	1.59E-05	3.12E-02	8.38E-04	6.84E-03	8	280	ACTA1,FGA,FGB,CP,FGG,TTN,GSN,CRP
GO: Biological Process	GO:0070613	regulation of protein processing	1.65E-05	3.22E-02	8.38E-04	6.84E-03	11	573	SERPINA3,COL6A3,SERPINA4,VTN,SERPING1,C8A,C9,CFI,F2,KLKB1,SERPIND1
GO: Biological Process	GO:0034433	steroid esterification	1.71E-05	3.35E-02	8.38E-04	6.84E-03	3	13	APOA2,APOA4,APOC1
GO: Biological Process	GO:0034435	cholesterol esterification	1.71E-05	3.35E-02	8.38E-04	6.84E-03	3	13	APOA2,APOA4,APOC1
GO: Biological Process	GO:0034434	sterol esterification	1.71E-05	3.35E-02	8.38E-04	6.84E-03	3	13	APOA2,APOA4,APOC1
GO: Biological Process	GO:0016125	sterol metabolic process	1.90E-05	3.72E-02	8.84E-04	7.21E-03	6	138	SAA1,PON1,APOA2,APOA4,APOC1,APOL1
GO: Biological Process	GO:0033344	cholesterol efflux	1.94E-05	3.80E-02	8.84E-04	7.21E-03	4	40	PON1,APOA2,APOA4,APOC1
GO: Biological Process	GO:1900047	negative regulation of hemostasis	2.14E-05	4.20E-02	8.84E-04	7.21E-03	4	41	VTN,SERPING1,F2,KLKB1
GO: Biological Process	GO:0030195	negative regulation of blood coagulation	2.14E-05	4.20E-02	8.84E-04	7.21E-03	4	41	VTN,SERPING1,F2,KLKB1
GO: Biological Process	GO:0006952	defense response	2.16E-05	4.24E-02	8.84E-04	7.21E-03	18	1515	SERPINA3,SAA1,SAA2,VTN,HP,JCHAIN,SERPING1,APOA2,APOA4,LBP,C8A,APOL1,C9,CFI,F2,KLKB1,LYST,CRP
GO: Biological Process	GO:0030193	regulation of blood coagulation	2.17E-05	4.24E-02	8.84E-04	7.21E-03	5	84	VTN,FGA,SERPING1,F2,KLKB1
GO: Biological Process	GO:1900046	regulation of hemostasis	2.17E-05	4.24E-02	8.84E-04	7.21E-03	5	84	VTN,FGA,SERPING1,F2,KLKB1
GO: Biological Process	GO:0034375	high-density lipoprotein particle remodeling	2.17E-05	4.26E-02	8.84E-04	7.21E-03	3	14	APOA2,APOA4,APOC1
GO: Biological Process	GO:0030198	extracellular matrix organization	2.25E-05	4.41E-02	8.84E-04	7.21E-03	9	385	COL6A3,VTN,NF1,FGA,FGB,LAMA3,LAMC2,FGG,KLKB1
GO: Biological Process	GO:0043062	extracellular structure organization	2.30E-05	4.50E-02	8.84E-04	7.21E-03	9	386	COL6A3,VTN,NF1,FGA,FGB,LAMA3,LAMC2,FGG,KLKB1
GO: Biological Process	GO:0040011	locomotion	2.30E-05	4.51E-02	8.84E-04	7.21E-03	18	1522	SPTBN4,COL6A3,SAA1,MYH9,VTN,CCDC88A,NF1,SPTAN1,SYNE2,ASPM,LAMA3,LBP,F2,DNAH5,TTN,LYST,SERPIND1,DNAH11
GO: Biological Process	GO:0050819	negative regulation of coagulation	2.60E-05	5.08E-02	9.78E-04	7.97E-03	4	43	VTN,SERPING1,F2,KLKB1
GO: Biological Process	GO:0050818	regulation of coagulation	2.71E-05	5.31E-02	1.00E-03	8.18E-03	5	88	VTN,FGA,SERPING1,F2,KLKB1
GO: Biological Process	GO:0007010	cytoskeleton organization	3.17E-05	6.21E-02	1.15E-03	9.38E-03	14	987	SPTBN4,MYH9,CCDC88A,NF1,GCC2,SPTAN1,SYNE2,ACTA1,NUMA1,OBSCN,ASPM,TTN,PCM1,GSN
GO: Biological Process	GO:1902041	regulation of extrinsic apoptotic signaling pathway via death domain receptors	3.40E-05	6.66E-02	1.21E-03	9.88E-03	4	46	NF1,FGA,FGB,FGG
GO: Cellular Component	GO:0072562	blood microparticle	4.76E-36	1.21E-33	1.21E-33	7.42E-33	25	132	A1BG,CFHR1,SERPINA3,VTN,HP,HPR,JCHAIN,ACTA1,FGA,FGB,PON1,SERPING1,CP,APOA2,APOA4,FGG,C8A,APOL1,C9,HBA1,F2,HBB,HBD,F13A1,GSN
GO: Cellular Component	GO:0005615	extracellular space	7.81E-25	1.99E-22	9.96E-23	6.10E-22	38	1284	A1BG,CFHR1,SERPINA3,COL6A3,SAA1,DMXL2,SAA2,SERPINA4,VTN,HP,HPR,JCHAIN,ACTA1,FGA,FGB,PON1,SERPING1,CP,LAMC2,APOA2,APOA4,APOC1,LBP,FGG,C8A,APOL1,C9,HBA1,CFI,F2,HBB,HBD,KLKB1,LRG1,SERPIND1,F13A1,GSN,CRP
GO: Cellular Component	GO:0034364	high-density lipoprotein particle	1.24E-13	3.16E-11	1.05E-11	6.45E-11	8	28	SAA1,SAA2,HPR,PON1,APOA2,APOA4,APOC1,APOL1
GO: Cellular Component	GO:0034358	plasma lipoprotein particle	2.95E-12	7.53E-10	1.88E-10	1.15E-09	8	40	SAA1,SAA2,HPR,PON1,APOA2,APOA4,APOC1,APOL1
GO: Cellular Component	GO:0032994	protein-lipid complex	4.50E-12	1.15E-09	2.30E-10	1.41E-09	8	42	SAA1,SAA2,HPR,PON1,APOA2,APOA4,APOC1,APOL1
GO: Cellular Component	GO:0060205	cytoplasmic membrane-bounded vesicle lumen	8.85E-10	2.26E-07	3.76E-08	2.30E-07	8	79	SAA1,HP,FGA,FGB,SERPING1,FGG,HBB,F13A1
GO: Cellular Component	GO:0031983	vesicle lumen	1.20E-09	3.05E-07	4.36E-08	2.67E-07	8	82	SAA1,HP,FGA,FGB,SERPING1,FGG,HBB,F13A1
GO: Cellular Component	GO:0031838	haptoglobin-hemoglobin complex	2.41E-07	6.14E-05	7.67E-06	4.70E-05	3	4	HP,HBA1,HBB
GO: Cellular Component	GO:0034385	triglyceride-rich lipoprotein particle	1.06E-06	2.71E-04	2.71E-05	1.66E-04	4	20	APOA2,APOA4,APOC1,APOL1
GO: Cellular Component	GO:0034361	very-low-density lipoprotein particle	1.06E-06	2.71E-04	2.71E-05	1.66E-04	4	20	APOA2,APOA4,APOC1,APOL1
GO: Cellular Component	GO:0031093	platelet alpha granule lumen	1.30E-06	3.32E-04	3.01E-05	1.84E-04	5	48	FGA,FGB,SERPING1,FGG,F13A1
GO: Cellular Component	GO:0005856	cytoskeleton	1.59E-06	4.05E-04	3.38E-05	2.07E-04	23	1993	SPTBN4,BPTF,MYH9,NF1,MDN1,KIF27,MYO5B,SPTAN1,CNTRL,SYNE2,ACTA1,NUMA1,OBSCN,ASPM,EVPL,ATM,PIBF1,DNAH5,TTN,LYST,PCM1,GSN,DNAH11
GO: Cellular Component	GO:0034366	spherical high-density lipoprotein particle	3.33E-06	8.50E-04	6.07E-05	3.71E-04	3	8	HPR,PON1,APOA2
GO: Cellular Component	GO:0005577	fibrinogen complex	3.33E-06	8.50E-04	6.07E-05	3.71E-04	3	8	FGA,FGB,FGG
GO: Cellular Component	GO:0031091	platelet alpha granule	4.34E-06	1.11E-03	7.38E-05	4.51E-04	5	61	FGA,FGB,SERPING1,FGG,F13A1
GO: Cellular Component	GO:0034774	secretory granule lumen	4.71E-06	1.20E-03	7.50E-05	4.59E-04	5	62	FGA,FGB,SERPING1,FGG,F13A1
GO: Cellular Component	GO:0042627	chylomicron	1.29E-05	3.30E-03	1.83E-04	1.12E-03	3	12	APOA2,APOA4,APOC1
GO: Cellular Component	GO:0005833	hemoglobin complex	1.29E-05	3.30E-03	1.83E-04	1.12E-03	3	12	HBA1,HBB,HBD
GO: Cellular Component	GO:0071682	endocytic vesicle lumen	3.26E-05	8.30E-03	4.37E-04	2.68E-03	3	16	SAA1,HP,HBB
GO: Cellular Component	GO:0005938	cell cortex	3.59E-05	9.15E-03	4.57E-04	2.80E-03	7	230	SPTBN4,MYH9,SPTAN1,FGA,FGB,LAMC2,FGG
GO: Cellular Component	GO:0044433	cytoplasmic vesicle part	4.32E-05	1.10E-02	5.25E-04	3.21E-03	10	527	SAA1,DMXL2,MYO5B,HP,FGA,FGB,SERPING1,FGG,HBB,F13A1
GO: Cellular Component	GO:0044430	cytoskeletal part	5.35E-05	1.36E-02	6.20E-04	3.80E-03	17	1477	SPTBN4,MYH9,NF1,KIF27,MYO5B,SPTAN1,CNTRL,ACTA1,NUMA1,OBSCN,ASPM,ATM,PIBF1,DNAH5,TTN,PCM1,DNAH11
GO: Cellular Component	GO:0005610	laminin-5 complex	9.31E-05	2.37E-02	1.03E-03	6.32E-03	2	4	LAMA3,LAMC2
GO: Cellular Component	GO:0036379	myofilament	1.01E-04	2.57E-02	1.07E-03	6.56E-03	3	23	ACTA1,OBSCN,TTN
GO: Cellular Component	GO:0015630	microtubule cytoskeleton	1.07E-04	2.72E-02	1.09E-03	6.65E-03	13	971	BPTF,MYH9,KIF27,SPTAN1,CNTRL,NUMA1,ASPM,ATM,PIBF1,DNAH5,LYST,PCM1,DNAH11
GO: Cellular Component	GO:0016459	myosin complex	1.30E-04	3.31E-02	1.27E-03	7.79E-03	4	65	MYH9,MYO5B,OBSCN,TTN
GO: Cellular Component	GO:0016460	myosin II complex	1.65E-04	4.20E-02	1.56E-03	9.53E-03	3	27	MYH9,OBSCN,TTN

Table S3. IPA-generated functional interaction network based on scores for septic patients.

ID	Molecules in Network	Score	Focus Molecules	Top Diseases and Functions
IPA generated functional interaction network with based of score for septic survivor patients after day 0 (D0S). Green down arrow represent down-regulated proteins and red up arrow represent up-regulation. The bold symbols are the proteins, identified in the present study.				
1	↓**APOA2**, ↓**APOA4**, ↓**APOC1**, ↓**APOC2**, ↓**APOC3**, ↓**APOD**, ↑**CPN1**, ↑**CRP**, ERK1/2, ↑**FGA**, ↑**FGB**, ↑ **FGG**, Fibrin, Fibrinogen, GPIIB-IIIA, HDL, HDL-cholesterol, hemoglobin, ↑**HP**, ↑**HPR**, ↑**LAMA3**, ↑**LBP**, LDL-cholesterol, ↑**MYH13**, NCOR-LXR-Oxysterol-RXR-9 cis RA, Nrlh, ↑**ORM1**, ↑**ORM2**, ↓**PON**, SAA, ↑**SAAI**, ↑**SAA2**, ↑**SERPINA1**, Stat3-Stat3, VLDL-cholesterol	48	22	Metabolic Disease, Lipid Metabolism, Small Molecule Biochemistry
2	Actin, Akt, Alpha catenin, calpain, caspase, chymotrypsin, ↑**COL6A3**, Collagen(s), ↓**F2**, F Actin, ↓**GSN**, ↓**KLKBI**, Laminin, ↑**LRG1**, MIc, ↑**MYH9**, ↓**MYO5A**, ↑**MYO5B**. ↑**MYO7A**, Myosin, NADPH oxidase, ↑**OBSCN**, PDGF BB, ↓**PLEC**, SerineProtease, ↑**SERPINA3**, ↓**SERPIND1**, Sos, ↓**SPTAN1**, ↓**SPTBN4**, ↑**SYNE1**, T3-TR-RXR, Tgf beta, trypsin, ↑**TTN**	33	17	Cellular Assembly and Organization, Cellular Function and Maintenance, Cellular Movement
3	AHR, ↑**ALMS1**, ↑**ASPM**, Ca2+, CBX4, ↑**DMXL2**, DNAH1, DNAH2. DNAH3, ↑**DNAH5**, DNAH6, ↑**DNAH8**, DNAH9, ↑**DNAH10**, ↑**DNAH11**, ↑**DNAH12**, DNAH14, ↓**DNAH17**, DNAI1, DNAI2, DNAL1, DNAL4, DNAL1I, ↑**EVPL**, FPR2, ↓**GCC2**, IDH3G, MYC, RFX2. RFX3, ↑**RNF2I3**, SP1, ↑**SVEP1**, TGFB1, ↓**TRANKI**	26	13	Developmental Disorder, Hereditary Disorder, Respiratory Disease
IPA generated functional interaction network with based of score for septic non-survivors patients after day 0 (D0NS).				
1	↓**APOA2**, ↓**APOA4**, ↓**APOC1**, ↓**APOC2**, ↓**APOC3**, ↑**CRP**, ERK1/2, ↑**FGA**, ↑**FGB**, ↑**FGG**, Fibrin, Fibrinogen, GPIIB-IIIA, Growth hormone, HDL, HDL-cholesterol, hemoglobin, ↑**HP**, ↑**HPR**, ↑**LAMA3**, ↑**LBP**, IDL-cholesteroL NCOR-LXR-Oxysterol-RXR-9 cis RA, ↑**ORM1**, ↑**ORM2**, ↓**PON**, Pro-inflammatory Cytokine, ↑**PROS1**, ↑**SAAI**, ↑**SAA2**, ↑**SERPINA1**, Stat3-Stat3, ↓**TTR**, VLDL-cholesterol	45	21	Metabolic Disease, lipid Metabolism, Molecular Transport
2	↑**ACTB**, Actin, ↓**AFM**, Akt, ↑**ALMS1**, Alpha catenin, ↓**ANK3**, caspase, chymotrypsin, ↑**COL6A3**, Collagen(s), ↓**DYSF**, ↓**F2**, F Actin, ↓**GSN**, ↑**HBA1/HBA2**, ↑**HBB**, ↑**HBD**, lgG, Laminin, ↑**LRG1**, MIc, ↑**MYH9**, ↑**MYO5B**, NADPH oxidase. PId, Serine Protease, ↑**SERPINA3**, ↓**SERPINDI**, ↓**SHROOM3**, Sos, ↓**SPTAN1**, ↑**SYNE2**, T3-TR-RXR,TCF	38	19	Cell-to-Cell Signaling and Interaction, Cellular Assembly and Organization, Connective Tissue Disorders
3	↑**ASPM**, beta-estradiol, Ca2+, CD3, ↑**DMXL2**, DNAH2, DNAH3, ↑**DNAH5**, DNAH6, ↑**DNAH8**, DNAH9, ↑**DNAH10**, ↑**DNAH11**, ↑**DNAH12**, DNAH14, ↓**DNAH17**, DNAI1, DNAI2, DNAL1, DNALI1, ↓**DST**, ↑**EVPL**, FCN1, FPR2, ↓**GCC2**, ↑**GOLGB1**, GPR65, IFNG, 1L13, ↑**MDN1**, MYC, ↓**PIBFI**, S100A12, ↑**SVEPI**, TGFB1	28	14	Developmental Disorder, Hereditary Disorder, Respiratory Disease
IPA generated functional interaction network with based of score for septic survivors patients after day 7 (D7S).				
1	↓**APOA2**, ↓**APOA4**, ↓**APOC1**, ↑**CLU**, ↑**CP** ↑**CRP**, ERK1/2, Ferritin, ↑**FGA**, ↑**FGB**, ↑**FGG**, Fibrin, Fibrinogen, GPIIB-IIIA, ↑**HBA1/HBA2**, HDL, HDL-cholesterol, hemoglobin, ↑**HP**, ↑**HPR**, ↑**HPX**, ↑**LAMA3**, ↑**LBP**, LDL, LDL-cholesterol, NADPH oxidase, ↑**ORM1**, ↓**PON**, Pro-inflammatory Cytokine, ↑**SAA1**, ↑**SAA2**, ↑**SERPINA1**, Stat3-Stat3, ↑**TRMP2**, VLDL-cholesterol	43	21	Metabolic Disease, Cardiovascular Disease, Developmental Disorder
2	Ap1, ↑**ATM**, ATPase, caspase, CD3, Cg, Creb, ↑**DNAH5**, ↓**DNAH10**, ↑**DNAH11**, ↑**GOLGB1**, ↑**HBB**, ↑**HBD**, HISTONE, Histoneh3, Histoneh4, ↓**HLTF**, Hsp9O, lgG, IL1, Immunoglobulin, ↑**JCHAIN**, ↑**KMT2C**, Mapk, Mek, NFkB (complex), ↑**nikotinamide adenine dinucleotide phosphate**, ↑**NIPBL**, RNA polymerase II, ↑**RNF213**, ↑**SACS**, ↑**SBF1**, ↑**SRCAP**, Tgf beta, Vegf	32	16	Cancer, Organismal Injury arid Abnormalities, Endocrine System Disorders
3	↑**ACTAl**, ↑**ACTB**, Actin, Akt, Alpha Actinin, Alpha catenin, Calmodulin, chymotrypsin, Cofilin, Collagen(s), Dynein, F Actin, G-Actin, ↓**GSN**, ↑**LAMA4**, Laminin, ↑**LRGl**, MIc, ↑**MYH9**, ↑**MYO5B**, ↑**MYO7A**, Myosin, ↑**NFI**, ↑**OBSCN**, ↑**PCNT**, PDGF-BB, PId, Rock, Serine Protease, ↑**SERPINA3**, ↓**SERPINDI**, ↓**SPTBN4**, ↑**SYNE2**, trypsin, ↑**TTN**	30	16	Cellular Assembly and Organization, Cellular Development, Cellular Growth and Proliferation
IPA generated functional interaction network with based of score for septic non-survivors patients after day 7 (D7NS).				
1	↑**A1BG**, ↓**APOA2**, ↓**APOA4**, ↓**APOC1**, ↓**APOLI**, ↓**CCDC88A**, ↑**CRP**, ERK1/2, ↓**F13A1**, factor XIII, Ferritin, ↑**FGA**, ↑**FGB**, ↑**FGG**, Fibrin, Fibrinogen, GPIIB-IIIA, HDL, HDL-cholesterol, hemoglobin, ↑**HP**, ↑**HPR**, ↑**LAMA3**, ↓**LAMC2**, ↑**LBP**, LDL-cholesterol, MHC Class II (complex), ↓**PON1**, Pro inflammatory Cytokine, ↑**SAA1**, ↑**SAA2**, Stat3-Stat3, ↑**TRPM2**, VLDL-cholesterol, ↓**VTN**	43	21	Developmental Disorder, Hematological Disease, Hereditary Disorder
2	↑**ACTA1**, Actin, Akt, Alpha catenin, caspase, chymotrypsan, ↑**COL6A3**, Collagen(s), ↓**F2**, F Actin, ↓**GSN**, ↑**HBAI/HBA2**, ↑**HBB**, ↑**HBD**, ↓**HLTF**, Kallikrein, ↓**KLKB1**, Laminin, ↑**LRGI**, MIc, ↑**MYH9**, ↑**MYO5B**, Myosin, NADPH oxidase, ↑**OBSCN**, PId, ↑**SERPINA3**, ↓**SFRPINA4**, ↓**SERPIND1**, ↓**SPTANI**, ↓**SPTBN4**, ↑**SYNE2**, T3-TR-RXR, trypsin, ↑**TTN**	39	20	Cellular Assembly and Organization, Cellular Development, Cellular Growth and Proliferation
3	APP, ↑**ASPM**, BET1L, ↑**BODIL1**, ↓**BPTF**, ↓**C8A**, ↑**CFHR2**, ↑**CNTRL**, DECR2, DNAH2, ↑**DNAH5**, DNAH6, ↓**DNAH10**, DNAH14, DNALI1, DYNLT1, ↑**EVPL**, ↓**GCC2**, ↑**GOLGA4**, ↑**GOLGB1**, GOSR2, ↑**MDN1**, P2RX6, PARVA, ↓**PIBFI**, POU5F1, PPARA, SGK2, SOX2, ↑**SVEPI**, TGFB1, TLL1, TNFRSP1A, XBP1, ↓**ZFHX4**	29	15	Cellular Development, Tissue Development, Cancer

Table S4. Altered functions with their activation and inhibition z scores in septic patients.

S. No.	Categories	Diseases or Functions Annotation	p-Value	Predicted Activation State	Activation z-score	# Molecules
Functional interaction network annotation of differentially expressed proteins in septic survival patients at day 0 (D0S) in order to Z-scores.						
1	Lipid Metabolism, Molecular Transport, Small Molecule Biochemistry	transport of phospholipid	1.47E-07	Decreased	-2.236	6
2	Lipid Metabolism, Molecular Transport, Small Molecule Biochemistry	efflux of phospholipid	5.60E-08	Decreased	-2	5
3	Cardiac Hypertrophy, Cardiovascular Disease, Developmental Disorder, Organismal Injury and Abnormalities	hypertrophy of heart	3.82E-02		-1.977	4
4	Molecular Transport	export of molecule	3.46E-05		-1.703	8
5	Lipid Metabolism, Molecular Transport, Small Molecule Biochemistry	quantity of steroid	1.35E-04		-1.509	9
6	Lipid Metabolism, Molecular Transport, Small Molecule Biochemistry	efflux of cholesterol	2.13E-07		-1.429	7
7	Lipid Metabolism, Molecular Transport, Small Molecule Biochemistry	transport of lipid	5.28E-08		-1.336	10
8	Lipid Metabolism, Molecular Transport, Small Molecule Biochemistry	concentration of cholesterol	1.45E-04		-1.298	7
9	Lipid Metabolism, Molecular Transport, Small Molecule Biochemistry	concentration of fatty acid	1.07E-03		-1.266	6
10	Lipid Metabolism, Molecular Transport, Small Molecule Biochemistry	transport of steroid	1.43E-07		-1.254	8
11	Cardiovascular System Development and Function, Cell-To-Cell Signaling and Interaction	activation of vascular endothelial cells	2.58E-06		-1.217	4
12	Lipid Metabolism, Molecular Transport, Small Molecule Biochemistry	concentration of sterol	2.98E-05		-1.199	8
13	Lipid Metabolism, Small Molecule Biochemistry	fatty acid metabolism	6.10E-05		-1.17	11
14	Lipid Metabolism, Molecular Transport, Small Molecule Biochemistry	concentration of lipid	4.70E-05		-1.04	13
15	Cellular Movement, Hematological System Development and Function, Immune Cell Trafficking, Inflammatory Response	chemotaxis of mononuclear leukocytes	5.49E-04		-1	5
16	Organismal Injury and Abnormalities	Bleeding	4.03E-06		-0.95	10
17	Cellular Movement	chemotaxis of cells	2.56E-04		-0.931	9
18	Protein Synthesis	quantity of protein lipid complex in blood	6.34E-06		-0.931	5
19	Cardiovascular System Development and Function, Cellular Development, Cellular Growth and Proliferation, Organismal Development, Tissue Development	proliferation of endothelial cells	1.30E-03		-0.842	6
20	Cellular Movement, Hematological System Development and Function, Immune Cell Trafficking	cell movement of leukocytes	3.75E-04		-0.836	11
21	Cardiovascular Disease	atherosclerosis	2.86E-09		-0.695	14
22	Cardiovascular System Development and Function, Cell-To-Cell Signaling and Interaction	activation of endothelial cells	2.72E-06		-0.69	5
23	Free Radical Scavenging	metabolism of reactive oxygen species	2.88E-03		-0.62	8
24	Molecular Transport	transport of molecule	1.20E-05		-0.555	20
25	Lipid Metabolism, Small Molecule Biochemistry	synthesis of fatty acid	2.46E-03		-0.544	6
26	Cellular Movement, Hematological System Development and Function, Immune Cell Trafficking, Inflammatory Response	cell movement of phagocytes	4.25E-04		-0.543	9
27	Cellular Movement, Hematological System Development and Function, Immune Cell Trafficking	cell movement of myeloid cells	4.02E-04		-0.529	9
28	Cell-To-Cell Signaling and Interaction, Hematological System Development and Function, Immune Cell Trafficking, Inflammatory Response	activation of myeloid cells	4.94E-04		-0.439	7
29	Cell-To-Cell Signaling and Interaction, Hematological System Development and Function, Immune Cell Trafficking, Inflammatory Response	activation of phagocytes	7.76E-04		-0.439	7
30	Inflammatory Response	inflammatory response	3.73E-06		-0.429	14
31	Cellular Movement, Hematological System Development and Function, Immune Cell Trafficking, Inflammatory Response	cell movement of neutrophils	1.05E-03		-0.391	6
32	Cell-To-Cell Signaling and Interaction	adhesion of blood cells	3.26E-06		-0.381	10
33	Cellular Movement	cell movement	1.09E-06		-0.372	25
34	Cell-To-Cell Signaling and Interaction	aggregation of cells	7.73E-06		-0.353	9
35	Molecular Transport	secretion of molecule	9.99E-05		-0.343	10
36	Cellular Movement, Hematological System Development and Function, Immune Cell Trafficking, Inflammatory Response	chemotaxis of phagocytes	1.42E-05		-0.254	8
37	Cell-To-Cell Signaling and Interaction, Hematological System Development and Function, Immune Cell Trafficking	adhesion of immune cells	1.05E-05		-0.253	9
38	Cardiovascular Disease	vascular lesion	1.76E-05		-0.243	7
39	Cell-To-Cell Signaling and Interaction, Hematological System Development and Function, Immune Cell Trafficking, Inflammatory Response	adhesion of phagocytes	1.74E-03		-0.205	4
40	Cellular Movement, Hematological System Development and Function, Immune Cell Trafficking, Inflammatory Response	chemotaxis of myeloid cells	1.37E-05		-0.128	8
41	Cellular Assembly and Organization, Cellular Function and Maintenance	organization of cytoskeleton	2.35E-06		-0.055	19
42	Cellular Assembly and Organization, Cellular Function and Maintenance	microtubule dynamics	4.21E-06		0.116	17
43	Cardiovascular Disease	atherosclerotic lesion	1.60E-05		0.13	6
44	Molecular Transport	quantity of metal	1.93E-03		0.156	8
45	Hematological System Development and Function	hemostasis	2.21E-05		0.191	7
46	Hematological System Development and Function	coagulation	9.54E-05		0.192	6
47	Cell-To-Cell Signaling and Interaction, Hematological System Development and Function, Inflammatory Response	aggregation of blood platelets	1.20E-06		0.335	8
48	Cardiovascular Disease, Hematological Disease	Thrombosis	3.09E-04		0.613	5
49	Cellular Assembly and Organization	quantity of filaments	2.45E-07		0.655	7
50	Cell-To-Cell Signaling and Interaction, Cellular Function and Maintenance, Inflammatory Response	phagocytosis of cells	6.75E-04		0.676	6
51	Cellular Function and Maintenance, Inflammatory Response	phagocytosis	1.71E-04		0.961	7

52	Cellular Function and Maintenance	engulfment of cells	1.23E-03		1.02	7
53	Lipid Metabolism, Molecular Transport, Small Molecule Biochemistry	uptake of lipid	5.49E-05		1.387	6

Functional interaction network annotation of differentially expressed proteins in septic nonsurvival patients at day 0 (D0NS) in order to Z-scores.

1	Lipid Metabolism, Molecular Transport, Small Molecule Biochemistry	transport of phospholipid	1.77E-07	Decreased	-2.236	6
2	Cellular Assembly and Organization, Tissue Development	fibrogenesis	3.288-05	Decreased	-2.17	9
3	Lipid Metabolism, Molecular Transport. Small Molecule Biochemistry	efflux of phospholipid	6.55E-08	Decreased	-2	5
4	Molecular Transport	export of molecule	5.41 E-06		-1.953	9
5	Lipid Metabolism, Molecular Transport, Small Molecule Biochemistry	concentration of lipid	2.76E-04		-1.688	12
6	Lipid Metabolism, Molecular Transport. Small Molecule Biochemistry	quantity of steroid	1.72E-04		-1.509	9
7	Lipid Metabolism, Molecular Transport, Small Molecule Biochemistry	efflux of cholesterol	2.64E-07		-1.429	7
8	Lipid Metabolism, Small Molecule Biochemistry	fatty acid metabolism	8.17E-05		-1.369	11
9	Lipid Metabolism, Molecular Transport, Small Molecule Biochemistry	transport of lipid	7.16E-08		-1.336	10
10	Lipid Metabolism, Molecular Transport, Small Molecule Biochemistry	concentration of cholesterol	1.76E-04		-1.298	7
11	Molecular Transport	transport of molecule	5.30E-06		-1.271	21
12	Lipid Metabolism, Molecular Transport, Small Molecule Biochemistry	transport of steroid	1.83E-07		-1.254	8
13	Free Radical Scavenging	metabolism of reactive oxygen species	3.64E-05		-1.246	11
14	Free Radical Scavenging	synthesis of reactive oxygen species	1.39E-04		-1.246	10
15	Tissue Development	development of epithelial tissue	1.22E-03		-1.234	8
16	Cardiovascular System Development and Function, Cellular Development, Cellular Growth and Proliferation, Organismal Development, Tissue Development	proliferation of endothelial cells	2.42E-04		-1.234	7
17	Lipid Metabolism, Molecular Transport, Small Molecule Biochemistry	concentration of sterol	3.74E-05		-1.199	8
18	Cardiovascular System Development and Function, Cellular Development, Cellular Growth and Proliferation, Organismal Development, Tissue Development	proliferation of vascular endothilial	1.91 E-03		-1.154	4
19	Free Radical Scavenging	production of reactive oxygen species	4.24E-04		-1.056	8
20	Protein Synthesis	quantity of protein lipid concentration	7.38E-06		-0.931	5
21	Lipid Metabolism, Small Molecule Biochemistry	hydrolysis of lipid	1.188-03		-0.865	5
22	Lipid Metabolism, Small Molecule Biochemistry	synthesis of fatty acid	2.88E-03		-0.84	6
23	Lipid Metabolism, Small Molecule Biochemistry	synthesis of lipid	5.36E-03		-0.717	9
24	Cardiovascular Disease	atherosclerosis	4.39E-09		-0,695	14
25	Cellular Movement	chemotaxis of cells	1.52E-03		-0.657	8
26	Cellular Movement, Hematological System Development and Function, Immune C	cell movement of phagocyt	2.34E-03		-0.543	8
27	Cellular Movement, Hematological System Development and Function, Immune C	cell movement of myeloid c	2.23E-03		-0.529	8
28	Cellular Movement. Hematological System Development and Function, Immune C	cell movement of neutrophil	1.23E-03		-0.391	6
29	Cell-To-Cell Signaling and Interaction	aggregation of cells	1.19E-06		-0.353	10
30	Molecular Transport	secretion of molecule	1.30E-04		-0.343	10
31	Cellular Assembly and Organization	quantity of filaments	1.03E-03		-0.277	4
32	Cellular Movement. Hematological System Development and Function, Immune C	chemotaxis of phagocytes	1.43E-04		-0.254	7
33	Cell-To-Cell Signaling and Interaction	binding of cells	1.11E-03		-0.245	8
34	Cardiovascular Disease	vascular lesion	2.16E-05		-0.243	7
35	Cellular Movement. Hematological System Development and Function, Immune C	cell movement of leukocyte	4.93E-04		-0.239	11
36	Cell-To-Cell Signaling and Interaction, Hematological System Development and Fu	adhesion of phagocytes	1.95E-03		-0.205	4
37	Cardiovascular System Development and Function, Cell-To-Cell Signaling and Inte	activation of endothelial cell	7.88E-05		-0.174	4
38	Cell-To-Cell Signaling and Interaction	adhesion of blood cells	3.14E-05		-0.143	9
39	Cellular Movement. Hematological System Development and Function, Immune C	chemotaxis of myeloid cells	1.38E-04		-0.128	7
40	Cardiovascular System Development and Function, Organismal Development	vasculogenesis	2.56E-04		-0.1	11
41	Cell-To-Cell Signaling and Interaction	activation of cells	4.11E-03		-0.071	11
42	Cell-To-Cell Signaling and Interaction, Hematological System Development and Fu	aggregation of blood platelets	1.18E-07		-0.028	9
43	Cell-To-Cell Signaling and Interaction, Hematological System Development and Fu	adhesion of immune cells	9.67E-05		-0.008	8
44	Cardiovascular Disease	atherosclerotic lesion	1.91E-05		0.13	6
45	Cardiovascular System Development and Function, Organismal Development	angiogenesis	4.28E-04		0.227	12
46	Molecular Transport	quantity of metal	1.10E-04		0.228	10
47	Cellular Movement	cell movement	7.75E-06		0.228	24
48	Cell-To-Cell Signaling and Interaction, Hematological System Development and Fu	activation of macrophages	2.60E-03		0.294	5
49	Cell Signaling. Molecular Transport, Vitamin and Mineral Metabolism	quantity of Ca2+	6.91E-04		0.506	8
50	Hematological System Development and Function	hemeostasis	2.70E-05		0.564	7
51	Hematological System Development and Function	coagulation	1.13E-04		0.565	6
52	Cell-To-Cell Signaling and Interaction, Hematological System Development and Fu	activation of mycloid cells	3.23E-03		0.596	6
53	Cell-To-Cell Signaling and Interaction, Hematological System Development and Fu	activation of phagocytes	4.68E-03		0.596	6
54	Inflammatory Response	inflammatory response	1.25E-02		0.619	12
55	Cell-To-Cell Signaling and Interaction, Cellular Function and Maintenance, Inflamr phagocytosis of cells	Phagocytosis of cells	7.95E-04		0.676	6
56	Cellular Function and Maintenance engulfment of cells	engulfment of cells	1.48E-03		1.02	7
57	Lipid Metabolism, Molecular Transport. Small Molecule Biochemistry	uptake of lipid	6.54E-05		1.387	6

Functional interaction network annotation of differentially expressed proteins in septic survival patients after day 7 (D7S) in order to Z-scores.

1	Organismal Injury and Abnormalities	Fibrosis	8.61E-04	Decreased	-2.213	9
2	Cardiac Hypertrophy, Cardiovascular Disease, Developmental Disorder, Organismal Injury and Abnormalities	hypeitrophy of heart	3.99E-03	Decreased	-2.211	6
3	Organismal Injury and Abnormalities	Bleeding	1.17E-04		-1.687	9

4	Lipid Metabolism, Molecular Transport, Small Molecule Biochemistry	concentration of sterol	1.04E-04	-1.431	8
5	Free Radical Scavenging	metabolism of reactive oxygen species	2.85E-05	-1.167	12
6	Free Radical Scavenging	synthesis of reactive oxygen species	4.51E-04	-1.167	10
7	Free Radical Scavenging	production of reactive oxygen species	1.10E-03	-0.968	8
8	Protein Synthesis	quantity of protein lipid complex in blood	1.48E-05	-0.931	5
9	Lipid Metabolism, Small Molecule Biochemistiy	fatty acid metabolism	1.19E-03	-0.886	10
10	Cell-To-Cell Signaling and Interaction, Hernatological System Development and Function, Immune Cell Trafficking, Inflammatory Response. Nervous System	activation of microglia	1.07E-03	-0.817	4
11	Molecular Transport	quantity of metal	3.62E-04	-0.644	10
12	Lipid Metabolism, Molecular Transport, Small Molecule Biochemistry	transport of steroid	7.11E-06	-0.516	7
13	Cell-To-Cell Signaling and Interaction, Hernatological System Development and Function, Inflammatory Response	aggregation of blood platelets	3.30E-08	-0.484	10
14	Lipid Metabolism, Molecular Transport. Small Molecule Biochemistry	transport of lipid	2.76E-06	-0.447	9
15	Tissue Development	development of epithehal tissue	3.02E-03	-0.416	8
16	Cardiovascular Disease	atherosclerosis	3.55E-09	-0.416	15
17	Lipid Metabolism, Molecular Transport, Small Molecule Biochemistry	efflux of cholesterol	1.11E-05	-0.415	6
18	Cardiovascular Disease, Hematological Disease	Thrombosis	6.84E-04	-0.343	5
19	Cardiovascular Disease	vascular lesion	5.44E-05	-0.243	7
20	Cellular Movement, Hematological System Development and Function Immune Cell Trafficking, Inflammatory Response	cell movement of phagocytes	1.50E-03	-0.227	9
21	Cellular Movement, Hematological System Development and Function, Immune Cell Trafficking	cell movement of myeloid cells	1.43E-03	-0.211	9
22	Molecular Transport	export of molecule	1.20E-04	-0.183	8
23	Cellular Movement, Hair and Skin Development and Function	cell movement of epithelial cell lines	1.01E-03	-0.152	4
24	Neurological Disease	damage of central nervous system	2.03E-03	-0.152	5
25	Cell-To-Cell Signaling and Interaction, Hematological System Development and Function	aggregation of blood cells	2.36E-08	-0.023	11
26	Cellular Movement, Hematological System Development and Function, Immune Cell Trafficking. Inflammatory Response	cell movement of neutrophils	4.50E-04	0	7
27	Cellular Movement, Hematological System Development and Function, Immune Cell Trafficking	cell movement of leukocytes	4.64E-04	0.026	12
28	Cell-To-Cell Signaling and Interaction, Hematological System Development and Function, Immune Cell Trafficking, Inflammatory Response	activation of macrophages	7.93E-04	0.104	6
29	Cell-To-Cell Signaling and Interaction	aggregation of cells	6.55E-09	0.112	13
30	Cellular Movement, Immune Cell Trafficking	leukocyte migration	4.45E-04	0.127	13
31	Cardiovascular Disease	atherosclerotic lesion	4.29E-03	0.13	6
32	Cardiovascular System Development and Function, Organismal Development	angiogenesis	4.65E-04	0.248	13
33	Cellular Movement	cell movement	1.03E-06	0.364	28
34	Cell-To-Cell Signaling and Interaction, Hematological System Development and Function, Immune Cell Trafficking. Inflammatory Response	activation of myeloid cells	1.39E-03	0.392	7
35	Inflammatory Response	inflammatory response	1.27E-02	0.482	13
36	Molecular Transport	transport of molecule	5.00E-06	0.739	23
37	Cell-To-Cell Signaling and Interaction	adhesion of blood cells	1.58E-05	0.83	10
38	Cell-To-Cell Signaling and Interaction, Hematological System Development and Function, Immune Cell Trafficking	adhesion of immune cells	4.30E-05	0.972	9
39	Organismal Survival	lifespan of organism	2.59E-03	1.067	4
40	Cellular Assembly and Organization	quantity of filaments	1.63E-04	1.213	5
41	Cellular Movement	migration of cells	5.16E-05	1.219	23
42	Organismal Injury and Abnormalities, Tissue Morphology	size of lesion	1.38E-04	1.342	7
Functional interaction network annotation of differentially expressed proteins in septic nonsurvival patients after day 7 (D7NS) in order to Z-scores					
1	Cardiac Hypertrophy, Cardiovascular Disease, Developmental Disorder, Organismal Injury and Abnormalities	hypertrophy of heart	5.25E-02	-1.977	4
2	Cellular Movement	chemotaxis of cells	1.16E-04	-1.612	10
3	Lipid Metabolism, Molecular Transport, Small Molecule Biochemistry	quantity of steroid	1.42E-03	-1.509	8
4	Lipid Metabolism, Small Molecule Biochemistry	fatty acid metabolism	6.93E-04	-1.37	10
5	Cellular Movement, Hematological System Development and Function, Immune Cell Trafficking, Inflammatory Response	chemotaxis of leukocytes	2.10E-05	-1.299	9
6	Cellular Movement, Hematological System Development and Function, Immune Cell Trafficking, Inflammatory Response	chemotaxis of phagocytes	3.08E-05	-1.299	8
7	Lipid Metabolism, Molecular Transport, Small Molecule Biochemistry	concentration of cholesterol	2.78E-04	-1.298	7
8	Cellular Movement, Hematological System Development and Function, Immune Cell Trafficking. Inflammatory Response	chemotaxis of myeloid cells	2.97E-05	-1.254	8
9	Cardiovascular System Development and Function, Cell-To-Cell Signaling and Interaction	activation of vascular endothehal cells	3.91E-06	-1.217	4
10	Cell-To-Cell Signaling and Interaction, Hematological System Development and Function, Immune Cell Trafficking, Inflammatory Response. Nervous system	activation of microglia	6.35E-05	-1.216	5
11	Cellular Assembly and Organization, Tissue Development	fibrogenesis	5.92E-05	-1211	9
12	Molecular Transport	export of molecule	2.68E-03	-1.145	6
13	Free Radical Scavenging	metabolism of reactive oxygen species	3.44E-04	-1.053	10
14	Free Radical Scavenging	synthesis of reactive oxygen species	1.13E-03	-1.053	9
15	Cellular Assembly and Organization	quantity of actin filaments	4.87E-06	-1	5

16	Lipid Metabolism, Molecular Transport, Small Molecule Biochemistry	concentration of lipid	1.94E-03	-0.998	11
17	Free Radical Scavenging	production of reactive oxygen species	3.23E-03	-0.968	7
18	Lipid Metabolism, Molecular Transport, Small Molecule Biochemistry	transport of steroid	5.34E-05	-0.952	6
19	Protein Synthesis	quantity of protein lipid complex in blood	1.06E-05	-0.931	5
20	Lipid Metabolism, Molecular Transport, Small Molecule Biochemistry	transport of lipid	1.44E-05	-0.915	8
21	Lipid Metabolism, Molecular Transport, Small Molecule Biochemistry	efflux of cholesterol	1.07E-04	-0.875	5
22	Cardiovascular System Development and Function, Cellular Development, Cellular Growth and Proliferation, Organismal Development. Tissue Devlopment	proliferation of endothelial cells	2.22E-03	-0.842	6
23	Organismal Injury and Abnormalities	Bleeding	6.82E-05	-0.833	9
24	Cardiovascular Disease	atherosclerosis	1.30E-09	-0.695	15
25	Inflammatory Response	inflammatory response	2.69E-06	-0.616	15
26	Cell-To-Cell Signaling and Interaction, Hematological System Development and Function, Immune Cell Trafficking, Inflammatory Response	activation of myeloid cells	2.44E-05	-0.393	9
27	Cell-To-Cell Signaling and Interaction, Hematological System Development and Function, Immune Cell Trafficking, Inflammatory Response	activation of phagocytes	4.42E-05	-0.393	9
28	Cardiovascular System Development and Function, Cell-To-Cell Signaling and Interaction	activation of endothelial cells	1.61E-07	-0.355	6
29	Cell-To-Cell Signaling and Interaction, Hematological System Development and Function, Immune Cell Trafficking, Inflammatory Response	activation of macrophages	7.20E-05	-0.354	7
30	Cell-To-Cell Signaling and Interaction	aggregation of cells	2.38E-06	-0.353	10
31	Cellular Movement. Immune Cell Trafficking	leukocyte migration	5.55E-05	-0.348	14
32	Cellular Movement	cell movement	7.70E-07	-0.304	27
33	Molecular Transport	transport of molecule	1.88E-05	-0.262	21
34	Cardiovascular Disease	vascular lesion	3.49E-05	-0.243	7
35	Cellular Movement, Hematological System Development and Function, Immune Cell Trafficking	cell movement of leukocytes	5.75E-05	-0.239	13
36	Cellular Movement, Hematological System Development and Function, Immune Cell Trafficking, Inflammatory Response	cell movement of phagocytes	2.04E-04	-0.227	10
37	Cellular Assembly and Organization	quantity of filaments	5.04E-07	-0.218	7
38	Cellular Movement, Hematological System Development and Function, Immune Cell Trafficking	cell movement of myeloid cells	1.91E-04	-0.211	10
39	Cellular Movement, Hematological System Development and Function, Immune Cell Trafficking, Inflammatory Response	cell movement of neutrophils	2.96E-04	-0.115	7
40	Cell-To-Cell Signaling and Interaction, Inflammatory Response	immune response of macrophages	2.50E-03	0	4
41	Cellular Movement, Hair and Skin Development and Function	cell movement of epithelial cell lines	7.85E-04	0	4
42	Gastrointestinal Disease, Hepatic System Disease, Liver Damage, Organismal Injury and Abnormalities	damage of liver	1.04E-03	0	6
43	Cardiovascular Disease	atherosclerotic lesion	2.91E-05	0.13	6
44	Carbohydrate Metabolism	activation of carbohydrate	2.37E-07	0.152	4
45	Hematological System Development and Function	hemostasas	3.31E-08	0.191	10
46	Hematological System Development and Function	coagulation	1.26E-07	0.192	9
47	Cell-To-Cell Signaling and Interaction, Cellular Function and Maintenance, Hematological System Development and Function, Inflammatory Res	phagocytosis of myeloid cells	1.89E-03	*0.278*	4
48	Cell-To-Cell Signaling and Interaction, Cellular Function and Maintenance, Inflammatoiy Response	phagocytosis of phagocytes	1.89E-03	0.278	4
49	Cell-To-Cell Signaling and Interaction, Hematological System Development and Function, Inflammatory Response	aggregation of blood platelets	2.26E-05	0.335	9
50	Cell-To-Cell Signaling and Interaction	adhesion of blood cells	5.67E-05	0.349	9
51	Cell-To-Cell Signaling and Interaction, Hematological System Development and Function, Immune Cell Trafficking	adhesion of immune cells	1.63E-04	0.506	8
52	Cell-To-Cell Signaling and Interaction, Inflammatory Response	immune response of phagocytes	1.28E-01	0.603	5
53	Cell-To-Cell Signaling and Interaction	response of mycloid cells	1.58E-03	0.603	5
54	Cardiovascular Disease. Hematological Disease	Thrombosis	4.92E-05	0.613	6
55	Cardiovascular Disease	Infarction	6.11E-04	0.686	7
56	Cardiovascular Disease, Organisrnal Injury and Abnormalities, Tissue Morphology	size of infarct	4.57E-03	0.686	4
57	Cell-To-Cell Signaling and Interaction, Cellular Function and Maintenance, Inflammatory Response	phagocytosis of cells	1.17E-03	0.789	6
58	Organismal Injury and Abnormalities, Tissue Morphology	size of lesion	6.60E-04	1	6
59	Cellular Function and Maintenance	engulfment of cells	2.27E-03	1.109	7
60	Embryonic Development. Organismal Development. Tissue Development	growth of embryonic tissue	4.94E-03	1.131	5

Table S5. IPA-identified canonical pathways in septic patients.

S. No.	Name of Canonical Pathway	No. of molecules	Total molecules	Ratio	Z Score	(-)LOG B-H-p value	B-H-p value
IPA identified canonical pathways in septic survivor patients at day 0 (D0S).							
1	LXR/RXR Activation	15	128	0.117	-2.58	17.476	3.34E-18
2	Acute Phase Response Signaling	16	171	0.094	2.828	17.476	3.34E-18
3	FXR/RXR Signaling	14	138	0.101	NaN	15.711	1.95E-16
4	IL-12 Signaling and Production in Macrophages	11	138	0.08	NaN	11	1E-11
5	Clathrin-mediated Endocytosis Signaling	12	188	0.064	NaN	11	1E-11
6	Atherosclerosis Signaling	10	125	0.08	NaN	10.006	9.85E-11
7	Production of Nitric Oxide and Reactive Oxygen Species in Macrophages	11	186	0.059	-0.905	9.753	1.76E-10
8	Coagulation System	7	35	0.2	0.378	9.695	2.02E-10
9	Intrinsic Prothrombin Activation Pathway	5	30	0.167	NaN	6.273	5.33E-07
10	Extrinsic Prothrombin Activation Pathway	4	18	0.222	1	5.415	3.84E-06
11	Actin Cytoskeleton Signaling	7	221	0.032	1.342	4.18	6.61E-05
12	Role of Tissue Factor in Cancer	5	112	0.045	NaN	3.505	0.000313
13	TR/RXR Activation	4	92	0.043	NaN	2.639	0.0023
14	Hepatic Fibrosis/Hepatic Stellate Cell Activation	4	187	0.021	NaN	1.515	0.0306
IPA identified canonical pathways in septic non-survivor patients at day 0 (D0NS).							
1	Acute Phase Response Signaling	17	171	0.099	3	19.068	8.56E-20
2	LXR/RXR Activation	15	128	0.117	-0.258	17.995	1.01E-18
3	FXR/RXR Signaling	14	138	0.101	NaN	15.96	1.1E-16
4	Clathrin-mediated Endocytosis Signaling	11	188	0.059	NaN	9.847	1.42E-10
5	Coagulation System	7	35	0.2	0.378	9.847	1.42E-10
6	Atherosclerosis Signaling	9	125	0.072	NaN	8.803	1.57E-09
7	IL-12 Signaling and Production in Macrophages	9	138	0.065	NaN	8.484	3.28E-09
8	Extrinsic Prothrombin Activation Pathway	5	18	0.278	0.447	7.816	1.53E-08
9	Production of Nitric Oxide and Reactive Oxygen Species in Macrophages	9	186	0.048	-1	7.446	3.58E-08
10	Intrinsic Prothrombin Activation Pathway	5	30	0.167	NaN	6.704	1.98E-07
11	Role of Tissue Factor in Cancer	4	112	0.036	NaN	2.64	0.00299
12	Actin Cytoskeleton Signaling	5	221	0.023	1	2.507	0.00311
13	TR/RXR Activation	3	92	0.033	NaN	1.845	0.0143
IPA identified canonical pathways in septic survival patients after day 7 (D7S).							
1	Acute Phase Response Signaling	16	171	0.094	3.162	15.722	1.9E-16
2	LXR/RXR Activation	13	128	0.102	2.77	13.11	7.76E-14
3	FXR/RXR Signaling	12	138	0.087	NaN	11.309	4.91E-12
4	Clathrin-mediated Endocytosis Signaling	11	188	0.059	NaN	8.469	3.39E-09
5	Coagulation System	6	35	0.171	0.816	6.932	1.17E-07
6	IL-12 Signaling and Production in Macrophages	8	138	0.058	NaN	5.914	1.22E-06
7	Atherosclerosis Signaling	7	125	0.056	NaN	5.037	9.19E-06
8	Production of Nitric Oxide and Reactive Oxygen Species in Macrophages	8	186	0.043	-0.707	5.037	9.19E-06
9	Extrinsic Prothrombin Activation Pathway	4	18	0.222	1	4.924	1.19E-05
10	Actin Cytoskeleton Signaling	8	221	0.036	1.633	4.566	2.72E-05
11	Intrinsic Prothrombin Activation Pathway	4	30	0.133	NaN	4.073	8.45E-05
12	Role of Tissue Factor in Cancer	5	112	0.045	NaN	2.999	0.001
13	Germ Cell-Sertoli Cell Junction Signaling	5	163	0.031	NaN	2.266	0.00542
14	TR/RXR Activation	4	92	0.043	NaN	2.24	0.00575
15	MSP-RON Signaling Pathway	3	47	0.064	NaN	1.978	0.0105
16	Epithilial Adherens Junction Signaling	4	148	0.027	NaN	1.525	0.0298
17	Role of BRCA1 in DNA Damage Response	3	78	0.038	NaN	1.395	0.0403
IPA identified canonical pathways in septic non-survivor patients after day 7 (D7NS).							
1	LXR/RXR Activation	12	128	0.094	-0.577	11.841	1.44E-12
2	Acute Phase Response Signaling	13	171	0.076	2.646	11.841	1.44E-12
3	FXR/RXR Signaling	11	138	0.08	NaN	10.129	7.43E-11
4	Coagulation System	7	35	0.2	0.378	8.906	1.24E-09
5	Intrinsic Prothrombin Activation Pathway	6	30	0.2	NaN	7.526	2.98E-08
6	Extrinsic Prothrombin Activation Pathway	5	18	0.278	1	6.914	1.22E-07
7	Clathrin-mediated Endocytosis Signaling	8	188	0.043	NaN	5.157	6.97E-06
8	IL-12 Signaling and Production in Macrophages	6	138	0.043	NaN	3.689	0.000205
9	Actin Cytoskeleton Signaling	7	221	0.032	1.342	3.626	0.000236
10	Production of Nitric Oxide and Reactive Oxygen Species in Macrophages	6	186	0.032	-1.633	3.085	0.000822
11	Role of Tissue Factor in Cancer	5	112	0.045	NaN	3.085	0.000822
12	Atherosclerosis Signaling	5	125	0.04	NaN	2.895	0.00127
13	TR/RXR Activation	4	92	0.043	NaN	2.303	0.00498
14	MSP-RON Signaling Pathway	3	47	0.064	NaN	2.016	0.00964
